# Targeting the SMAD3/CISD2 axis suppresses bladder cancer progression by promoting ferroptosis in mesenchymal-like bladder cancer cells

**DOI:** 10.1038/s41419-025-08339-9

**Published:** 2025-12-18

**Authors:** Yulin Sun, Jianghui Wang, Da Gu, Huanmin Lou, Jinpeng Sun, Zilian Cui, Weiting Kang

**Affiliations:** 1 Department of Urology, Shandong Provincial Hospital Affiliated to Shandong First Medical University, Jinan, Shandong China; 2 Department of Orthopedic Surgery, The Second People’s Hospital of Dongying, Dongying, Shandong China; 3 Department of Plastic Surgery, Central Hospital Affiliated to Shandong First Medical University, Jinan, Shandong China

**Keywords:** Bladder cancer, Apoptosis, Transcriptional regulatory elements

## Abstract

The poor prognosis of bladder cancer (BCa) is primarily attributed to the acquisition of invasive and metastatic capabilities by tumor cells through epithelial-mesenchymal transition (EMT), yet the dynamic alterations in ferroptosis during EMT and their regulatory mechanisms remain unelucidated. This study is the first to reveal the mechanism by which the Smad3/CISD2 signaling axis regulates ferroptosis in mesenchymal-like bladder cancer cells. Clinical sample analysis demonstrated significantly reduced expression of E-cadherin and upregulation of N-cadherin and Vimentin in muscle-invasive bladder cancer tissues (MIBC), with EMT-related marker levels correlating with overall survival rates in BCa patients. In TGF-β1-induced mesenchymal-like bladder cancer cells, ferroptosis-related genes (GPX4, SLC7A11) were markedly elevated, alongside increased lipid peroxides (LPO) and glutathione (GSH) levels. However, mesenchymal-like bladder cancer cells exhibited heightened sensitivity to the ferroptosis inducer Erastin, showing more pronounced suppression of proliferation, elevated ROS, higher LPO and MDA levels, and reduced GSH, confirming their enhanced susceptibility to ferroptosis. RNA-seq revealed significant downregulation of Smad3 in ferroptosis inducer treated mesenchymal-like bladder cancer cells. Smad3 knockdown further exacerbated ferroptosis markers (elevated Fe²⁺, ROS, LPO; decreased GSH), inhibited migration, invasion, and proliferation—phenotypes reversible by ferroptosis inhibitors. Mechanistically, ChIP-seq combined with RNA-seq demonstrated that Smad3 regulates CISD2 expression by binding its promoter region, with clinical specimens confirming a positive correlation between Smad3 and CISD2 expression. Functional rescue experiments showed that CISD2 overexpression in Smad3-knockdown mesenchymal-like bladder cancer cells reversed abnormal increases in Fe²⁺, ROS, LPO, and MDA while restoring GSH levels, indicating that Smad3 modulates ferroptosis through a CISD2-dependent pathway. In vivo experiments further demonstrated that targeting the Smad3/CISD2 axis significantly suppressed xenograft tumor growth and activated ferroptosis. In conclusion, this study elucidates a novel mechanism by which the Smad3/CISD2 axis dynamically regulates ferroptosis through redox homeostasis reprogramming during EMT, providing a potential therapeutic strategy for targeting the progression of MIBC.

## Introduction

Bladder cancer (BCa) is one of the most common malignant tumors in the urinary system, ranking 10th in global incidence and 13th in mortality among systemic malignancies, posing a serious threat to patient survival [1]. Based on tumor invasion depth, BCa can be further classified into non-muscle-invasive bladder cancer (NMIBC) and muscle-invasive bladder cancer (MIBC) [2]. Clinical studies demonstrate that the 5-year survival rate for NMIBC patients can reach as high as 90%, while this rate plummets to 40%-60% for MIBC patients [3]. This significant prognostic disparity is not only attributed to increased surgical difficulty and elevated postoperative recurrence risk caused by deeper tumor infiltration, but also closely associated with MIBC’s resistance to conventional radiotherapy/chemotherapy and the limitations of current targeted therapeutic regimens [3–6**]**. Current MIBC treatment primarily relies on neoadjuvant chemotherapy combined with radical cystectomy, yet patients’ long-term survival benefits remain limited due to the lack of highly effective specific molecular targets [3–6**]**. Therefore, elucidating the key molecular mechanisms underlying BCa progression and exploring novel therapeutic targets hold crucial clinical significance for improving patient prognosis and developing precision therapeutic strategies.

The epithelial-mesenchymal transition (EMT), as a central biological event driving invasion and metastasis in BCa and other solid tumors, displays marked heterogeneity [7–9]. EMT not only confers tumor cells with migratory and invasive capabilities through downregulation of epithelial markers such as E-cadherin and activation of mesenchymal markers like Vimentin and N-cadherin, but also remodels redox homeostasis through epigenetic and transcriptional reprogramming [9–11]. Within the dynamic tumor microenvironment (TME) of BCa, EMT interacts with stromal components—including cancer-associated fibroblasts (CAFs), immune cells, and extracellular matrix (ECM) remodeling—to foster immunosuppression, angiogenesis, and therapy resistance [12**–**14]. Notably, TGF-β/Smad signaling pathway-driven EMT progression may influence cell fate decisions by remodeling intracellular redox homeostasis, representing a critical alteration in BCa malignant progression, though the precise regulatory mechanisms remain undefined [15].

In recent years, ferroptosis, a novel form of programmed cell death, has garnered widespread attention, characterized by redox homeostasis collapse: iron-dependent lipid peroxides (LPO) accumulation and glutathione peroxidase 4 (GPX4) system inactivation [16**–**18]. Notably, the redox homeostasis remodeling accompanying EMT may directly regulate tumor cell sensitivity to ferroptosis [19]. Emerging studies demonstrate that EMT significantly enhances tumor cell ferroptosis susceptibility through multiple mechanisms in breast cancer and colorectal cancer [20, 21]. However, in BCa, how EMT-mediated redox system remodeling affects ferroptosis response remains undefined.

This study focuses on redox homeostasis remodeling under EMT context in BCa, and proposes the novel concept that the Smad3/CISD2 axis serves as a critical node regulating ferroptosis. By integrating multi-omics sequencing data with functional experimental validation, we will systematically elucidate the molecular mechanisms of Smad3-mediated ferroptosis regulation in EMT settings. This investigation not only aims to uncover novel mechanisms underlying BCa malignant progression such as invasion and metastasis, but also provides theoretical foundations for developing ferroptosis-based precision therapeutic strategies, holding significant translational value for improving prognosis in BCa patients.

## Materials and methods

### Clinical specimens

All BCa tissue samples and their matched adjacent normal tissues used in this study were obtained with approval from the Medical Ethics Committee of Shandong First Medical University Affiliated Provincial Hospital. This study strictly adhered to the ethical principles of the Declaration of Helsinki, and written informed consent was obtained from all participants.

### Cell culture and EMT model

The human BCa cell lines T24 and 5637 were purchased from Suzhou Haixing Biosciences Co., Ltd. All cell lines were authenticated by short tandem repeat (STR) profiling and confirmed to be mycoplasma-free (testing results were negative) prior to experiments to ensure cell quality met experimental requirements. Cell culture strictly followed supplier-recommended protocols: T24 cells were maintained in DMEM complete medium (Gibco, 10566016) supplemented with 10% fetal bovine serum (Vivacell, C04001-500) and 1% penicillin-streptomycin solution (Solarbio, P1400); 5637 cells were cultured in RPMI-1640 complete medium (Gibco, 11875093) with identical concentrations of serum and antibiotics. All cells were routinely passaged in a humidified incubator at 37 °C with 5% CO₂.

To establish the EMT model, experimental group cells were treated with recombinant human TGF-β1 at a fixed concentration of 10 ng/mL. The culture medium containing 10 ng/mL TGF-β1 was refreshed every 48 h, and the treatment continued for 4 days. The control group was cultured under identical conditions without TGF-β1 supplementation.

### Cell transfection

Cell transfection was performed using the Polyplus jetPRIME® transfection kit (Polyplus, 101000046) strictly following the manufacturer’s protocol. For each well of a 6-well plate, 2000 ng plasmid DNA or 50 nM small interfering RNA (siRNA) was dissolved in jetPRIME® buffer, then mixed with jetPRIME® transfection reagent to form nucleic acid-polymer complexes. The complexes were evenly distributed by dropwise addition onto the cell culture medium surface, followed by gentle swirling of the culture plate to ensure uniform distribution.

### Chemicals and antibodies

Chemical reagents used in this study included: recombinant human transforming growth factor β1 (TGF-β1, HY-P78168), ferroptosis inducers Erastin (HY-15763) and RSL3 (HY-100218A), ferroptosis inhibitor Ferrostatin-1 (Fer-1, HY-100579), and iron chelator Deferoxamine mesylate (DFO, HY-B0988), all purchased from MedChemExpress (MCE) with purity ≥98%. Stock solutions were prepared according to the manufacturer’s instructions prior to use.

Bladder cancer cells were first treated with 10 ng/mL TGF-β1 for ≥4 days to establish mesenchymal-like phenotypes. To induce ferroptosis in these mesenchymal-like bladder cancer cells, the cells were then treated with either 0.2 μM RSL3 or 2 μM Erastin for 12 h. For ferroptosis inhibition, 2 μM Fer-1 was co-administered with RSL3 or Erastin, and maintained throughout the treatment. In experiments measuring intracellular Fe²⁺ levels, cells were pretreated with 50 μM DFO for 4 h before inducer exposure, and DFO treatment was continued during ferroptosis induction.

Antibody information: The following primary antibodies were used for western blot, IHC, and IF experiments: GPX4 (Proteintech, 67763-1-Ig, 1:1000), SLC7A11 (Proteintech, 26864-1-AP, 1:1000), E-cadherin (Proteintech, 20874-1-AP, 1:30000), Vimentin (Proteintech, 60330-1-Ig, 1:20000), N-cadherin (Proteintech, 22018-1-AP, 1:20000), Smad3 (Abcam, ab40854, 1:4000), p-Smad3 (Abcam, ab52903, 1:2000), CISD2 (Cell Signaling technology, 60758S, 1:1000), and GAPDH (Proteintech, 60004-1-Ig, 1:10000). Secondary antibody information: WB secondary antibodies: HRP-conjugated goat anti-mouse/rabbit IgG (Proteintech, SA00001-1/2, 1:10000); IHC secondary antibodies: rabbit/mouse HRP detection kit (ZSGB-BIO, PV-9000); IF secondary antibodies: Alexa Fluor® 488-conjugated goat anti-rabbit IgG (H&L) (Abcam, ab150077, 1:200) and Alexa Fluor® 594-conjugated goat anti-mouse IgG (H&L) (Abcam, ab150113, 1:200).

### RNA extraction and RT-qPCR

Total RNA was extracted using TRIzol reagent (Accurate Biotechnology, AG21102) following the manufacturer’s instructions. Cells in logarithmic growth phase were lysed with TRIzol, followed by chloroform extraction, isopropanol precipitation, and 75% ethanol washes, with final dissolution in RNase-free water. RNA concentration and purity were measured using a NanoDrop One spectrophotometer (Thermo Fisher Scientific). cDNA synthesis was performed using the Evo M-MLV Reverse Transcription Premix Kit (Accurate Biotechnology, AG11705). RT-qPCR was conducted using SYBR Green master mix (Accurate Biotechnology, AG11739) on a LightCycler 480 system (Roche). GAPDH served as the internal reference gene, and relative expression of target genes was calculated using the 2−ΔΔCt method.

### Western blot

Protein extraction: Cells were lysed with ice-cold RIPA buffer (Solarbio, 240003001) supplemented with 1% PMSF (Solarbio, P0100-01) and 1% protease/phosphatase inhibitor cocktail (New Cell & Molecular Biotech, P002). The lysates were sonicated and centrifuged to collect supernatants. Protein concentrations were determined using a BCA protein assay kit (Solarbio, PC0021).

Western blot analysis: Protein samples were separated by 10% SDS-PAGE and transferred to PVDF membranes (Millipore, ISEQ00010) using a wet transfer system (Bio-Rad). After blocking with 5% skim milk (Solarbio, D8340), the membranes were incubated with appropriately diluted primary antibodies overnight at 4 °C, followed by incubation with HRP-conjugated goat anti-mouse/rabbit IgG secondary antibodies (Proteintech, SA00001-1/SA00001-2) for 1 h at room temperature. Protein bands were visualized using ECL substrate (Epizyme, SQ203L-1) and imaged with a ChemiDoc MP system (Bio-Rad).

### Cell viability

Cell viability assay was performed using the CCK-8 kit (NewCell & Molecular Biotech, C6005). Cells were seeded in 96-well plates and subjected to respective treatments (e.g., drug stimulation, transfection). Following the manufacturer’s protocol, 10 μL of CCK-8 solution and 100 μL of serum-free medium were added to each well, gently mixed, and incubated at 37 °C with 5% CO₂ in the dark for 1 hour. Absorbance (OD value) was measured at 450 nm using a microplate reader (Molecular Devices) to calculate cell viability.

### Colony formation

Cells were seeded in six-well plates and continuously cultured in a 37 °C, 5% CO₂ incubator for 14 days, with fresh medium replaced every 72 h. When visible cell colonies (≥0.5 mm in diameter) were observed, the culture was terminated and 1 mL of 4% paraformaldehyde (Solarbio, P1110) was added to each well for 20-min fixation at room temperature. Subsequently, 1 mL of 1% crystal violet staining solution (Bioss, S0286) was added to each well for 20-min staining at room temperature, followed by three gentle washes with distilled water to remove background staining. After air-drying, the plates were scanned and imaged using a scanner (Canon).

### Construction of stably Smad3-silenced expressed cells

The CRISPR-Cas9 and overexpression lentiviral vectors were constructed by Keyybio. Cells were seeded in six-well plates, and after 24 h, lentiviral particles were added. The medium was replaced with complete medium containing 10% fetal bovine serum 6 h post-infection. At 48 h after infection, puromycin (Sigma, P8833) was added for selection, which continued for 2 weeks until all uninfected cells died.

### Tissue immunohistochemistry (IHC) and immunofluorescence(IF)

*Tissue sample preparation*: Normal adjacent tissues, tumor tissues from BCa patients, and mouse subcutaneous xenograft tissues were collected. After fixation with 4% paraformaldehyde, the samples were routinely paraffin-embedded and sectioned. Sections were baked at 60 °C for 2 h, followed by dewaxing in xylene and hydration through a graded ethanol series. Antigen retrieval was performed using citrate buffer (pH 6.0; Solarbio, C0090).

*IHC staining*: Sections were incubated with 3% hydrogen peroxide at room temperature for 15 min to block endogenous peroxidase activity. Primary antibodies were applied and incubated overnight at 4 °C, followed by incubation with HRP-conjugated secondary antibodies (ZSGB-BIO, PV-9000) at room temperature for 1 h. DAB substrate (Beyotime, P0203) was used for color development, followed by hematoxylin counterstaining and mounting with neutral resin. Images were acquired using a Leica DMi8 upright microscope.

*IF staining*: After dewaxing, hydration, and antigen retrieval, sections were permeabilized with 0.5% Triton X-100/PBS (containing 0.5% v/v Triton X-100, Beyotime, P0096) at room temperature for 10 minutes. Blocking was performed with 10% goat serum albumin (Beyotime, C0265) in PBS for 30 min at room temperature. Primary antibodies were incubated overnight at 4 °C, followed by species-specific fluorescent secondary antibodies (Goat Anti-Rabbit/Mouse IgG H&L Alexa Fluor® 488/594, Abcam, ab150077/ab150113, dilution 1:200) for 1 h in the dark. Nuclei were stained with DAPI (Beyotime, C1005), and sections were mounted with antifade mounting medium (Beyotime, P0126). Images were acquired using a Leica TCS SP8 confocal microscope.

### Chromatin immunoprecipitation sequencing (ChIP-seq)

The ChIP assay was performed using the SimpleChIP® Plus kit (Cell Signaling Technology) following manufacturer’s instructions. DNA quality was verified by electrophoresis and quantified using Qubit® fluorometry. Libraries were prepared from 50 ng DNA with NEBNext® Ultra™ kit and sequenced on Illumina platform (2×150 bp). Bioinformatics analysis included read alignment (Bowtie 2), peak calling (MACS2), motif analysis (Homer), and data visualization (deepTools, ChIPseeker).

### RNA sequencing

Total RNA was extracted using TRIzol reagent, with quality verified by NanoDrop and Bioanalyzer (RIN > 7.0). Poly(A) RNA was purified, fragmented, and converted to cDNA libraries using NEBNext reagents, followed by 150 bp paired-end sequencing on Illumina Novaseq 6000. The raw sequencing data have been deposited in the NCBI Sequence Read Archive (SRA) under BioProject accession number PRJNA1336318. Bioinformatics analysis included quality control (fastp), alignment to GRCh38 (HISAT2), transcript assembly (StringTie), and differential expression analysis (edgeR).

### ROS detection

Cells were seeded in six-well plates and grouped according to experimental design. After treatment, cells were collected by digestion with 0.25% trypsin (Gibco, 25200056), washed twice with PBS, and then incubated with 10 μM DCFH-DA probe (Beyotime, S0033S) at 37 °C with 5% CO₂ in the dark for 30 min. The DCFH-DA probe is a non-specific fluorescent dye that detects total intracellular ROS levels (including H_2_O_2_, hydroxyl radicals, and peroxynitrite) rather than organelle-specific ROS (e.g., mitochondrial ROS). Following incubation, cells were washed twice with serum-free medium and resuspended in PBS, and fluorescence intensity was immediately measured using a FACSCanto II flow cytometer (BD Biosciences).

### Staining of intracellular ferrous iron (Fe^2+^)

Cells were seeded in glass-bottom confocal dishes (Biosharp, P24000040259) and grouped according to experimental design. After washing with PBS, cells were incubated with FerroOrange fluorescent probe (Dojindo, F374) at 37 °C in the dark for 30 min to allow specific binding of the probe to intracellular Fe^2+^. Following incubation, cells were immediately imaged using a Leica TCS SP8 confocal microscope.

### Transmission electron microscopy (TEM)

The cells were collected by centrifugation after treatment and fixed with electron microscopy fixative at 4 °C. Subsequently, the samples were washed with phosphate buffer (PB) and post-fixed with 1% osmium tetroxide (OsO_4_) under light-protected conditions, followed by multiple washes with PB and ultrapure water. The samples were then dehydrated through a graded ethanol and acetone series, infiltrated with an acetone-Epon812 resin (SPI) mixture, and embedded in pure resin within molds. After polymerization, the resin blocks were trimmed, and ultrathin sections were cut and collected on 150-mesh copper grids. The sections were stained with 2% uranyl acetate and 2.6% lead citrate, followed by observation and image acquisition using a transmission electron microscope (Hitachi HT-7800, 80 kV).

### C11-BODIPY staining

Cells were seeded in glass-bottom confocal dishes (Biosharp, P24000040259) and grouped according to experimental design. After PBS washing, cells were incubated with C11-BODIPY 581/591 fluorescent probe (Beyotime, S0043S) at 37 °C in the dark for 20 min. Following incubation, cells were washed twice with PBS and immediately imaged using a Leica TCS SP8 confocal microscope. Both unoxidized probe (red fluorescence, emission wavelength 591 nm) and oxidation products (green fluorescence, emission wavelength 510 nm) were detected.

### Reduced glutathione (GSH), lipid peroxidation (LPO), and malondialdehyde (MDA) assay

*Determination of GSH*: After centrifugation to collect cells, the supernatant was removed and cells were resuspended in Reagent I, followed by ice-bath sonication. The supernatant was then obtained by centrifugation. GSH content was determined by measuring absorbance at 412 nm according to the manufacturer’s protocol of the GSH assay kit (Solarbio, BC1175).

*Determination of LPO*: Cells were collected by centrifugation and resuspended in extraction buffer after supernatant removal, followed by ice-bath sonication. The supernatant was collected by centrifugation. LPO content was measured by detecting absorbance at 532 nm and 600 nm using the LPO assay kit (Solarbio, BC5245).

*Determination of MDA*: Following cell collection via centrifugation and supernatant removal, cells were resuspended in extraction buffer and subjected to ice-bath sonication. The supernatant was then obtained by centrifugation. MDA content was quantified by measuring absorbance at 532 nm and 600 nm according to the MDA assay kit (Solarbio, BC0025) instructions.

### Cell migration and invasion assays

*Migration assay*: Cell migration ability was evaluated using transwell chambers (24-well plate format, 8 μm pore size, Corning, 3422). After respective treatments, cells were seeded in the upper chamber, while complete medium containing 20% fetal bovine serum was added to the lower chamber as a chemoattractant. The chambers were incubated at 37 °C with 5% CO₂ for 24-48 h. Following incubation, migrated cells were fixed with 4% paraformaldehyde (Solarbio, P1110) for 20 min at room temperature. Non-migrated cells on the upper surface of the membrane were carefully removed using a cotton swab, while migrated cells on the lower surface were stained with 0.1% crystal violet solution (Bioss, S0286) at room temperature. The membranes were then examined under an inverted microscope (Leica).

*Invasion assay*: The invasion assay procedure was similar to the migration assay, with the exception that Transwell chambers were pre-coated with Matrigel matrix before cell seeding. Appropriately diluted Matrigel matrix (Corning, 354234) was evenly applied to the upper chamber surface and incubated at 37 °C for 4 h to form an extracellular matrix barrier.

### Wound healing assay

Cells in the logarithmic growth phase were seeded into six-well plates and cultured in medium supplemented with 10% fetal bovine serum until reaching near-confluence (90–100%). Straight wounds were created by scraping the cell monolayer with a 1 mL sterile pipette tip perpendicular to the bottom of the plate, followed by gentle washing with PBS three times to remove detached cells. The medium was then replaced with serum-free medium, and cells were further incubated at 37 °C with 5% CO₂. Wound areas were photographed under an inverted microscope at 0, 24, 48, and 72 h. The wound widths were quantitatively analyzed using ImageJ software (NIH), and cell migration rates were calculated accordingly.

### Tumour xenograft experiment

*Animal Ethics*: This study strictly followed the protocol approved by the Animal Ethics Committee of Shandong Provincial Hospital Affiliated to Shandong First Medical University, and complied with the ethical guidelines of the Guide for the Care and Use of Laboratory Animals. Four-week-old female BALB/c nude mice (Beijing Vitalever Laboratory Animal Technology) were used in this study, with six mice allocated to each experimental group. All mice had identical age, sex, and genetic background at the start of the experiment. Following respective treatments, cells from different experimental groups were resuspended in a 1:1 mixture of Matrigel matrix (Corning, 354248) and PBS at a final concentration of 2 × 10⁶ cells/100 μL, and then subcutaneously injected into the right flank of nude mice (injection volume: 100 μL, 27 G needle). The treatment group received Fer-1 (10 mg/kg, dissolved in DMSO/PBS system) via intraperitoneal injection every 2 days for 21 days. Tumor length (*L*) and width (*W*) were measured every 3 days, and tumor volume was calculated using the formula *V* = (*L* × *W*²)/2, while mouse body weight was recorded simultaneously. After 28 days, euthanasia was performed in accordance with institutional guidelines. Tumor weight and maximum/minimum diameters were measured. Following photographic documentation, portions of tumor tissues were fixed in 4% paraformaldehyde for section staining.

### Statistical analysis

Statistical analyses were performed using GraphPad Prism 8.0 software (GraphPad Software, USA). Experiments were repeated independently at least three times. Student’s *t* test one-way ANOVA was used to determine the significance of two and multiple groups, respectively. Two-way ANOVA was used to analyze the differences between the two groups over time. Survival analysis was performed using the Kaplan-Meier method to generate survival curves, with between-group differences assessed by Log-rank test. Data are expressed as mean ± mean square deviation. *p* < 0.05 statistically significant (ns, not significant, *p < 0.05, **p < 0.01, ***p < 0.001, ****p < 0.0001).

## Results

### Activation of EMT is associated with BCa progression and adverse patient prognosis

**Fig. 1:**
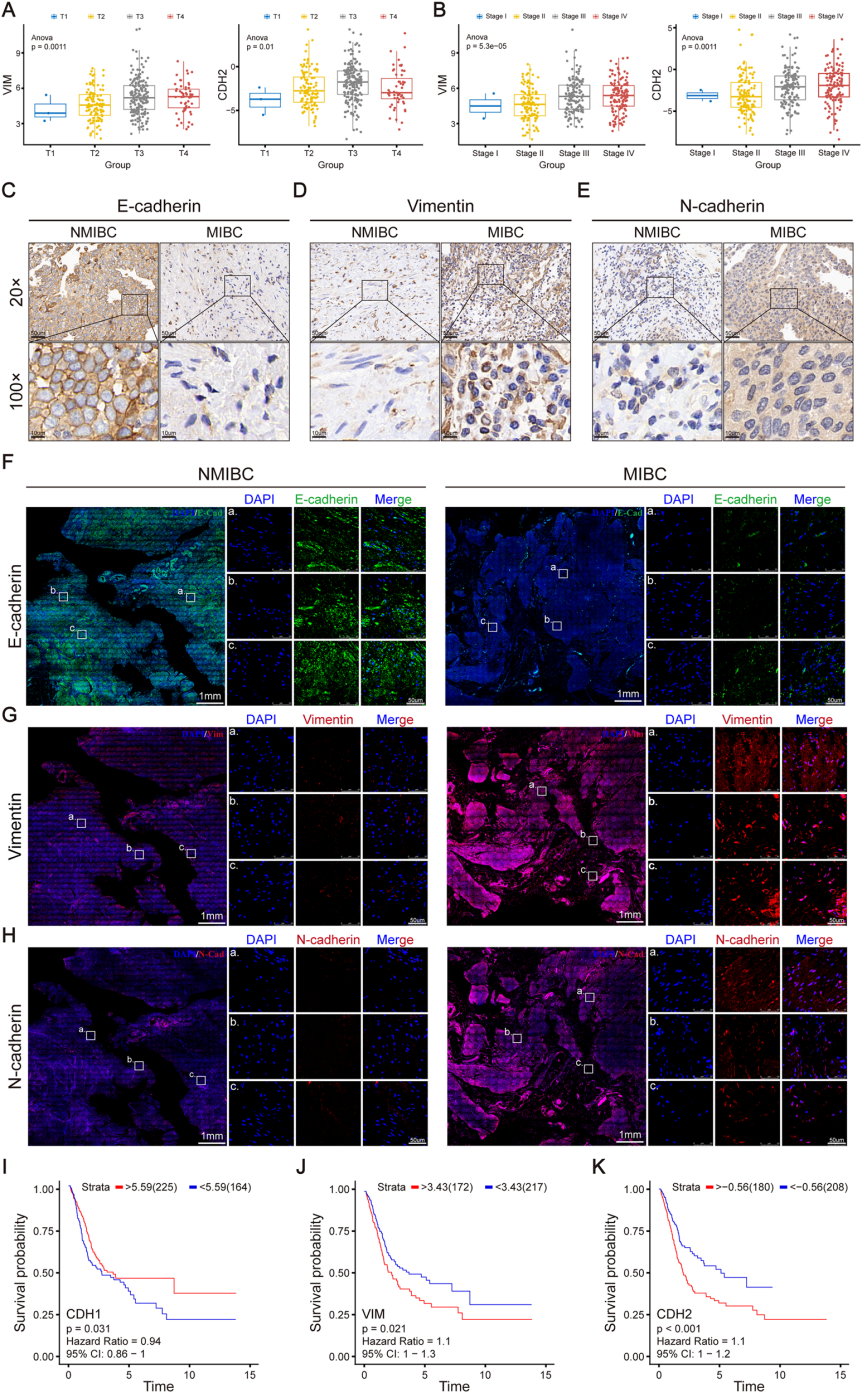
EMT activation in bladder cancer tissues correlates with poor patient prognosis. **A, B** Relative expression levels of EMT markers VIM and CDH2 across TNM stages (T1–T4) and clinical stages (Stage I–IV) in bladder cancer (BCa) from the TCGA dataset. **C–E** IHC staining of EMT markers E-cadherin, Vimentin, and N-cadherin in MIBC versus NMIBC tissues. **F–H** IF staining of EMT markers E-cadherin, Vimentin, and N-cadherin in MIBC compared to NMIBC tissues. **I–K** Kaplan-Meier survival curves showing that the expression of CDH1, VIM, and CDH2 are associated with overall survival (OS) in bladder cancer patients. Data are presented as the mean ± SEM. *p < 0.05, **p < 0.01, ***p < 0.001, ****p < 0.0001; ns, not significant (*p* > 0.05).

To investigate the relationship between EMT and BCa progression, this study analyzed mRNA expression differences of EMT characteristic markers in BCa samples from the TCGA database, comparing tumors across TNM stages (T1–T4) and clinical stage groups (Stage I–IV). Results showed that epithelial marker CDH1 mRNA expression progressively decreased with advancing clinical stages, while mesenchymal markers CDH2 and VIM mRNA levels elevated (Fig. 1A, B). These results suggest a significant positive correlation between EMT activation and tumor staging. To further validate these findings, we collected matched clinical samples of NMIBC and MIBC for IHC analysis. IHC revealed significantly decreased protein expression intensity of epithelial marker E-cadherin and markedly increased levels of mesenchymal markers N-cadherin and Vimentin in MIBC compared to NMIBC (Fig. 1C–E). IF results corroborated these findings (Fig. 1F–H). Collectively, these data confirm persistent enhancement of EMT phenotype during tumor invasion.

To evaluate the clinical significance of EMT, patients were stratified into high- and low-expression groups based on optimal cutoff values of CDH1, CDH2, and VIM expressionm, determined using the R software package “surv_cutpoint” (minprop = 0.3). Kaplan-Meier survival analysis demonstrated that high CDH2 expression (HR = 1.1, 95% CI: 1-1.2, p < 0.001) and high VIM expression (HR = 1.1, 95% CI: 1-1.3, p = 0.021) groups exhibited significantly shorter overall survival (OS) than low-expression groups, whereas high CDH1 expression correlated with better prognosis (HR = 0.94, 95% CI: 0.86-1, p = 0.031) (Fig. 1I–K). These findings underscore the pivotal role of EMT activation in BCa progression, serving as both a biological driver of invasion/metastasis and a robust predictor of adverse patient outcomes.

### EMT is associated with ferroptosis in mesenchymal-like bladder cancer cells

**Fig. 2:**
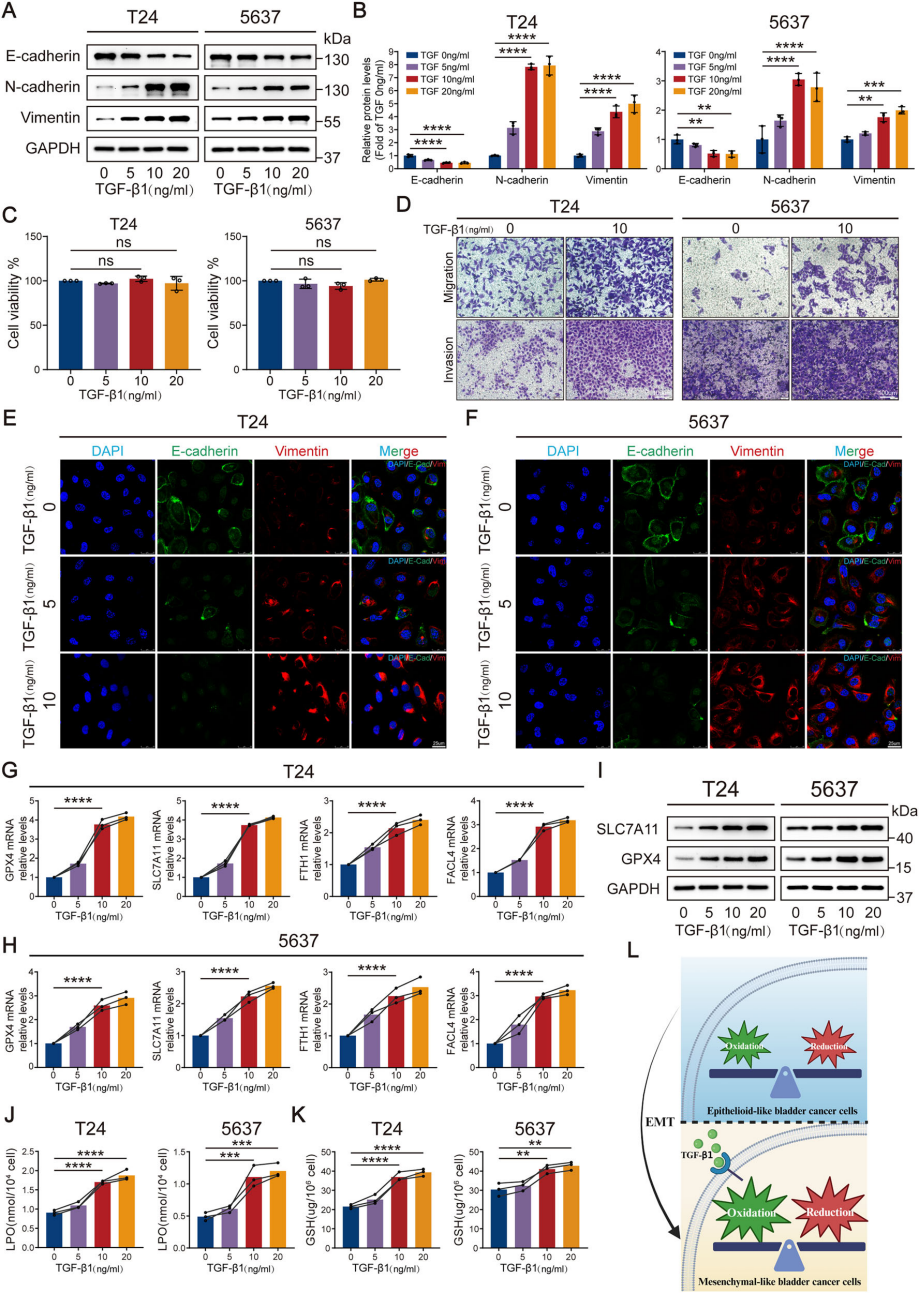
TGF-β1 induces EMT in bladder cancer cells and remodels redox homeostasis to modulate ferroptosis susceptibility. **A**, **B** Western blot confirming TGF-β1-induced suppression of epithelial markers (E-cadherin) and upregulation of mesenchymal markers (Vimentin, N-cadherin). **C** CCK-8 assay demonstrating the effect of different concentrations of TGF-β1 on cell viability. **D** Transwell migration and invasion assays demonstrated changes in migratory and invasive abilities of mesenchymal-like bladder cancer cells. **E**, **F** Immunofluorescence staining of EMT markers (E-cadherin, Vimentin) in TGF-β1-treated cells. **G**, **H** RT-qPCR profiling of TGF-β1-modulated ferroptosis-related genes (GPX4, SLC7A11, FTH1, FACL4) in cells. **I** Western blot analysis of ferroptosis suppressor proteins (GPX4, SLC7A11) in TGF-β1-treated cells. **J** LPO assay showing elevated LPO levels in TGF-β1-exposed cells. **K** GSH assay revealing TGF-β1-induced GSH depletion in cells. **L** Schematic summary of TGF-β1-driven EMT promoting redox imbalance and ferroptosis sensitivity in bladder cancer cells. Data are presented as the mean ± SEM. *p < 0.05, **p < 0.01, ***p < 0.001, ****p < 0.0001; ns, not significant (*p* > 0.05).

To investigate the role of EMT in BCa progression, based on the well-established mechanism of TGF-β/Smad pathway-mediated EMT regulation reported in the literature, we established EMT cell models by TGF-β1 induction in two human bladder cancer cell lines (T24 and 5637). Both cell lines were treated with increasing concentrations of TGF-β1 (0, 5, 10, 20 ng/mL). First, we confirmed that these TGF-β1 concentrations did not affect cell viability using the CCK-8 assay (Fig. 2C). Subsequently, we observed dose-dependent changes in EMT markers. RT-qPCR results demonstrated that epithelial marker CDH1 mRNA levels progressively decreased with increasing TGF-β1 concentrations, while mesenchymal markers CDH2 and VIM expression were significantly upregulated (Fig. S1A, B). Western blot further confirmed dynamic switching of core EMT proteins during TGF-β1-induced EMT (Fig. 2A, B). IF confocal microscopy revealed that 10 ng/mL TGF-β1 induction triggered typical mesenchymal-like morphology, characterized by Vimentin fiber network reorganization and loss of E-cadherin membrane localization (Fig. 2E, F). Based on the significance of marker changes and cellular adaptability, we identified 10 ng/mL TGF-β1 as the optimal concentration for stable EMT induction. The induced mesenchymal-like bladder cancer cell lines (T24 and 5637) were designated as T24m (mesenchymal-like T24) and 5637 m (mesenchymal-like 5637), respectively, to distinguish them from their uninduced epithelial parental cells. In conclusion, we successfully established mesenchymal-like bladder cancer cell models (T24m and 5637 m) through TGF-β1 induction, which exhibited characteristic molecular and morphological EMT alterations. Subsequent Transwell assays further validated these models by demonstrating significantly enhanced migratory and invasive capacities—hallmarks of EMT progression (Fig. 2D).

Notably, ferroptosis-related genes exhibited dynamic alterations during TGF-β1-induced EMT. RT-qPCR analysis revealed that mRNA levels of anti-ferroptosis core genes GPX4, Solute Carrier Family 7 Member 11 (SLC7A11), and Ferritin Heavy Chain 1 (FTH1) were significantly upregulated with increasing TGF-β1 concentrations, while pro-ferroptosis gene Fatty Acid-CoA Ligase 4 (FACL4) also showed synchronous elevation (Fig. 2G, H). Western blot further confirmed concentration-dependent increases in GPX4 and SLC7A11 protein expression (Fig. 2I and S1C). These results suggest that TGF-β1-induced EMT may simultaneously activate both anti-ferroptosis (e.g., GPX4, SLC7A11, FTH1) and pro-ferroptosis (e.g., FACL4) pathways, revealing a dual regulatory role of EMT in modulating ferroptosis. To decipher the biological significance of this bidirectional regulation, we measured key redox homeostasis indicators (LPO and GSH). Results demonstrated that mesenchymal-like bladder cancer cells with EMT activation showed progressively elevated LPO levels following TGF-β1 concentration escalation (Fig. 2J). Intriguingly, intracellular GSH levels simultaneously exhibited marked increases (Fig. 2K), suggesting that cells establish dynamic equilibrium through coordinated enhancement of oxidative stress response and antioxidant defense.

These findings indicate that the EMT process establishes a dynamically balanced redox microenvironment in mesenchymal-like bladder cancer cells through coordinated enhancement of antioxidant defense (GSH/GPX4 system) and oxidative stress response (Fig. 2L). These results suggest complex crosstalk between EMT and ferroptosis regulatory networks, whose specific molecular mechanisms require further validation through interventions with ferroptosis inducers (e.g., Erastin) or inhibitors (e.g., Fer-1). In summary, in vitro functional experiments confirmed that the TGF-β1-induced EMT cell model exhibits characteristic molecular phenotypic switching, accompanied by bidirectional regulation of ferroptosis-related genes and redox homeostasis remodeling, thereby establishing an experimental foundation for in-depth investigation of EMT-ferroptosis crosstalk.

### Mesenchymal-like bladder cancer cells exhibit heightened susceptibility to ferroptosis

**Fig. 3:**
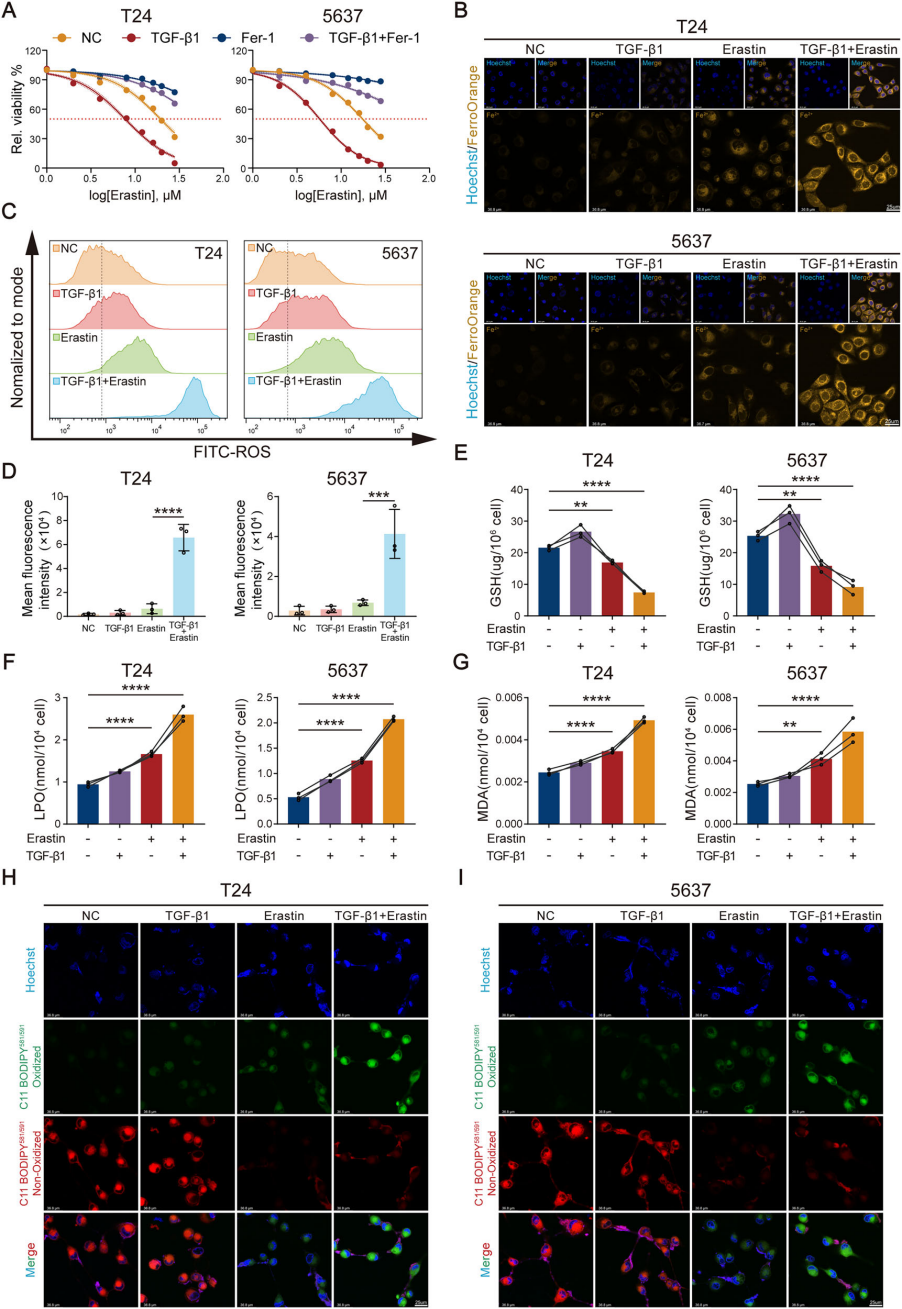
Mesenchymal-like bladder cancer cells exhibit heightened susceptibility to ferroptosis. **A** CCK-8 assay demonstrating the cell sensitivity to Erastin under different treatment conditions. **B** Ferrous iron (Fe^2+^) accumulation in cells assessed by FerroOrange fluorescence staining. **C**, **D** Flow cytometry analysis of total intracellular ROS levels. **E** Glutathione (GSH) levels quantified using a GSH assay kit. **F** Lipid peroxidation (LPO) measured via an LPO assay kit. **G** Malondialdehyde (MDA) content measured via an MDA assay kit. **H**, **I** Representative confocal images of lipid peroxidation. Red: non-oxidized lipids; Green: oxidized lipids. Data are presented as the mean ± SEM. *p < 0.05, **p < 0.01, ***p < 0.001, ****p < 0.0001; ns, not significant (*p* > 0.05).

To further elucidate the molecular mechanism by which EMT reprograms redox homeostasis to enhance ferroptosis sensitivity in BCa cells, we employed our established EMT model (10 ng/mL TGF-β1) and treated mesenchymal-like (TGF-β1-preconditioned) and epithelial-like (untreated) bladder cancer cells with the classical ferroptosis inducer Erastin, a small molecule that triggers ferroptosis by targeting inhibition of the cystine/glutamate antiporter (System Xc⁻) and GSH synthesis [22]. CCK-8 cell viability assays demonstrated that low-concentration Erastin treatment significantly suppressed the survival rate of mesenchymal-like bladder cancer cells (T24m = TGF-β1-treated group), whereas epithelial-like bladder cancer cells (T24) showed minimal viability reduction under the same conditions (Fig. 3A). These results suggest that mesenchymal-like bladder cancer cells with activated EMT exhibit significantly enhanced ferroptosis susceptibility due to their high baseline redox homeostasis. Extensive studies have confirmed that dysregulated iron metabolism (e.g., ferritinophagy and impaired iron export) leads to intracellular labile Fe²⁺ accumulation, a fundamental trigger of ferroptosis and a key catalyst for Fenton reactions, which drive ROS generation [23, 24]. To explore this core ferroptosis mechanism, we performed FerroOrange fluorescent iron staining. Confocal microscopy revealed that Erastin-treated mesenchymal-like bladder cancer cells exhibited markedly greater intracellular labile Fe²⁺ accumulation compared to epithelial-like bladder cancer cells (Fig. 3B). Excess Fe²⁺ catalyzes hydrogen peroxide (H₂O₂) decomposition into highly toxic hydroxyl radicals (·OH) via the Fenton reaction, causing ROS bursts [25]. Flow cytometry with ROS-specific probes confirmed that low-dose Erastin induced significantly elevated total intracellular ROS levels in mesenchymal-like bladder cancer cells (Fig. 3C, D). These findings collectively demonstrate that EMT-driven phenotypic switching exacerbates iron metabolism dysregulation, amplifies oxidative stress, and further support the hypothesis that mesenchymal-like bladder cancer cells are more vulnerable to ferroptosis.

We measured levels of the critical antioxidant molecule reduced GSH. Compared to epithelial-like bladder cancer cells, mesenchymal-like bladder cancer cells exhibited more pronounced intracellular GSH depletion under low-concentration Erastin induction (Fig. 3E). Further analysis of lipid peroxidation markers—LPO and MDA—revealed that mesenchymal-like bladder cancer cells generated significantly higher LPO and MDA levels than epithelial-like bladder cancer cells under equivalent Erastin treatment (Fig. 3F, G). Additionally, cellular fluorescence probe (C11-BODIPY) detection of intracellular lipid peroxidation and reduction products yielded results consistent with biochemical assays (Fig. 3H, I). This metabolic remodeling renders mesenchymal-like bladder cancer cells more prone to Erastin-induced ferroptosis, whereas epithelial-like bladder cancer cells exhibit greater resistance to ferroptosis inducers, likely due to their relatively preserved antioxidant reserves and reduced accumulation of membrane lipid peroxidation substrates compared to mesenchymal-like bladder cancer cells.

### Smad3 serves as an EMT-ferroptosis regulatory hub modulating bladder cancer cell ferroptosis

**Fig. 4:**
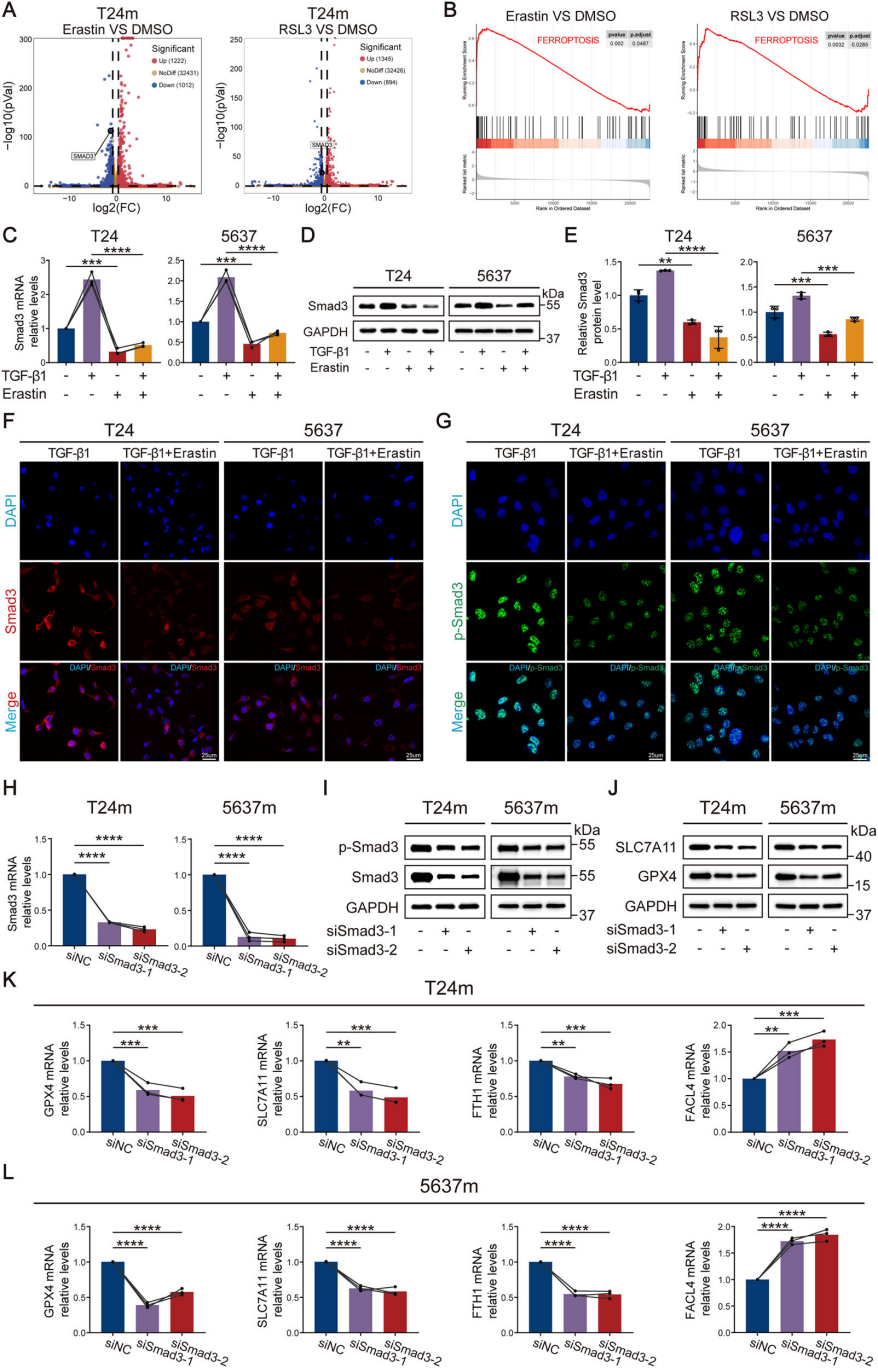
Smad3 was screened as a key gene regulating ferroptosis. **A** Volcano plots showing differentially expressed genes (DEGs) in mesenchymal-like bladder cancer cells treated with ferroptosis inducers (Erastin/RSL3) compared to DMSO controls. **B** Gene Set Enrichment Analysis (GSEA) revealed prominent enrichment of the “ferroptosis” signature. **C** RT-qPCR confirmed reduced Smad3 mRNA levels in Erastin-treated cells. **D**, **E** Western blot analysis demonstrating Erastin-induced suppression of Smad3 protein expression levels. **F**, **G** Immunofluorescence staining showing Erastin-induced alterations in Smad3 and p-Smad3 fluorescence signals. Validation of Smad3 siRNA transfection efficiency in T24m and 5637 m cells by RT-qPCR (**H**) and western blot (**I**). **J** Western blot analysis of ferroptosis suppressor protein levels in T24m and 5637 m cells following Smad3 knockdown. RT-qPCR profiling of ferroptosis-related regulatory genes in Smad3-knockdown T24m (**K**) and 5637 m (**L**) cells. Data are presented as the mean ± SEM. *p < 0.05, **p < 0.01, ***p < 0.001, ****p < 0.0001; ns, not significant (*p* > 0.05).

To comprehensively explore the molecular regulatory mechanisms of ferroptosis in mesenchymal-like bladder cancer cells, we treated mesenchymal-like T24m cells with ferroptosis inducers (Erastin and RSL3) and integrated transcriptomic profiling with functional validation to identify key regulators. RNA-seq analysis revealed 6457 differentially expressed genes (DEGs, 3154 upregulated, 3303 downregulated) between Erastin- and DMSO-treated groups, while 4630 DEGs (2372 upregulated, 2258 downregulated) were identified in RSL3-treated versus DMSO controls (Fig. 4A). Intersection analysis identified 2186 overlapping DEGs (1013 upregulated, 1173 downregulated), suggesting shared molecular underpinnings between Erastin- and RSL3-induced ferroptosis pathways. Notably, the transcription factor Smad3 was significantly downregulated in both treatment groups (*P* < 0.01). Gene Set Enrichment Analysis (GSEA) further demonstrated significant enrichment of ferroptosis core pathways in both conditions (NES = 2.1, FDR = 0.008) (Fig. 4B). RT-qPCR and western blot validation confirmed that Erastin treatment markedly reduced total Smad3 protein levels in mesenchymal-like bladder cancer cells (Fig. 4C–E). Intriguingly, TGF-β1-induced EMT upregulated Smad3 expression, whereas ferroptosis inducer treatment reversed this elevation (Fig. 4C–E). IF staining further revealed that Erastin treatment significantly reduced the cytoplasmic fluorescence signal of Smad3 (Fig. 4F), while the nuclear localization signal of phosphorylated Smad3 (p-Smad3) was also significantly reduced (Fig. 4G), indicating that both its protein stability and transcriptional activity were suppressed. Collectively, these findings suggest that Smad3 plays a pivotal role in ferroptosis regulation in mesenchymal-like bladder cancer cells.

To clarify the functional role of Smad3 in ferroptosis of mesenchymal-like bladder cancer cells, we designed two Smad3-targeting siRNAs (siSmad3-1 and siSmad3-2) to specifically knock down Smad3 expression in T24m/5637 m cells. RT-qPCR and western blot confirmed knockdown efficiencies exceeding 70% (Fig. 4H, I and S2A). RT-qPCR analysis of ferroptosis-related genes in Smad3-silenced mesenchymal-like T24m/5637 m cells revealed significant upregulation of pro-ferroptosis gene FACL4 and marked downregulation of anti-ferroptosis genes GPX4, SLC7A11, and FTH1 (Fig. 4K, L). Western blot further validated reduced protein levels of GPX4 and SLC7A11 (Fig. 4J and S2B). These results uncover the transcription factor Smad3 as a central regulatory hub orchestrating the EMT-ferroptosis network.

### Smad3 knockdown promotes ferroptosis in mesenchymal-like bladder cancer cells

**Fig. 5:**
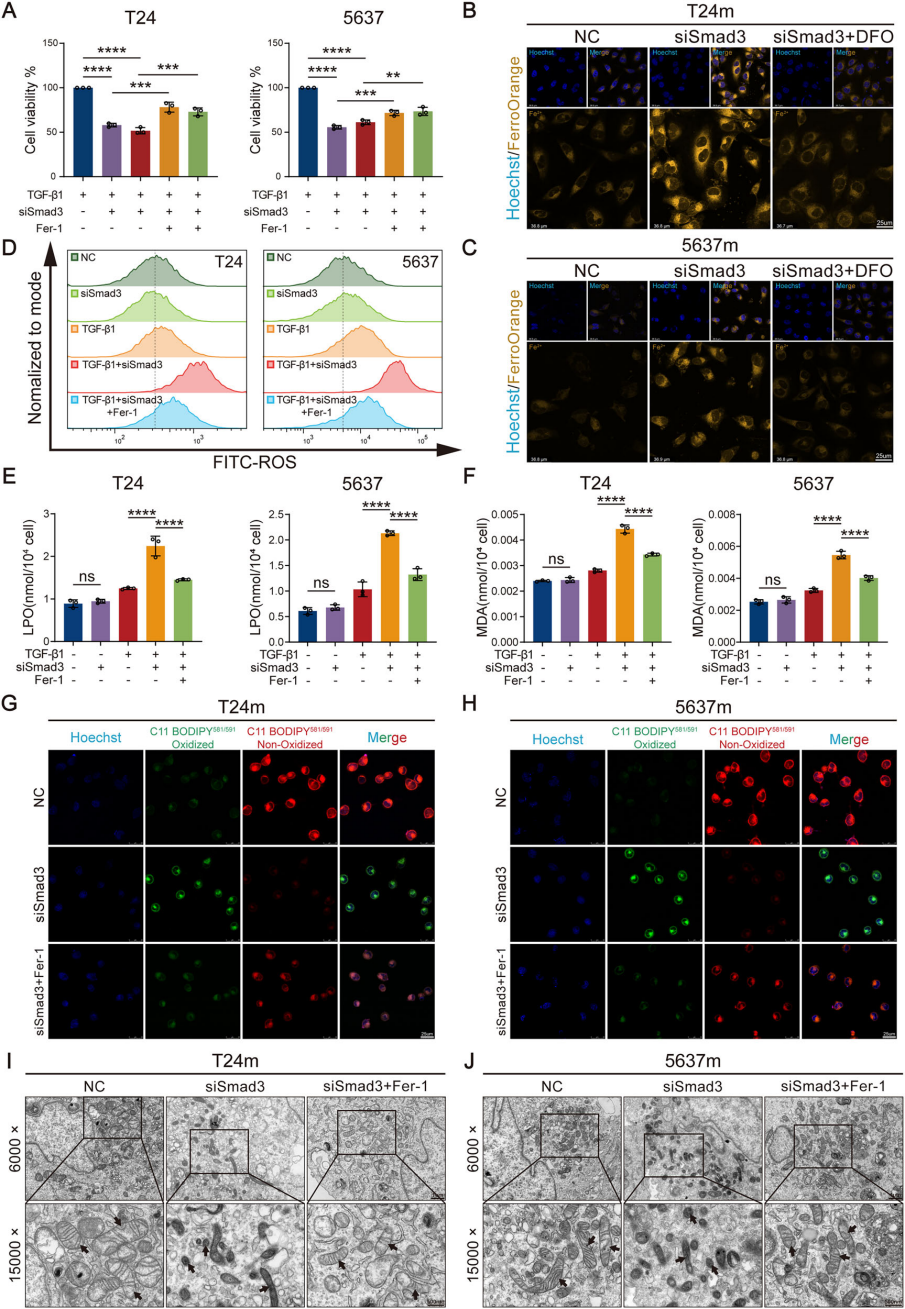
Smad3 knockdown induces ferroptosis in mesenchymal-like bladder cancer cells. **A** CCK-8 assay demonstrating the effect of Smad3 knockdown on proliferation. **B**, **C** Fe^2+^ levels in cells visualized by confocal imaging. **D** Flow cytometry analysis of total intracellular ROS levels after Smad3 knockdown. **E** LPO levels assessed via an LPO assay kit. **F** MDA content measured via an MDA assay kit. **G**, **H** Representative confocal images of lipid peroxidation. Red indicates non-oxidized lipids, and green indicates oxidized lipids. **I**, **J** Transmission electron micrographs revealed mitochondrial alterations following Smad3 knockdown (black arrows). Data are presented as the mean ± SEM. *p < 0.05, **p < 0.01, ***p < 0.001, ****p < 0.0001; ns, not significant (*p* > 0.05).

To clarify the regulatory role of Smad3 in ferroptosis of mesenchymal-like bladder cancer cells, we systematically validated its mechanism through multifaceted functional assays. CCK-8 cell viability assays demonstrated that Smad3 knockdown significantly reduced the viability of mesenchymal-like T24m/5637 m cells, whereas the ferroptosis inhibitor Fer-1—a small molecule compound that suppresses ferroptosis by scavenging lipid radicals and blocking iron-dependent lipid peroxidation—markedly reversed this effect (Fig. 5A), indicating that Smad3 deficiency-induced viability reduction was directly linked to ferroptosis execution. Further analysis of ferroptosis core markers revealed that Smad3 knockdown triggered a significant increase in intracellular labile Fe²⁺ levels, which was suppressed by DFO, an iron chelator that specifically inhibits ferroptosis through high-affinity binding to labile Fe²⁺, thereby blocking Fenton reaction-driven lipid peroxidation cascades (Fig. 5B–C). This confirms Fe²⁺ accumulation as a key trigger of ferroptosis upon Smad3 loss. Flow cytometry-based ROS detection showed substantially elevated ROS production in Smad3-knockdown groups, which was attenuated by Fer-1 co-treatment (Fig. 5D and S2C, D), suggesting that Smad3 deficiency drives ferroptosis via amplified oxidative stress. Biochemical analyses demonstrated that Smad3 knockdown caused significant depletion of reduced GSH and elevated levels of LPO and MDA, all of which were rescued by Fer-1 intervention (Figs. S2E–F and 5E–F). Consistently, C11-BODIPY fluorescence staining revealed intensified green fluorescence (lipid peroxidation products) and weakened red fluorescence (reduced lipids) in Smad3-knockdown cells (Fig. 5G, H), further confirming irreversible lipid peroxidation damage induced by Smad3 deficiency. We further employed TEM to analyze the mitochondrial ultrastructure in mesenchymal-like bladder cancer cells (T24m and 5637 m). The results revealed that Smad3 knockdown induced significant mitochondrial morphological changes compared to the control group (Fig. 5I, J). Specifically, we observed decreased mitochondrial volume, increased membrane electron density, and disrupted cristae structure—features that reproduce the hallmark ultrastructural alterations associated with ferroptosis [25, 26]. Moreover, these pathological changes were effectively rescued by Fer-1 treatment.

Notably, Smad3 knockdown in epithelial-like bladder cancer cells failed to induce significant alterations in total intracellular ROS levels or redox-related markers (GSH, LPO, and MDA) (Fig. 5D–F and S2C-F), in stark contrast to the dramatic changes observed in mesenchymal-like bladder cancer cells. This discrepancy corroborates that EMT-induced establishment of an “oxidative boost-reductive compensation” neo-homeostasis primes mesenchymal-like bladder cancer cells for heightened sensitivity to Smad3 deficiency, rendering them more vulnerable to ferroptosis upon disruption of redox equilibrium.

### Smad3 knockdown via ferroptosis suppresses bladder cancer cell migration, invasion, and proliferation

**Fig. 6:**
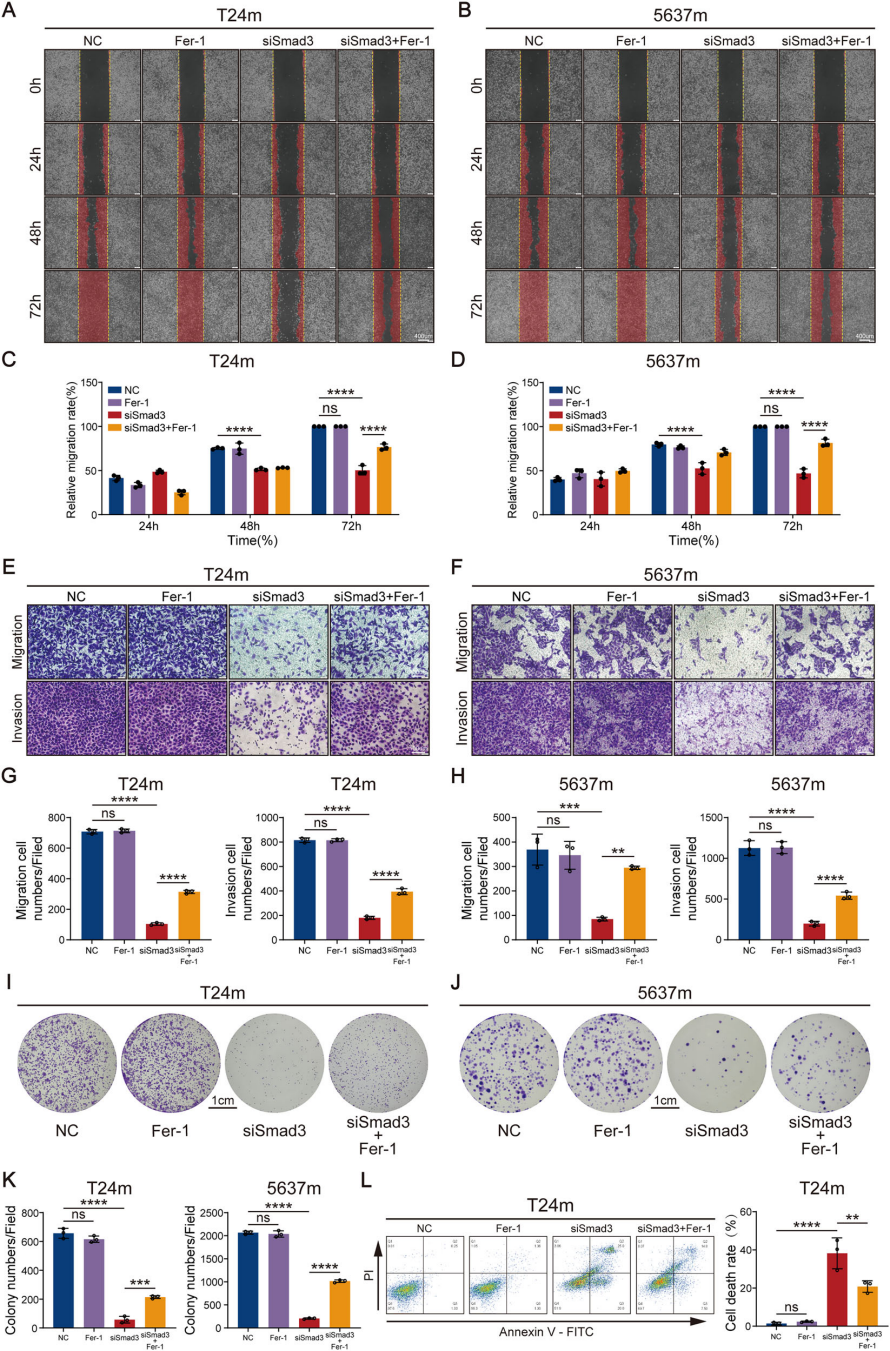
Smad3 promotes malignant phenotypes of bladder cancer cells in vitro. **A–D** The effect of Smad3 knockdown on cell migration was demonstrated by scratch healing assay. **E–H** The effect of Smad3 knockdown on migratory and invasive abilities was demonstrated by transwell migration and invasion assay. **I–K** The effect of Smad3 knockdown on clones formation abilities was demonstrated by colony formation assays. **L** Cell death analysis of Smad3-knockdown mesenchymal-like bladder cancer cells via flow cytometry. Data are presented as the mean ± SEM. *p < 0.05, **p < 0.01, ***p < 0.001, ****p < 0.0001; ns, not significant (*p* > 0.05).

Given that EMT confers mesenchymal traits through epithelial-mesenchymal molecular reprogramming, leading to loss of cell polarity, weakened adhesion, and enhanced migratory/invasive capacities, and based on our prior findings implicating Smad3 in regulating ferroptosis in mesenchymal-like bladder cancer cells, we further investigated Smad3’s functional role in post-EMT cellular behaviors. Wound healing assays demonstrated that Smad3 knockdown significantly inhibited the migratory capacity of mesenchymal-like bladder cancer cells, with partial restoration observed upon ferroptosis inhibitor Fer-1 co-treatment (Fig. 6A–D). Transwell migration and invasion assays further confirmed that Smad3 deficiency markedly attenuated both migratory and invasive abilities, which were partially rescued by Fer-1 (Fig. 6E–H). These results collectively demonstrate Smad3’s central regulatory role in sustaining EMT-driven migratory/invasive phenotypes, with partial dependence on ferroptosis. Colony formation assays revealed that Smad3 knockdown substantially suppressed cell proliferation and clonogenic potential (Fig. 6I–K), indicating its concurrent involvement in proliferation regulation. Flow cytometry analysis revealed significantly increased cell death in Smad3-knockdown mesenchymal-like bladder cancer cells (Fig. 6L).

### Multi-omics analysis identifies CISD2 as a direct downstream target of Smad3 in ferroptosis regulation

**Fig. 7:**
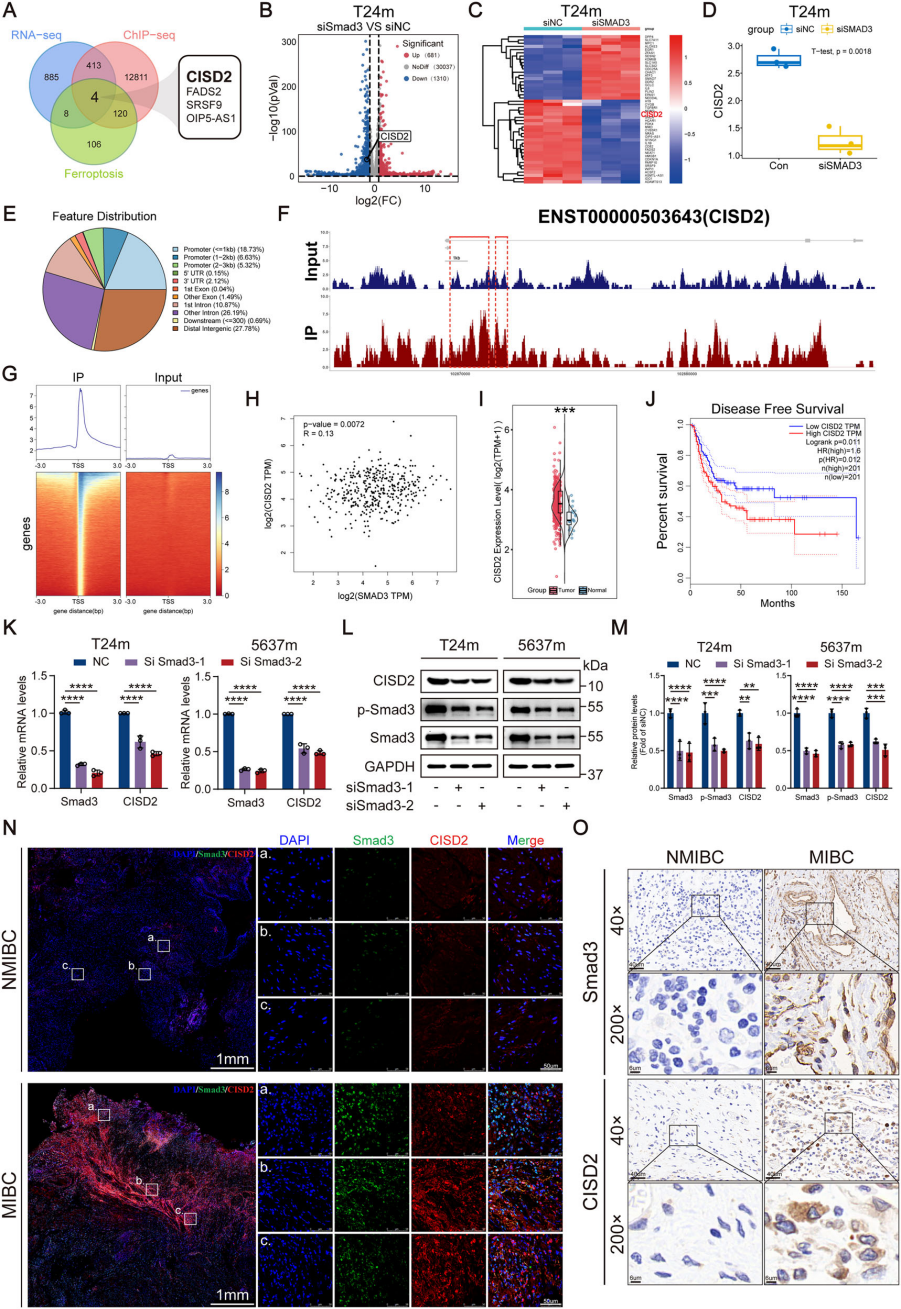
CISD2 was identified as a downstream target of Smad3 in regulating ferroptosis. **A** Venn diagram illustrating the overlapping genes identified from ChIP-seq, RNA-seq, and ferroptosis-related gene datasets. Volcano plot (**B**) and heatmap (**C**) demonstrating DEGs following Smad3 knockdown. **D** RNA-seq data analysis revealed CISD2 mRNA expression levels following Smad3 knockdown. **E** ChIP-seq analysis revealed the distribution of binding peaks across functional genomic elements. **F** Integrative Genomics Viewer (IGV) analysis of ChIP-seq data demonstrated Smad3 binding at the CISD2 locus. **G** ChIP-seq analysis reveals the depth distribution of p-Smad3 binding peaks across Gene Ontology categories and within 3 kb upstream/downstream regions of target genes. **H** A scatter plot shows the correlation between Smad3 and CISD2 expression levels. **I** Analysis of the TCGA database revealed differential expression of CISD2 between bladder cancer tissues and adjacent normal tissues. **J** Gene Expression Profiling Interactive Analysis (GEPIA) showed a significant correlation between CISD2 expression levels and disease-free survival (DFS) in patients. **K–M** The expression levels of Smad3 and CISD2 were analyzed by RT-qPCR and western blot. **N** Tissue IF revealed the expression levels of Smad3 and CISD2 in NMIBC and MIBC. **O** The expression levels of Smad3 and CISD2 in NMIBC and MIBC were assessed using IHC. Data are presented as the mean ± SEM. *p < 0.05, **p < 0.01, ***p < 0.001, ****p < 0.0001; ns, not significant (*p* > 0.05).

To elucidate the molecular mechanism by which Smad3 regulates ferroptosis in mesenchymal-like bladder cancer cells, we integrated RNA-seq, ChIP-seq, and functional validation to screen potential downstream targets (Fig. 7A). RNA-seq analysis of Smad3-knockdown versus control groups identified 1991 DEGs (681 upregulated, 1,310 downregulated), with CDGSH Iron-Sulfur Domain-containing protein 2(CISD2) showing significant downregulation in the knockdown group (Fig. 7B–D). The [2Fe-2S] cluster-containing iron-sulfur protein CISD2 plays a pivotal role in maintaining cellular proliferation and iron homeostasis [27]. Intersection analysis of these DEGs with Smad3 ChIP-seq-bound genes and ferroptosis-related genes narrowed the candidates to four target genes, including CISD2, a key iron-sulfur cluster biosynthesis regulator (Fig. 7A). ChIP-seq analysis revealed that 30.68% of p-Smad3 binding peaks were enriched near transcription start sites (TSS) of promoter regions (Fig. 7E), with a prominent binding signal detected at the CISD2 promoter (Fig. 7F), suggesting CISD2 as a direct transcriptional target of Smad3.

Clinical correlation analysis using the TCGA database revealed a positive correlation between Smad3 and CISD2 mRNA expression in BCa tissues (Fig. 7H). Notably, CISD2 expression was significantly elevated in tumor tissues compared to normal bladder tissues (Fig. 7I) and showed progressive upregulation with advancing clinical stage (Fig. S3B). IHC analysis from the Human Protein Atlas demonstrated significantly elevated CISD2 expression in bladder cancer tissues relative to normal bladder tissues (Fig. S3A). Prognostic analysis demonstrated that patients with high CISD2 expression had significantly shorter disease-free survival (Fig. 7J), highlighting CISD2’s functional importance in BCa progression. Functional validation experiments showed that Smad3 knockdown markedly suppressed CISD2 mRNA and protein levels via RT-qPCR and western blot (Fig. 7K–M). Multiplex IF staining revealed significantly higher Smad3 protein expression in MIBC tissues compared to NMIBC counterparts, accompanied by concurrent upregulation of its putative target CISD2 (Fig. 7N). Consistent results were obtained from IHC staining, further validating their co-expression pattern in clinical specimens (Fig. 7O). These data confirm CISD2 as a direct downstream target of Smad3, acting as a pivotal mediator in EMT-associated ferroptosis signaling.

### Smad3 regulates ferroptosis in mesenchymal-like bladder cancer cells through CISD2

**Fig. 8:**
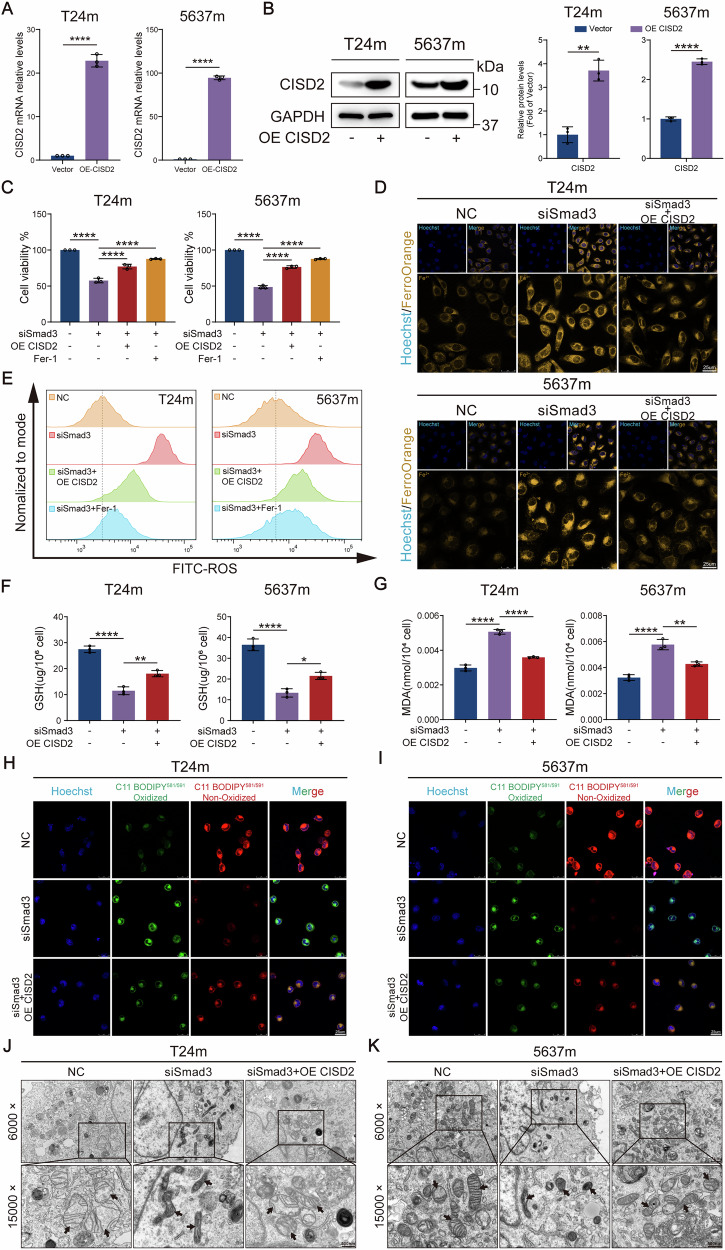
CISD2 rescues Smad3 knockdown-induced ferroptosis phenotypic alterations. **A**, **B** RT-qPCR and western blot analyses confirming the transfection efficiency of CISD2 plasmid overexpression. **C** CCK-8 assays revealed cell proliferation in different groups. **D** Fe^2+^ levels in cells visualized by confocal imaging. **E** Flow cytometry analysis of total intracellular ROS levels after CISD2 overexpression. **F** GSH content measured via a GSH assay kit. **G** MDA content measured via an MDA assay kit. **H**, **I** Representative confocal images of lipid peroxidation. Red indicates non-oxidized lipids, and green indicates oxidized lipids. **J**, **K** Transmission electron micrographs revealed mitochondrial alterations (black arrows). Data are presented as the mean ± SEM. *p < 0.05, **p < 0.01, ***p < 0.001, ****p < 0.0001; ns, not significant (*p* > 0.05).

To validate the critical role of CISD2 as a downstream effector of Smad3 in ferroptosis regulation, we performed CISD2 overexpression rescue experiments in Smad3-knockdown mesenchymal-like bladder cancer cells. RT-qPCR and western blot confirmed efficient CISD2 overexpression in the cellular models (Fig. 8A, B). Functional assays showed that CISD2 overexpression significantly rescued the Smad3 knockdown-induced reduction in cell viability, as evidenced by CCK-8 assays (Fig. 8C), indicating that CISD2 restoration counteracts the viability suppression caused by Smad3 deficiency.

Further analysis of ferroptosis core markers demonstrated that CISD2 overexpression effectively reduced intracellular labile Fe²⁺ levels (Fig. 8D), attenuated ROS generation (Fig. 8E and Fig. S3C, D), elevated reduced GSH content (Fig. 8F), and significantly inhibited Smad3 knockdown-induced accumulation of LPO and MDA (Figs. S3E-F and 8G). C11-BODIPY fluorescence staining visually demonstrated diminished green fluorescence (lipid peroxidation products) and enhanced red fluorescence (reduced lipids) in CISD2-overexpressing cells compared to Smad3-knockdown counterparts (Fig. 8H, I), aligning with biochemical measurements. TEM analysis further demonstrated that CISD2 overexpression rescued the mitochondrial alterations induced by Smad3 knockdown (Fig. 8J, K), providing morphological evidence supporting these findings. These results collectively confirm across multiple dimensions—cell viability, iron homeostasis, oxidative stress, and lipid peroxidation—that CISD2 overexpression specifically rescues Smad3 deficiency-driven ferroptosis, establishing CISD2 as the key downstream effector of Smad3 with a core regulatory role in EMT-associated ferroptosis pathways.

### Targeting the Smad3/CISD2 axis induces ferroptosis in mesenchymal-like bladder cancer cells in vivo

**Fig. 9:**
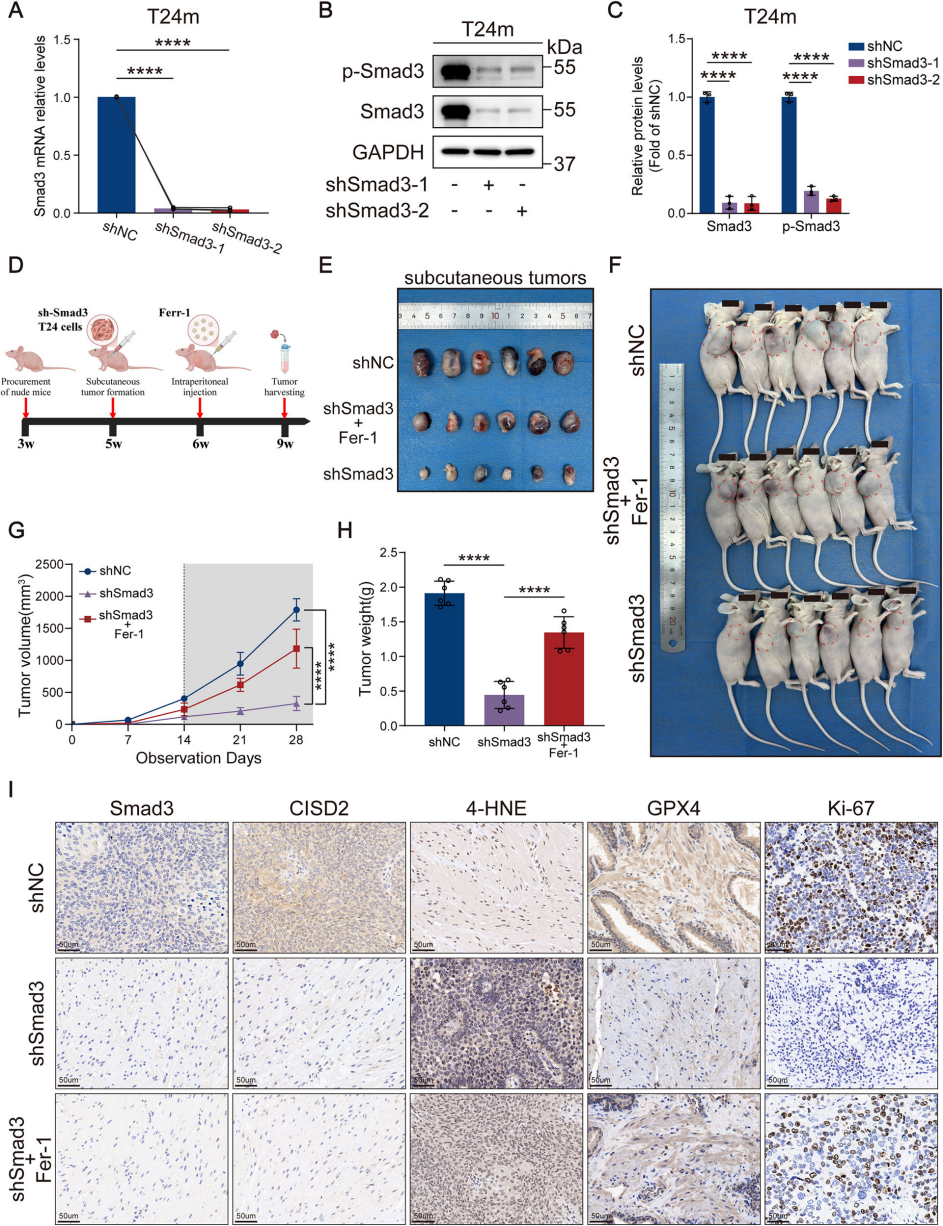
Targeting the Smad3/CISD2 axis inhibits bladder cancer growth in vivo by inducing ferroptosis. **A–C** RT-qPCR and western blot analyses validating the stable silencing efficiency of Smad3. **D** Schematic diagram of the experimental design for the animal model. **E** Representative images of subcutaneous tumors. **F** Photographs of xenograft tumors in nude mice. **G** Tumor growth curves in the xenograft model. **H** Comparison of subcutaneous tumor weights. **I** IHC staining of Smad3, CISD2, 4-HNE, GPX4, and Ki-67 in xenograft tumors. Data are presented as the mean ± SEM. *p < 0.05, **p < 0.01, ***p < 0.001, ****p < 0.0001; ns, not significant (*p* > 0.05).

To validate the Smad3/CISD2 axis-driven EMT-associated ferroptosis mechanism identified in vitro, we established a nude mouse subcutaneous xenograft model to systematically assess the effects of Smad3 targeting on tumor growth and its ferroptosis dependency. Mesenchymal-like bladder cancer cell lines with stable Smad3 knockdown were generated via lentiviral infection, with knockdown efficiency confirmed by RT-qPCR and western blot (Fig. 9A–C). Three experimental groups were designed: control (shNC), Smad3 knockdown (shSmad3), and Smad3 knockdown combined with ferroptosis inhibitor treatment (shSmad3 + Fer-1). Cells from each group were subcutaneously inoculated into nude mice, followed by intraperitoneal injections of Fer-1 for the shSmad3 + Fer-1 group or equivalent saline for other groups post-tumor formation (Fig. 9D). Serial tumor growth monitoring revealed significantly smaller tumor volumes in the shSmad3 group compared to controls (Fig. 9G). Endpoint analysis demonstrated markedly reduced tumor weights and volumes in the shSmad3 group (Fig. 9E, F, H). Fer-1 treatment partially reversed Smad3 knockdown-induced tumor growth suppression, confirming that Smad3 deficiency inhibits tumor growth via a ferroptosis-dependent pathway.

IHC staining results demonstrated that, compared to the control group, the expression of the key ferroptosis-inhibitory protein GPX4 was significantly decreased in shSmad3 tumor tissues, while the expression of the lipid peroxidation marker 4-hydroxynonenal (4-HNE, a highly reactive aldehyde generated from membrane lipid peroxidation that reflects intracellular oxidative stress and serves as a specific biomarker for ferroptosis and oxidative damage-related pathologies) was markedly increased. These findings indicate that the cellular antioxidant defense system is impaired, leading to a disruption of redox homeostasis and thereby inducing ferroptosis. Additionally, a reduction in the positive rate of the proliferation marker Ki-67 was observed. Treatment with Fer-1 reversed these alterations (Fig. 9I). Mechanistically, IHC analysis revealed markedly reduced CISD2 protein levels in shSmad3 tumors (Fig. 9I), consistent with in vitro findings, thereby reaffirming CISD2 as a downstream target of Smad3. Collectively, these in vivo data demonstrate that Smad3 knockdown inhibits BCa growth and progression by suppressing CISD2-mediated ferroptosis, providing functional evidence for targeting the Smad3-CISD2 axis to intervene in tumor malignant transformation.

## Discussion

BCa mortality is strongly associated with metastatic progression, with approximately 25% of patients presenting with MIBC at diagnosis, more than half of whom will eventually develop metastatic disease [28, 29]. To address this clinical challenge, recent studies have employed multidimensional therapeutic strategies to inhibit BCa and metastasis. At the molecular level, targeted inhibition of the SLC2A11-MIF/PTBP1-mediated mRNA stability regulatory pathway blocks bladder cancer cell proliferation, whereas modulation of the CircPTK2/PABPC1/SETDB1 signaling axis suppresses EMT, thereby inhibiting BCa metastasis [30, 31]. In the field of drug delivery, the Fe-EGCG@RSL3 nanomedicine and oxaliplatin prodrug liposomes significantly enhance the therapeutic efficacy against BCa through dual mechanisms of ferroptosis-immunity synergy and chemo-immunotherapy synergy, respectively [32, 33]. In the realm of immunomodulation, CRISPR-engineered outer membrane vesicles potentiate antitumor immunity through T cell activation, whereas mitochondria-targeted brequinar liposomes enhance immunogenicity by inducing ferroptosis—these dual approaches provide innovative strategies to overcome immunotherapy resistance in BCa [34, 35]. Based on this research background, our study systematically elucidated the regulatory role of ferroptosis-related signaling pathways in BCa invasion and metastasis. We have for the first time discovered that the Smad3/CISD2 axis significantly influences ferroptosis susceptibility in mesenchymal-like bladder cancer cells by remodeling redox homeostasis. This pivotal finding reveals the molecular basis of Smad3/CISD2-mediated ferroptosis regulation, providing an innovative targeted intervention strategy for the clinical treatment of MIBC.

**Fig. 10:**
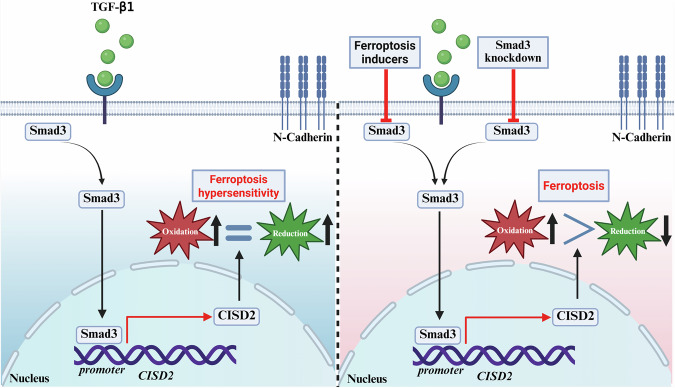
Mechanism diagram: Mesenchymal-like bladder cancer cells are more sensitive to ferroptosis (left) and Smad3/CISD2 axis regulate ferroptosis in mesenchymal-like bladder cancer cells (right).

EMT, as a core regulatory mechanism of tumor invasion and metastasis, has been extensively studied in terms of its molecular regulatory network. Current research indicates that multiple signaling pathways (e.g., TGF-β, Wnt/β-catenin) and epigenetic modifications can regulate the EMT process. Notably, numerous studies have confirmed that core transcription factors such as Zinc Finger E-Box Binding Homeobox 1 (ZEB1), Twist, and Snail directly modulate EMT by suppressing E-cadherin expression and activating mesenchymal markers (e.g., N-cadherin, Vimentin) [36, 37]. In addition, the role of post-translational modifications in EMT regulation has become increasingly prominent in recent years. The latest research reveals that UBE2S interacts with TRIM21 to mediate LPP degradation via the formation of K11-linked ubiquitination, thereby promoting EMT and breast cancer lymphatic metastasis [38]. Similarly, MUL1-mediated SUMOylation of HSPA9 can inhibit EMT and cancer cell migration [39]. However, despite significant progress in understanding the regulatory mechanisms of EMT, the intricate interplay between EMT and ferroptosis susceptibility—particularly the regulatory role of EMT-induced metabolic reprogramming in ferroptosis—remains incompletely elucidated. Although previous studies have revealed the dual characteristics of EMT in the regulation of ferroptosis sensitivity [20, 23]. It can not only promote the enhancement of oxidability by upregulating polyunsaturated fatty acids (PUFA) and other ways, but also activate the antioxidant pathway to form a defensive barrier [20]. However, the molecular mechanisms of the remodeling of the redox balance related to EMT and their biological effects under different TMEs still need to be further explored. In this study, we elucidate for the first time in BCa that TGF-β/Smad3-mediated EMT dynamically remodels redox homeostasis through CISD2 upregulation: Smad3 directly transcriptionally activates CISD2 by binding to conserved Smad-binding elements (SBEs) in its promoter region. The induced CISD2 maintains mitochondrial iron homeostasis, concurrently elevating baseline oxidative levels during EMT and activating compensatory antioxidant responses, ultimately sensitizing mesenchymal-like bladder cancer cells to ferroptosis [10, 40]. This discovery innovatively proposes an “oxidation-antioxidation biphasic equilibrium” model, wherein EMT-transformed cells establish antioxidant reserves through CISD2-mediated iron metabolic regulation while operating at heightened oxidative baselines, positioning tumor cells at a redox critical threshold (Fig. 10). Integrated multi-omics analyses identify the Smad3/CISD2 signaling axis as the key molecular hub connecting EMT and ferroptosis regulation. The bidirectional regulatory properties of this axis not only provide novel insights into tumor heterogeneity but also suggest that targeting this axis may disrupt redox equilibrium to induce ferroptosis specifically in MIBC, offering translational significance for developing therapeutic strategies against invasion-metastasis-associated treatment resistance.

This study reveals a novel mechanism by which EMT enhances ferroptosis susceptibility through remodeling the redox dynamic equilibrium in BCa cells. Building on the redox dynamic equilibrium theory proposed by Professor Helmut Sies, we demonstrate that EMT in BCa cells is not only characterized by downregulation of epithelial markers (E-cadherin) and upregulation of mesenchymal markers (N-cadherin, Vimentin) (Fig. 2A, B), but more importantly involves a profound reconstruction of redox homeostasis [41]. On one hand, oxidative markers, such as LPO (Fig. 2J) accumulate significantly, elevating overall cellular oxidative stress. On the other hand, the upregulation of key antioxidant enzymes (e.g., GPX4 and SLC7A11, Fig. 2I) and increased reduced GSH levels (Fig. 2K) establish a new redox equilibrium (Fig. 2L). This aligns with the theory proposed by Guo et al., suggesting that EMT cells counteract oxidative stress via three pathways—antioxidant defense, lipid remodeling, and iron metabolism reprogramming [42]. Notably, this dynamic equilibrium positions cells in a more fragile critical state. Prior studies posited that EMT-induced E-cadherin loss suppresses Neurofibromatosis Type 2(NF2) (also known as merlin) and Hippo signaling to promote ferroptosis sensitivity [43]. Schwab et al. further revealed that the EMT core transcription factor Zeb1 drives PUFA membrane incorporation—enhancing migratory capacity while increasing ferroptosis risk due to oxidation-prone PUFA enrichment [20]. Consistent with our experimental data, EMT cells with remodeled redox homeostasis exhibit heightened vulnerability to ferroptosis inducer Erastin (Fig. 3A), validating the intrinsic link between redox remodeling and ferroptosis susceptibility (Fig. 3B–I). These findings provide fresh insights into the connection between EMT-mediated metastasis and ferroptosis sensitivity.

The highly heterogeneous biological characteristics of EMT confer remarkable diversity in its regulatory mechanisms over ferroptosis susceptibility. In the tumor microenvironment, complex signals including Wnt pathway, Notch pathway, growth factor receptors, inflammatory cytokines, and hypoxia can trigger EMT, which exhibits substantial heterogeneity across different tumors or even within the same tumor mass due to microenvironmental complexity [44, 45]. For example, the TGF-β signal gradient can induce the spatial patterned distribution of EMT in breast cancer [46]. This not only drives tumor cells to exhibit a continuous spectrum of EMT states but also forms a heterogeneous pattern of ferroptosis sensitivity through the differential reconstruction of the redox regulation network. Notably, we observed TGF-β1 concentration-dependent changes in ferroptosis-related markers (e.g., LPO and GSH, Fig. 2J, K) during the epithelial-to-mesenchymal transition of BCa cells, confirming the heterogeneous EMT-ferroptosis regulatory axis. Mechanistically, this heterogeneity stems from tumor-type-specific activation of downstream EMT effectors: Zeb1-driven PUFA membrane remodeling in breast cancer [20], E-cadherin loss-mediated NF2/Hippo pathway suppression in colorectal cancer [43], and Smad3 knockout-induced redox homeostasis disruption in BCa (this study) collectively form a “multi-mechanism network” underlying EMT-regulated ferroptosis. Intriguingly, these mechanisms converge at core redox nodes such as LPO accumulation (Fig. 2J), yet cells dynamically adapt their responses via molecular switches (e.g., Smad3, Zeb1, NF2) in response to microenvironmental cues. Such mechanistic diversity reflects the complexity of EMT regulatory networks during tumor evolution and provides a theoretical foundation for developing precision ferroptosis therapies tailored to tumor types and microenvironmental features.

This study establishes Smad3 as the central hub of TGF-β/Smad signaling in mediating EMT and ferroptosis in BCa. In the canonical TGF-β1 signaling cascade, ligand-receptor binding triggers Smad3 phosphorylation and nuclear translocation (Fig. 4F, G), which serves not only as a critical transcriptional node driving EMT-associated gene expression but also reprograms redox homeostasis to confer ferroptosis susceptibility [47**–**50]. We observed synchronized nuclear enrichment of p-Smad3 with EMT progression in BCa cells. Specific Smad3 knockdown not only blocked EMT phenotypic transition but also markedly altered ferroptosis markers (MDA, GSH, LPO, and ROS; Fig. 5D–H and S2C–F), demonstrating its dual functionality in coordinating EMT and ferroptosis regulation. Mechanistically, nuclear Smad3 orchestrates the expression of both EMT effectors (e.g., E-cadherin, Vimentin) and redox homeostasis regulators (e.g., GPX4, xCT) through its transcriptional network. Smad3 deficiency disrupts the balance between antioxidant defense systems and lipid peroxidation pressure (Fig. 5E–H and S2E, F). This finding complements mechanisms observed in other cancers, such as Zeb1-mediated membrane phospholipid remodeling in breast cancer and the E-cadherin-NF2/Hippo axis in colorectal cancer [20, 43]. Collectively, these insights identify the TGF-β/Smad3 axis as a novel molecular target for developing precision therapies that simultaneously inhibit EMT and activate ferroptosis.

This study integrates Smad3-ChIP sequencing with multi-omics analyses to identify CISD2 (encoding NAF-1) as the core effector of Smad3 in ferroptosis regulation, establishing the TGF-β/Smad3-CISD2 axis as a molecular bridge linking EMT and ferroptosis. As a member of the iron–sulfur protein family, NAF-1 (encoded by CISD2) plays a pivotal role in maintaining oxidative stress resistance in tumor cells through mitochondrial 2Fe-2S cluster transfer and iron homeostasis regulation [51, 52]. We demonstrate that Smad3 directly binds to the CISD2 promoter (Fig. 7F), driving its transcriptional expression to suppress ferroptosis. In mesenchymal-like bladder cancer cells, this mechanism manifests as follows: sustained Smad3 activation during EMT upregulates CISD2 to maintain iron metabolic equilibrium, while Smad3 knockdown suppresses CISD2 expression, leading to Fe²⁺ overload (Fig. 8D), explosive accumulation of ferroptosis markers such as LPO and MDA (Fig. 8G and S3E-F), concurrent with GSH depletion (Fig. 8F) and total intracellular ROS levels surge (Fig. 8E), ultimately disrupting post-EMT redox balance to induce ferroptosis. The functional conservation of CISD2 is corroborated across multiple cancers: CISD2 expression correlates significantly with prognosis in head and neck, liver, and gastric cancers [52**–**54]. Strikingly, CISD2 overexpression in mesenchymal-like bladder cancer cells fully reverses ferroptotic phenotypes caused by Smad3 deficiency (Fig. 8D–K). This discovery not only expands the classical transcriptional regulatory role of Smad3 into the ferroptosis field but also reveals that CISD2 reduces ferroptosis during EMT by maintaining iron homeostasis in mesenchymal-phenotype cells. These findings provide new directions for developing synergistic therapeutic strategies targeting both EMT and ferroptosis via the TGF-β/Smad3-CISD2 signaling axis.

While this study elucidates the critical role of the Smad3-CISD2 axis in EMT-mediated ferroptosis regulation via TGF-β1-induced bladder cancer cell models, several limitations warrant acknowledgment. First, the current experimental system relies on exogenous cytokine-induced EMT, which differs from spontaneous EMT in the tumor microenvironment (e.g., cross-activation of multiple signaling pathways, cell-cell/cell-matrix interactions). Future studies should integrate organoid models and genetically engineered mouse models (GEMMs) with controlled EMT activation to better mimic the complexity of in vivo EMT and validate core findings. Second, although CISD2 is identified as a key downstream target of Smad3, how it interacts with multi-layered networks of iron metabolism (e.g., iron transport/storage) and redox homeostasis (e.g., GSH-GPX4 axis, ROS regulation) remains unclear. Systematic exploration using phosphoproteomics, metabolic flux analysis, and other advanced technologies is required to dissect the crosstalk between Smad3 and EMT-related pathways like Wnt/β-catenin and HIF-1α. Third, the proposed “oxidation-antioxidation neo-homeostasis” lacks real-time dynamic monitoring of its heterogeneous distribution at the single-cell level (e.g., redox state fluctuations across EMT transition states). Addressing this gap will necessitate single-cell RNA sequencing and intravital fluorescence imaging to spatiotemporally resolve redox remodeling heterogeneity during EMT progression.

## Conclusion

Through multi-omics analyses and functional validation, this study elucidates for the first time that Smad3, a core EMT regulator in BCa cells, not only drives EMT but also transcriptionally activates CISD2 to mediate mitochondrial iron homeostasis and antioxidant defense, establishing an “oxidation-augmentation and antioxidation-compensation” dynamic equilibrium that ultimately enhances cellular susceptibility to ferroptosis (Fig. 10). Further mechanistic investigations reveal the Smad3-CISD2 axis as a specific molecular bridge linking EMT to ferroptosis regulation in BCa—Smad3 converts EMT-induced oxidative stress into altered ferroptosis sensitivity by modulating CISD2 expression. Future studies should focus on developing targeted strategies against the Smad3-CISD2 axis for personalized treatment of MIBC, offering novel translational insights for intervening in tumor invasion, metastasis, and malignant progression.

## Supplementary information

Supplementary Materials

Appendix Table S1-3

original data

original data

Reproducibility Checklist

## Data Availability

The datasets supporting the findings of this study are available from the corresponding author upon reasonable request.
