# Supplementary material for: Targeting the SMAD3/CISD2 axis suppresses bladder cancer progression by promoting ferroptosis in mesenchymal-like bladder cancer cells
Source: Cell Death Dis. 2025 Dec 18;17(1):101. doi: 10.1038/s41419-025-08339-9 (PMC12847906; doi:10.1038/s41419-025-08339-9)
Supplement: Supplementary file 1 — Supplementary Materials [file 41419_2025_8339_MOESM1_ESM.docx]

# **Supplementary Materials**

**Targeting the SMAD3/CISD2 axis suppresses bladder cancer progression by promoting ferroptosis in mesenchymal-like bladder cancer cells**

Yulin Sun ^1^, Jianghui Wang ^3^, Da Gu ^2^, Huanmin Lou ^2^, Jinpeng Sun ^1^, Zilian Cui ^1*^, Weiting Kang ^1*^

^1^Department of Urology, Shandong Provincial Hospital Affiliated to Shandong First Medical University, Jinan, Shandong 250021, China

^2^Department of Plastic Surgery, Central Hospital Affiliated to Shandong First Medical University, Jinan, Shandong 250013, China

^3^Department of Orthopedic Surgery, The Second People's Hospital of Dongying, Dongying, Shandong 257000, China

^*^Correspondence to: Weiting Kang, email: [kangweiting@sdfmu.edu.cn](mailto:kangweiting@sdfmu.edu.cn); Zilian Cui, email: [122526195@qq.com](mailto:122526195@qq.com).

**Table of Contents**

**Supplementary Figure legends**

**Fig. S1**The construction and validation of mesenchymal-like bladder cancer cell model

**Fig. S2**Smad3 modulates ferroptosis in mesenchymal-like bladder cancer cells

**Fig. S3**CISD2 is upregulated in bladder tumors and rescues Smad3 knockdown-induced ferroptosis

**Supplementary Tables**

**Appendix Table S1** Primers used for qRT-PCR

**Appendix Table S2** Sequences of siRNAs and shRNAs used in this study

**Appendix Table S3** Clinical dataset for survival analysis of TCGA bladder cancer patients


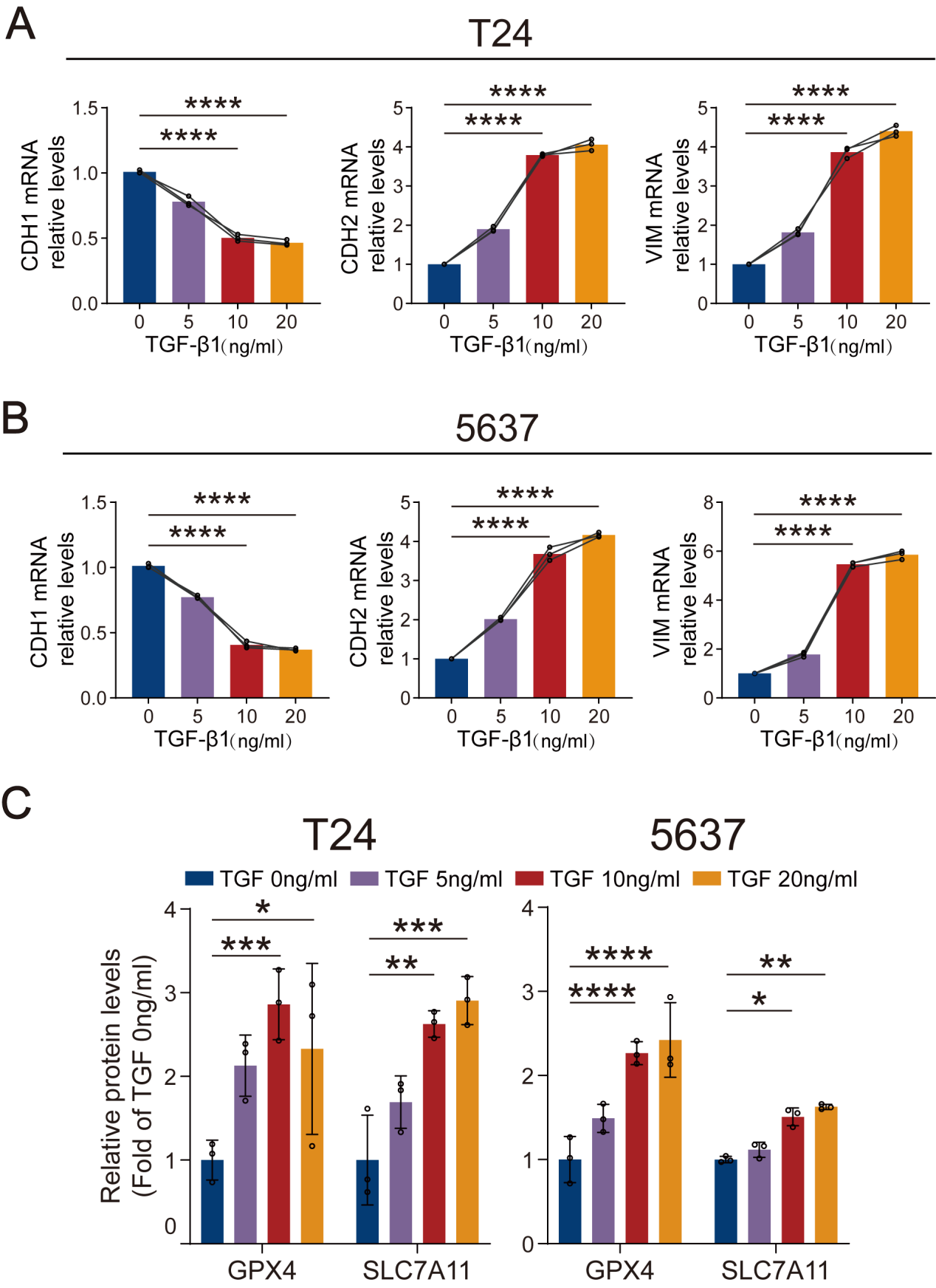


**Fig. S1 The construction and validation of mesenchymal-like bladder cancer cell model**

**A-B** RT-qPCR analysis showing TGF-β1-mediated regulation of EMT marker mRNA levels (CDH1, VIM, CDH2). **C** Western blot confirming TGF-β1-induced suppression of epithelial markers (E-cadherin) and upregulation of mesenchymal markers (Vimentin, N-cadherin). Relative protein levels of each protein were normalized to GAPDH. Data are presented as the mean ± SEM. *p < 0.05, **p < 0.01, ***p < 0.001, ****p < 0.0001; ns, not significant (p > 0.05).


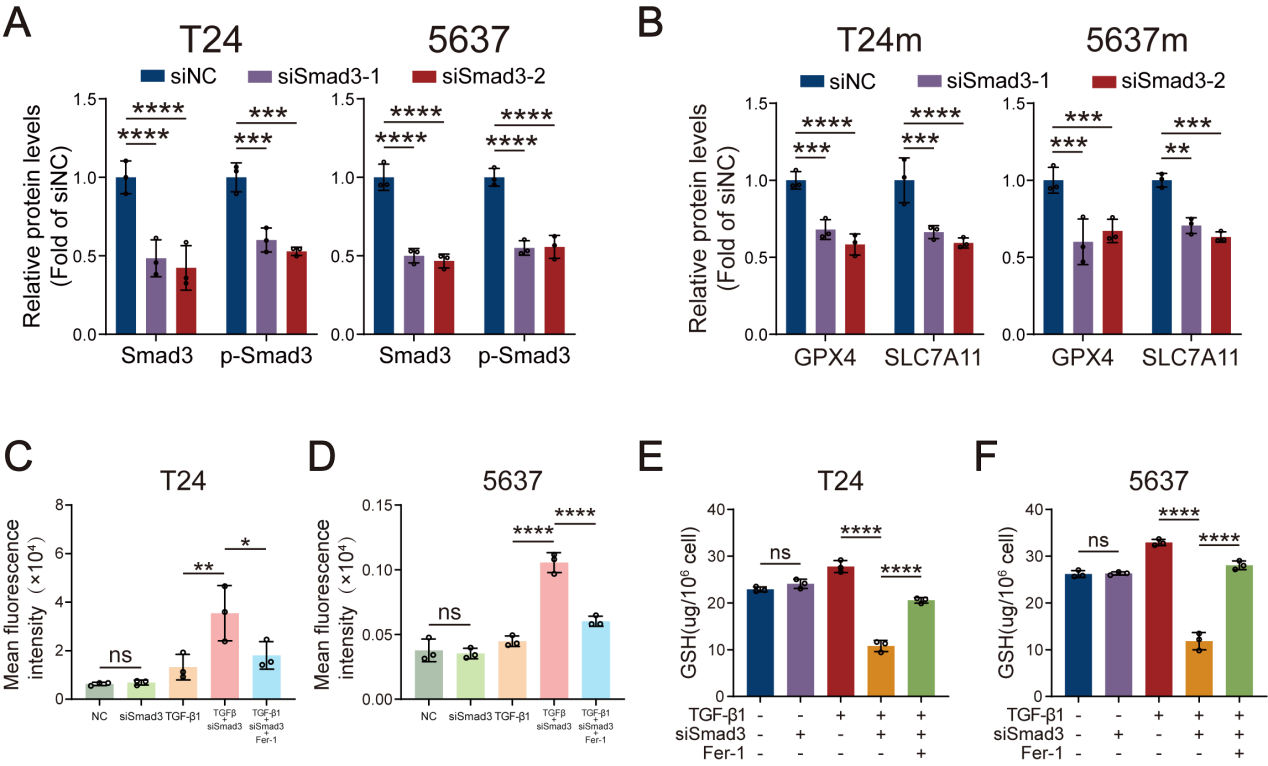


**Fig. S2 Smad3 modulates ferroptosis in mesenchymal-like bladder cancer cells**

**A** Western blot analysis for validation of Smad3 knockdown in T24m and 5637m cells. **B** Western blot analysis of ferroptosis suppressor proteins (such as GPX4 and SLC7A11) in T24m and 5637m cells following Smad3 knockdown. Relative protein levels of each protein were normalized to GAPDH. **C-D** Flow cytometry analysis of total intracellular ROS levels after Smad3 knockdown. **E-F** GSH levels measured using a GSH assay kit after Smad3 knockdown. Data are presented as the mean ± SEM. *p < 0.05, **p < 0.01, ***p < 0.001, ****p < 0.0001; ns, not significant (p > 0.05).


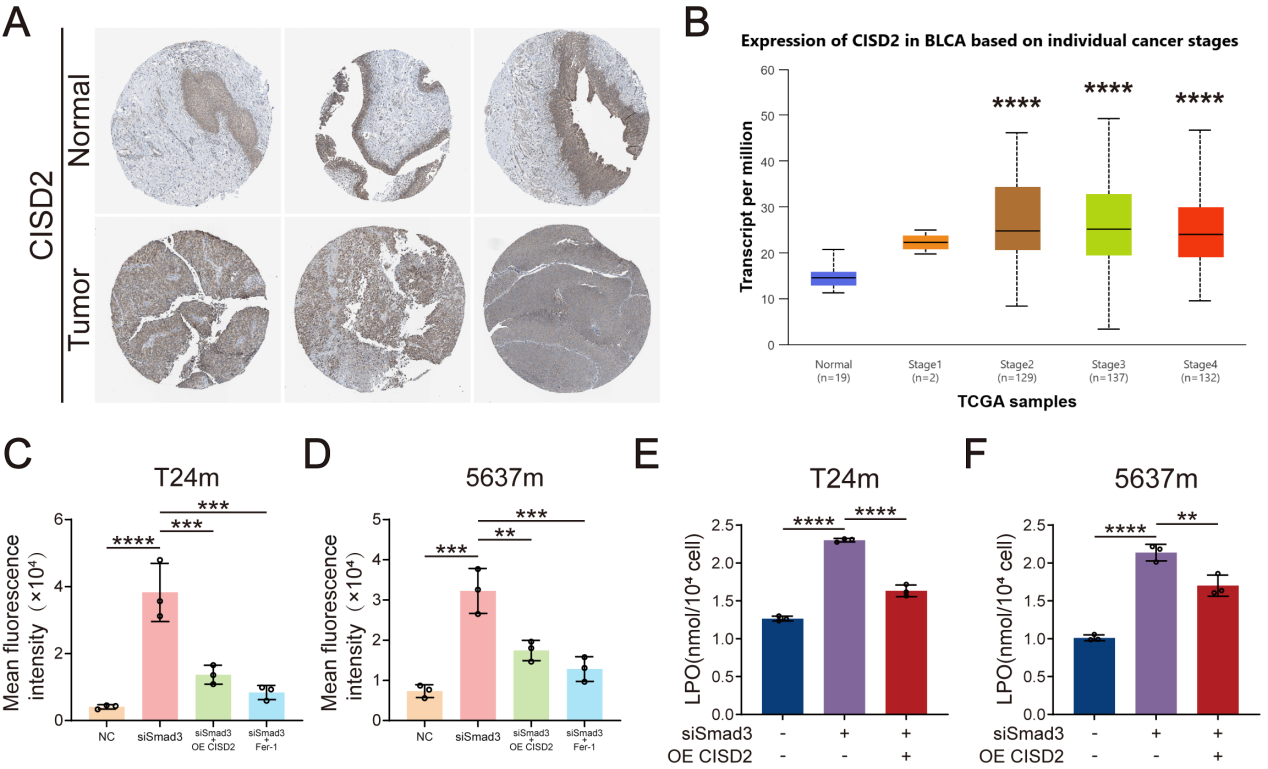


**Fig. S3 CISD2 is upregulated in bladder tumors and rescues Smad3 knockdown-induced ferroptosis**

**A** The IHC images of CISD2 in normal bladder tissues and bladder cancer tissues extracted from the Human Protein Atlas. **B** Analysis of the TCGA database demonstrated differential expression of CISD2 across distinct clinical stages in BCA. **C-D** Flow cytometry analysis of total intracellular ROS levels after CISD2 overexpression. **E-F** LPO levels were measured via an LPO assay kit. Data are presented as the mean ± SEM. *p < 0.05, **p < 0.01, ***p < 0.001, ****p < 0.0001; ns, not significant (p > 0.05).

**Appendix Table S1** Primers used for qRT-PCR

| Primers  for qRT-PCR | Sequence, 5' to 3' |
| --- | --- |
| GAPDH-F | GTCTCCTCTGACTTCAACAGCG |
| GAPDH-R | ACCACCCTGTTGCTGTAGCCAA |
| E-cadherin-F | GCCTCCTGAAAAGAGAGTGGAAG |
| E-cadherin-R | TGGCAGTGTCTCTCCAAATCCG |
| N-cadherin-F | CCTCCAGAGTTTACTGCCATGAC |
| N-cadherin-R | GTAGGATCTCCGCCACTGATTC |
| Vimentin-F | AGGCAAAGCAGGAGTCCACTGA |
| Vimentin-R | ATCTGGCGTTCCAGGGACTCAT |
| GPX4-F | ACAAGAACGGCTGCGTGGTGAA |
| GPX4-R | GCCACACACTTGTGGAGCTAGA |
| SLC7A11-F | TCCTGCTTTGGCTCCATGAACG |
| SLC7A11-R | AGAGGAGTGTGCTTGCGGACAT |
| FTH1-F | TGAAGCTGCAGAACCAACGAGG |
| FTH1-R | GCACACTCCATTGCATTCAGCC |
| FACL4-F | GCTATCTCCTCAGACACACCGA |
| FACL4-R | AGGTGCTCCAACTCTGCCAGTA |
| Smad3-F | TGAGGCTGTCTACCAGTTGACC |
| Smad3-R | GTGAGGACCTTGTCAAGCCACT |
| CISD2-F | CCAGTCCCTGAAAGCATTACCG |
| CISD2-R | CTTCTTCGGGAGGAATGGACGA |

F, forward; R, reverse.

**Appendix Table S2** Sequences of siRNAs and shRNAs used in this study

| siRNAs | Sequence, 5' to 3' |
| --- | --- |
| si-NC-S | UUCUCCGAACGUGUCACGUTT |
| si-NC-A | ACGUGACACGUUCGGAGAATT |
| si-Smad3-1-S | GCUGUUCCAGUGUGUCUUAT |
| si-Smad3-1-A | UAAGACACACUGGAACAGCT |
| si-Smad3-2-S | GGUGCUCCAUCUCCUACUAT |
| si-Smad3-2-A | UAGUAGGAGAUGGAGCACCT |

siRNAs: small interfering RNAs; S: sense strand; A: antisense strand.

| shRNAs | Sequence, 5' to 3' |
| --- | --- |
| sh-NC | GCGTGATCTTCACCGACAAGATTCAAGAGATCTTGTCGGTGAAGATCACGCTTTTTT |
| sh-Smad3-1 | GGTGCTCCATCTCCTACTACGTTCAAGAGACGTAGTAGGAGATGGAGCACCTTTTTT |
| sh-Smad3-2 | GGATTGAGCTGCACCTGAATGTTCAAGAGACATTCAGGTGCAGCTCAATCCTTTTTT |

shRNAs: short-hairpin RNAs.

**Appendix Table S3** Clinical dataset for survival analysis of TCGA bladder cancer patients

| TCGA_id | fustat | futime | gender | age | M | N | T | Stage |
| --- | --- | --- | --- | --- | --- | --- | --- | --- |
| TCGA-HQ-A5ND | 1 | 0.751 | male | 78.233 | M0 | N1 | T3b | Stage IV |
| TCGA-FD-A6TC | 0 | 0.512 | female | 79.351 | MX | N0 | T4a | Stage III |
| TCGA-DK-AA74 | 0 | 4.679 | male | 75.973 | M0 | N0 | T3 | Stage III |
| TCGA-GV-A3JV | 1 | 1.189 | male | 66.419 | MX | N1 | T3b | Stage IV |
| TCGA-XF-A8HH | 1 | 0.156 | female | 61.351 | M0 | N2 | T3b | Stage IV |
| TCGA-DK-A2I1 | 0 | 1.496 | female | 73.222 | M0 | N0 | T2b | Stage II |
| TCGA-XF-A9SK | 1 | 1.332 | male | 65.805 | MX | N1 | T3b | Stage IV |
| TCGA-ZF-AA5H | 0 | 2.458 | female | 60.608 | M0 | N2 | T3b | Stage IV |
| TCGA-E7-A7PW | 0 | 1.14 | male | 63.17 | M0 | NX | T3a | Stage III |
| TCGA-PQ-A6FI | 0 | 1.019 | male | 70.334 | MX | N0 | T2a | Stage II |
| TCGA-C4-A0EZ | 1 | 0.748 | female | 69.047 | M1 | N1 | T3a | Stage IV |
| TCGA-FD-A62S | 1 | 1.112 | female | 60.274 | MX | N0 | T3b | Stage III |
| TCGA-FD-A5C0 | 1 | 1.507 | male | 61.644 | MX | N2 | T3a | Stage IV |
| TCGA-GU-A42R | 1 | 1.581 | male | 68.86 | MX | N2 | T4a | Stage IV |
| TCGA-UY-A78M | 1 | 1.89 | female | 81.386 | MX | N2 | T2 | Stage IV |
| TCGA-GV-A3QI | 0 | 3.041 | male | 47.625 | MX | N0 | T3b | Stage III |
| TCGA-FD-A5C1 | 0 | 4.91 | female | 61.658 | MX | N0 | T3b | Stage III |
| TCGA-DK-AA6X | 0 | 1.279 | male | 63.584 | M0 | N0 | '-- | Stage II |
| TCGA-CU-A3YL | 0 | 2.482 | male | 67.189 | M0 | N0 | T4a | Stage III |
| TCGA-FD-A6TH | 1 | 0.359 | male | 63.66 | MX | N2 | T3 | Stage IV |
| TCGA-BT-A20T | 1 | 1.241 | male | 63.616 | M0 | N1 | T3b | Stage IV |
| TCGA-FD-A6TG | 1 | 0.255 | male | 73.679 | MX | N2 | T3a | Stage IV |
| TCGA-DK-A6B1 | 0 | 5.614 | male | 67.416 | M0 | N0 | T2a | Stage II |
| TCGA-ZF-AA58 | 0 | 4.518 | female | 61.778 | MX | N2 | T3a | Stage IV |
| TCGA-CF-A47T | 1 | 1.055 | female | 58.485 | M0 | N0 | T2 | Stage II |
| TCGA-XF-AAMH | 1 | 0.942 | male | 80.951 | MX | N1 | T3b | Stage IV |
| TCGA-BL-A3JM | 1 | 0.562 | male | 63.014 | M0 | N0 | T3 | Stage III |
| TCGA-FD-A6TI | 1 | 0.805 | male | 73.627 | MX | N1 | T4b | Stage IV |
| TCGA-FD-A3SN | 0 | 2.43 | male | 79.915 | MX | N0 | T3b | Stage III |
| TCGA-ZF-AA51 | 0 | 4.696 | female | 69.819 | M0 | NX | T2 | Stage II |
| TCGA-GV-A40G | 0 | 1.589 | male | 77.271 | MX | N0 | T2a | Stage II |
| TCGA-DK-AA6U | 0 | 1.584 | male | 64.688 | M0 | N0 | '-- | Stage II |
| TCGA-DK-A6B2 | 0 | 1.307 | male | 70.205 | M0 | N1 | T3 | Stage IV |
| TCGA-CF-A47W | 0 | 1.008 | male | 42.458 | M0 | N0 | T2 | Stage II |
| TCGA-FD-A43U | 0 | 1.742 | male | 70.797 | MX | N2 | T4a | Stage IV |
| TCGA-FD-A3NA | 0 | 5.055 | male | 60.099 | MX | N0 | T2b | Stage II |
| TCGA-DK-A3IL | 1 | 1.132 | female | 79.581 | M0 | N2 | T3 | Stage IV |
| TCGA-XF-A8HC | 1 | 0.548 | male | 79.441 | MX | N2 | T3a | Stage IV |
| TCGA-ZF-AA4T | 1 | 1.641 | male | 65.682 | MX | N2 | T4 | Stage IV |
| TCGA-E7-A97P | 1 | 1.197 | male | 73.852 | M0 | NX | T2 | Stage II |
| TCGA-XF-AAMW | 1 | 0.693 | female | 79.186 | '-- | N0 | T2b | Stage IV |
| TCGA-E5-A4U1 | 0 | 3.236 | male | 72.068 | M0 | N0 | T2b | Stage II |
| TCGA-E7-A8O7 | 0 | 1.277 | male | 52.227 | M0 | N0 | T2 | Stage II |
| TCGA-CF-A3MF | 0 | 1.049 | male | 34.403 | M0 | N0 | T3 | Stage III |
| TCGA-4Z-AA7M | 0 | 1.356 | male | 65.252 | M0 | N0 | T3a | Stage III |
| TCGA-GC-A3OO | 0 | 1.318 | male | 79.63 | M0 | N0 | T2b | Stage II |
| TCGA-K4-A4AC | 1 | 0.762 | male | 83.986 | MX | N0 | T2b | Stage II |
| TCGA-GU-A764 | 0 | 1.671 | male | 66.693 | MX | N0 | T2b | Stage II |
| TCGA-G2-AA3B | 0 | 5.501 | female | 75.742 | M0 | N0 | T2 | Stage II |
| TCGA-BT-A0YX | 1 | 1.096 | female | 70.055 | M0 | N0 | T3b | Stage III |
| TCGA-DK-AA71 | 0 | 1.137 | male | 71.096 | M0 | N0 | T2b | Stage II |
| TCGA-DK-A2I2 | 1 | 0.649 | female | 63.332 | M0 | N3 | T3 | Stage IV |
| TCGA-XF-AAN3 | 0 | 7.192 | male | 76.463 | MX | N2 | T3b | Stage IV |
| TCGA-ZF-AA5P | 0 | 1.019 | male | 65.674 | M0 | N2 | T2b | Stage IV |
| TCGA-BT-A20V | 1 | 0.422 | female | 59.044 | M0 | N2 | T4a | Stage IV |
| TCGA-CF-A7I0 | 0 | 1.008 | male | 54.956 | M0 | N0 | T2 | Stage II |
| TCGA-2F-A9KQ | 0 | 7.907 | male | 69.203 | M0 | N0 | T3a | Stage III |
| TCGA-ZF-A9RE | 1 | 0.29 | female | 78.416 | MX | '-- | '-- | Stage II |
| TCGA-E7-A7XN | 0 | 1.173 | male | 67.03 | M0 | N0 | T3 | Stage III |
| TCGA-G2-A2EC | 1 | 1.907 | female | 58.123 | M0 | N0 | '-- | Stage II |
| TCGA-E7-A678 | 0 | 2.186 | male | 55.258 | M0 | N0 | T3 | Stage III |
| TCGA-G2-A2EF | 0 | 5.162 | male | 50.595 | M0 | N0 | '-- | Stage II |
| TCGA-GD-A3OS | 0 | 1.748 | female | 54.181 | MX | NX | '-- | Stage II |
| TCGA-DK-A3WW | 0 | 1.734 | male | 57.285 | M0 | N0 | T3 | Stage III |
| TCGA-GD-A3OQ | 0 | 0.26 | male | 48.444 | MX | N1 | T4a | Stage IV |
| TCGA-FD-A3SQ | 1 | 3.899 | male | 62.156 | MX | N2 | T3a | Stage IV |
| TCGA-YF-AA3M | 0 | 1.137 | male | 57.8 | MX | NX | T2a | Stage II |
| TCGA-G2-A2EL | 1 | 2.244 | male | 77.726 | M0 | N0 | '-- | Stage II |
| TCGA-XF-A9T8 | 1 | 1.145 | male | 64.118 | MX | N0 | T3b | Stage III |
| TCGA-GD-A3OP | 0 | 0.175 | female | 84.811 | MX | N2 | T4a | Stage IV |
| TCGA-XF-AAML | 1 | 0.636 | male | 75.129 | MX | N0 | T2b | Stage II |
| TCGA-4Z-AA82 | 1 | 4.263 | male | 59.981 | M0 | N1 | T2a | Stage IV |
| TCGA-K4-A5RI | 1 | 0.975 | female | 67.436 | MX | N0 | T3a | Stage III |
| TCGA-XF-A9ST | 1 | 0.351 | male | 68.712 | MX | N0 | T3b | Stage III |
| TCGA-GV-A3QH | 1 | 0.707 | male | 67.619 | MX | NX | '-- | Stage II |
| TCGA-HQ-A2OE | 0 | 3.216 | male | #VALUE! | MX | N2 | T2a | '-- |
| TCGA-4Z-AA81 | 1 | 3.479 | male | 80.704 | M0 | N0 | T2b | Stage II |
| TCGA-FD-A3B6 | 1 | 2.753 | male | 75.455 | MX | N0 | T2b | Stage II |
| TCGA-DK-A1AC | 0 | 10.907 | male | 72.074 | M0 | N0 | T3b | Stage III |
| TCGA-ZF-A9R4 | 0 | 2.523 | male | 83.282 | M0 | NX | T2 | Stage II |
| TCGA-K4-AAQO | 0 | 0.984 | male | 56.666 | MX | N0 | T3a | Stage III |
| TCGA-ZF-A9RL | 0 | 7.405 | male | 61.589 | MX | '-- | '-- | Stage II |
| TCGA-FD-A6TB | 0 | 1.567 | male | 82.699 | MX | N0 | T3a | Stage III |
| TCGA-XF-AAMG | 0 | 9.216 | male | 49.373 | MX | N0 | T4a | Stage III |
| TCGA-CF-A1HR | 0 | 1.066 | male | 62.403 | M0 | N0 | T3 | Stage III |
| TCGA-CF-A3MG | 0 | 1.011 | male | 48.219 | M0 | N0 | T2 | Stage II |
| TCGA-LC-A66R | 0 | 1.277 | male | 78.885 | MX | N2 | T4a | Stage IV |
| TCGA-GU-AATP | 0 | 2.748 | male | 74.107 | MX | N2 | T2 | Stage IV |
| TCGA-2F-A9KW | 1 | 0.696 | female | 67.679 | MX | N0 | T3b | Stage III |
| TCGA-UY-A9PF | 0 | 0.321 | male | 78 | MX | N2 | T3a | Stage IV |
| TCGA-FD-A43N | 0 | 1.915 | male | 76.255 | MX | N0 | T3a | Stage III |
| TCGA-GV-A3JW | 0 | 1.778 | male | 74.222 | MX | NX | T2 | Stage II |
| TCGA-DK-A1A7 | 0 | 1.534 | female | 67.121 | M0 | N2 | T3 | Stage IV |
| TCGA-E7-A7DV | 0 | 0.101 | male | 44.233 | MX | N3 | T4 | Stage IV |
| TCGA-DK-A3IK | 1 | 0.4 | male | 87.879 | M0 | N2 | T3 | Stage IV |
| TCGA-C4-A0F0 | 0 | 0.162 | male | 60.46 | M0 | N0 | T2b | Stage II |
| TCGA-DK-A3X2 | 1 | 1.499 | male | 85.37 | M0 | N2 | T3 | Stage IV |
| TCGA-E5-A2PC | 0 | 3.633 | female | 61.118 | MX | N1 | T2b | Stage IV |
| TCGA-BT-A20Q | 1 | 1.625 | male | 73.364 | M0 | N2 | T3b | Stage IV |
| TCGA-DK-A3IM | 1 | 0.679 | male | 77.003 | M0 | N0 | T3 | Stage III |
| TCGA-FD-A43P | 0 | 2.279 | male | 74.523 | MX | N0 | T2a | Stage II |
| TCGA-SY-A9G0 | 1 | 2.762 | male | 82.753 | M0 | N1 | T4 | Stage IV |
| TCGA-XF-A9SU | 1 | 0.499 | female | 74.159 | MX | N1 | T3b | Stage IV |
| TCGA-UY-A78O | 0 | 6.334 | female | 74.614 | MX | N0 | T2 | Stage II |
| TCGA-CF-A8HX | 0 | 0.945 | female | 55.504 | M0 | N0 | T2 | Stage II |
| TCGA-FD-A3SL | 1 | 1.951 | male | 60.329 | M1 | N2 | T4a | Stage IV |
| TCGA-4Z-AA87 | 0 | 3.984 | male | 72.411 | M0 | N0 | T4a | Stage III |
| TCGA-DK-AA6S | 0 | 13.836 | male | 60.348 | M0 | N0 | T3b | Stage III |
| TCGA-UY-A9PA | 0 | 2.937 | male | 48 | MX | N0 | T3a | Stage III |
| TCGA-FD-A3B5 | 1 | 0.745 | male | 86.142 | MX | N1 | T2b | Stage IV |
| TCGA-FD-A62O | 1 | 0.592 | male | 74.822 | MX | N2 | T3a | Stage IV |
| TCGA-FD-A3SM | 1 | 1.499 | male | 70.827 | M1 | N2 | T3a | Stage IV |
| TCGA-GV-A3QF | 1 | 1.69 | male | 79.151 | MX | N2 | T3b | Stage IV |
| TCGA-DK-A1AE | 0 | 1.345 | male | 84.129 | M0 | N0 | T3 | Stage III |
| TCGA-4Z-AA7Y | 0 | 4.17 | male | 60.427 | M0 | N0 | T2a | Stage II |
| TCGA-CU-A0YO | 1 | 0.408 | male | 84.43 | MX | N2 | T3a | Stage IV |
| TCGA-FD-A3B3 | 1 | 2.668 | female | 74.159 | MX | N0 | T3 | Stage III |
| TCGA-CF-A5U8 | 0 | 1.093 | male | 59.545 | M0 | N0 | T2 | Stage II |
| TCGA-GC-A3YS | 0 | 2.077 | male | 61.685 | MX | N1 | T3a | Stage IV |
| TCGA-BT-A20W | 1 | 0.696 | male | 71.447 | M0 | N0 | T2b | Stage II |
| TCGA-DK-AA6P | 0 | 1.252 | female | 72.767 | M0 | N0 | '-- | Stage II |
| TCGA-ZF-A9RC | 0 | 7.858 | male | 77.466 | MX | N0 | T3a | Stage III |
| TCGA-FD-A5BV | 1 | 0.447 | female | 47.948 | MX | N0 | T3b | Stage III |
| TCGA-5N-A9KI | 1 | 0.208 | female | 76.101 | MX | NX | T4 | Stage III |
| TCGA-FD-A43X | 0 | 0.301 | male | 84.285 | MX | N0 | T2a | Stage II |
| TCGA-C4-A0F6 | 0 | 1.918 | female | 82.058 | M0 | N0 | T3b | Stage III |
| TCGA-XF-A9SL | 1 | 5.534 | male | 69.753 | MX | N2 | T3a | Stage IV |
| TCGA-K4-A54R | 0 | 2.307 | male | 59.181 | MX | N0 | T2b | Stage II |
| TCGA-XF-AAN1 | 1 | 2.578 | female | 75.855 | M0 | N0 | T4a | Stage III |
| TCGA-FD-A3B8 | 0 | 1.052 | male | 56.315 | MX | N0 | T2b | Stage II |
| TCGA-BL-A13J | 1 | 0.222 | male | 65.553 | M0 | N2 | T4 | Stage IV |
| TCGA-DK-A1AA | 0 | 1.584 | male | 57.789 | M0 | N0 | T3 | Stage III |
| TCGA-UY-A78N | 1 | 7.236 | male | 79.814 | MX | N1 | T2 | Stage IV |
| TCGA-DK-A3IT | 0 | 1.775 | male | 62.427 | M0 | N0 | T3 | Stage III |
| TCGA-ZF-AA4U | 1 | 0.718 | male | 70.362 | MX | NX | T4a | Stage III |
| TCGA-K4-A5RJ | 0 | 1.477 | male | 75.392 | MX | N0 | T2b | Stage II |
| TCGA-ZF-A9R0 | 1 | 1.863 | male | 82.8 | M0 | NX | T3b | Stage III |
| TCGA-DK-A2I4 | 0 | 10.507 | male | 79.553 | M0 | N0 | T3b | Stage III |
| TCGA-FD-A6TE | 0 | 1.03 | male | 54.449 | MX | N2 | T3a | Stage IV |
| TCGA-GD-A2C5 | 0 | 2.225 | female | 53.419 | MX | N2 | T3a | Stage IV |
| TCGA-G2-A2EK | 0 | 1.329 | male | 57.521 | M0 | N0 | '-- | Stage II |
| TCGA-DK-AA6R | 0 | 13.811 | male | 69.03 | M0 | N1 | T4 | Stage IV |
| TCGA-UY-A78K | 1 | 1.468 | male | 60.329 | MX | N2 | '-- | Stage IV |
| TCGA-XF-A9T0 | 0 | 2.189 | male | 69.014 | MX | N0 | T3b | Stage III |
| TCGA-XF-A9T3 | 0 | 0.186 | female | 69.951 | MX | N2 | T3b | Stage IV |
| TCGA-FD-A3SP | 0 | 2.145 | male | 60.288 | MX | N0 | T3b | Stage III |
| TCGA-DK-AA6Q | 1 | 1.132 | female | 61.134 | M0 | N1 | T3 | Stage IV |
| TCGA-4Z-AA89 | 0 | 2.819 | male | 61.016 | M0 | N0 | T4b | Stage IV |
| TCGA-K4-A6FZ | 0 | 0.151 | female | 75.852 | MX | N0 | T3a | Stage III |
| TCGA-FD-A6TF | 1 | 0.189 | female | 80.321 | M1 | N2 | T3b | Stage IV |
| TCGA-ZF-AA4W | 0 | 5.014 | male | 56.468 | MX | N0 | T3b | Stage III |
| TCGA-DK-AA6W | 1 | 1.137 | male | 79.049 | M0 | N0 | T2 | Stage II |
| TCGA-FD-A6TK | 0 | 0.904 | male | 60.115 | MX | N0 | T3a | Stage III |
| TCGA-2F-A9KP | 1 | 0.997 | male | 66.926 | MX | N2 | T3a | Stage IV |
| TCGA-YF-AA3L | 0 | 0.997 | female | 47.31 | MX | N0 | T2b | Stage II |
| TCGA-K4-A6MB | 0 | 1.285 | male | 64.833 | MX | N1 | T3b | Stage IV |
| TCGA-CU-A0YN | 1 | 1.077 | male | 60.074 | M0 | N0 | T3a | Stage III |
| TCGA-KQ-A41N | 0 | 4.395 | male | 73.526 | M0 | N0 | T3b | Stage III |
| TCGA-K4-A3WS | 0 | 2.085 | male | 66.093 | MX | N0 | T3a | Stage III |
| TCGA-BT-A0S7 | 1 | 0.548 | male | 75.433 | MX | N0 | T4a | Stage III |
| TCGA-R3-A69X | 0 | 1.186 | male | 70.071 | M0 | N0 | T3a | Stage III |
| TCGA-GU-A762 | 1 | 0.636 | male | 87.156 | MX | N2 | T4a | Stage IV |
| TCGA-GC-A3RD | 0 | 1.173 | female | 83.641 | M0 | N0 | T3a | Stage III |
| TCGA-CF-A8HY | 0 | 0.945 | male | 59.277 | M0 | N0 | T2 | Stage II |
| TCGA-LT-A8JT | 0 | 1.756 | female | 69.858 | M0 | N0 | T2a | Stage II |
| TCGA-XF-A9SI | 0 | 6.638 | female | 73.203 | MX | N0 | T2b | Stage II |
| TCGA-FJ-A3Z7 | 0 | 2.589 | male | 76.855 | MX | N2 | T4a | Stage IV |
| TCGA-FD-A5BX | 1 | 0.474 | male | 82.948 | MX | N1 | T3b | Stage IV |
| TCGA-DK-A3X1 | 0 | 5.504 | female | 78.786 | M0 | N0 | T3 | Stage III |
| TCGA-ZF-AA52 | 1 | 2.951 | male | 70.079 | MX | NX | T3a | Stage III |
| TCGA-FD-A3SJ | 1 | 2.025 | male | 59.219 | MX | N2 | T2b | Stage IV |
| TCGA-XF-A9T6 | 0 | 0.175 | female | 88.238 | MX | N0 | T3b | Stage III |
| TCGA-FD-A3SR | 1 | 1.649 | male | 68.436 | MX | N2 | T4a | Stage IV |
| TCGA-G2-A2ES | 1 | 2.751 | male | 85.734 | M0 | N0 | T3b | Stage II |
| TCGA-4Z-AA7R | 1 | 1.43 | male | 73.373 | M0 | N0 | T4b | Stage IV |
| TCGA-FJ-A3Z9 | 1 | 1.055 | male | 72.644 | M0 | N0 | TX | '-- |
| TCGA-BT-A3PK | 1 | 0.83 | male | 80.312 | MX | N0 | T2b | Stage II |
| TCGA-CF-A9FF | 0 | 0.989 | male | 52.589 | M0 | N0 | T2 | Stage II |
| TCGA-DK-A6B0 | 0 | 6.384 | male | 61.477 | M0 | N0 | T2b | Stage II |
| TCGA-ZF-A9RN | 1 | 1.685 | female | 67.932 | M0 | NX | T3b | Stage III |
| TCGA-FD-A62P | 1 | 0.523 | male | 76.282 | MX | N0 | T2b | Stage II |
| TCGA-E7-A519 | 0 | 1.392 | male | 72.403 | M0 | NX | T2b | Stage II |
| TCGA-BT-A20J | 1 | 1.586 | male | 75.742 | MX | N0 | T2b | Stage II |
| TCGA-GC-A3I6 | 1 | 1.726 | male | 45.216 | M0 | N0 | T3a | Stage III |
| TCGA-XF-A9SP | 1 | 1.244 | male | 59.66 | MX | N0 | T3b | Stage III |
| TCGA-S5-A6DX | 1 | 0.153 | male | 83.819 | MX | N2 | T4a | Stage IV |
| TCGA-FD-A43S | 0 | 1.247 | female | 71.214 | MX | N0 | T3b | Stage III |
| TCGA-GC-A3BM | 1 | 1.784 | male | 70.162 | M0 | N0 | T2b | Stage II |
| TCGA-4Z-AA7N | 1 | 3.745 | male | 66 | M0 | N0 | T3a | Stage III |
| TCGA-4Z-AA83 | 0 | 5.545 | male | 52.822 | M0 | N0 | T2a | Stage II |
| TCGA-BT-A2LA | 0 | 1.43 | male | 54.882 | M0 | N0 | T3a | Stage III |
| TCGA-CU-A5W6 | 1 | 0.153 | male | 70.427 | M0 | N0 | T4a | Stage III |
| TCGA-FD-A3SS | 1 | 1.071 | male | 66.871 | MX | N3 | T4 | Stage IV |
| TCGA-FT-A61P | 0 | 0.923 | male | 76.937 | MX | N2 | T3b | Stage IV |
| TCGA-SY-A9G5 | 0 | 3.249 | male | 66.466 | M0 | N0 | T4a | Stage III |
| TCGA-ZF-A9RM | 0 | 3.986 | male | 70.252 | MX | N0 | T0 | Stage II |
| TCGA-CF-A27C | 0 | 1.164 | male | 52.795 | M0 | N0 | T3 | Stage III |
| TCGA-CU-A0YR | 1 | 1.26 | male | 84.038 | M0 | N2 | T2 | Stage IV |
| TCGA-XF-AAMR | 0 | 7.644 | male | 48.411 | MX | N0 | T3b | Stage III |
| TCGA-4Z-AA7W | 0 | 2.301 | male | 55.956 | M0 | N0 | T2a | Stage II |
| TCGA-CF-A47X | 0 | 1.052 | male | 60.822 | M0 | N0 | T2 | Stage II |
| TCGA-XF-AAMQ | 0 | 5.964 | female | 59.342 | MX | N0 | T2a | Stage II |
| TCGA-4Z-AA7O | 0 | 1.403 | male | 64.781 | M0 | N0 | T2a | Stage II |
| TCGA-BT-A20U | 1 | 1.247 | female | 70.578 | M0 | N0 | T3a | Stage III |
| TCGA-XF-A8HE | 0 | 10.458 | male | 47.156 | MX | N0 | T3b | Stage III |
| TCGA-XF-A8HF | 1 | 8.093 | male | 80.241 | M0 | N0 | T3a | Stage III |
| TCGA-H4-A2HO | 0 | 0.126 | male | 53.781 | MX | N0 | T4a | Stage III |
| TCGA-DK-A1AD | 0 | 9.37 | male | 69.362 | M0 | N2 | T3b | Stage IV |
| TCGA-FD-A3B4 | 1 | 1.397 | female | 55.833 | MX | N0 | T4a | Stage III |
| TCGA-XF-A9SY | 0 | 1.753 | female | 60.855 | MX | N2 | T3b | Stage IV |
| TCGA-4Z-AA84 | 0 | 1.26 | male | 61.362 | M1 | N2 | T3a | Stage IV |
| TCGA-FD-A3N6 | 0 | 2.332 | female | 43.173 | MX | N0 | T2b | Stage II |
| TCGA-BT-A42E | 0 | 3.036 | male | 74.096 | M0 | N0 | T3a | Stage III |
| TCGA-FD-A5BY | 0 | 0.688 | female | 63.233 | MX | N0 | T3a | Stage III |
| TCGA-XF-AAMJ | 1 | 4.575 | male | 70.789 | M0 | N0 | T3b | Stage III |
| TCGA-DK-A1A5 | 1 | 0.178 | male | 79.756 | M0 | N0 | T2b | Stage II |
| TCGA-XF-A8HB | 0 | 3.753 | female | 50.805 | MX | N0 | T2b | Stage II |
| TCGA-FJ-A3ZE | 1 | 0.888 | male | 65.386 | M1 | N3 | '-- | Stage IV |
| TCGA-E7-A4XJ | 1 | 0.186 | male | 66.518 | M0 | N0 | T2 | Stage II |
| TCGA-ZF-AA53 | 0 | 4.825 | male | 60.37 | M0 | NX | T2 | Stage II |
| TCGA-E7-A6MD | 0 | 0.353 | male | 66.247 | M0 | N1 | T4a | Stage IV |
| TCGA-BT-A3PH | 1 | 0.389 | male | 76.795 | MX | N2 | T3b | Stage IV |
| TCGA-4Z-AA86 | 1 | 0.852 | male | 67.027 | M0 | N1 | T3a | Stage IV |
| TCGA-FD-A3SO | 1 | 0.46 | male | 68.896 | MX | N1 | T3a | Stage IV |
| TCGA-YC-A89H | 0 | 1.57 | female | 78.742 | MX | '-- | '-- | Stage II |
| TCGA-CF-A9FL | 1 | 1.548 | male | 85.203 | M0 | N0 | T3b | Stage III |
| TCGA-5N-A9KM | 1 | 1.452 | female | 73.715 | MX | N0 | T4a | Stage III |
| TCGA-YC-A8S6 | 0 | 0.803 | male | 71.589 | MX | N0 | T2a | Stage II |
| TCGA-FD-A5BU | 0 | 1.611 | female | 76.389 | MX | N0 | T2b | Stage II |
| TCGA-XF-AAN2 | 1 | 5.121 | male | 73.205 | MX | N0 | T2b | Stage II |
| TCGA-G2-AA3F | 0 | 2.447 | male | 77.964 | M0 | N1 | T3 | Stage IV |
| TCGA-G2-A2EO | 1 | 4.942 | male | 69.699 | M0 | N0 | T3a | Stage III |
| TCGA-K4-A3WU | 0 | 0.288 | male | 88.038 | MX | N0 | T4a | Stage III |
| TCGA-DK-A3WY | 0 | 13.608 | female | 67.222 | M0 | N0 | T3 | Stage III |
| TCGA-CF-A47Y | 0 | 1.022 | male | 55.978 | M0 | N0 | T2 | Stage II |
| TCGA-DK-A1A6 | 0 | 5.534 | male | 53.573 | M0 | N1 | T2a | Stage IV |
| TCGA-GU-A42Q | 1 | 0.942 | male | 67.216 | M0 | N0 | T3b | Stage III |
| TCGA-GV-A3JZ | 0 | 1.652 | male | 55.945 | MX | N3 | T4a | Stage IV |
| TCGA-XF-AAMY | 0 | 8.249 | male | 78.107 | MX | N0 | T3b | Stage III |
| TCGA-XF-AAN7 | 1 | 1.548 | male | 60.14 | MX | N1 | T3a | Stage IV |
| TCGA-KQ-A41O | 0 | 4.214 | male | 84.312 | M0 | N1 | T3 | Stage IV |
| TCGA-BT-A2LD | 1 | 1.707 | female | 78.556 | M0 | N1 | T3a | Stage IV |
| TCGA-GV-A3QK | 0 | 2.279 | female | 56.992 | M0 | N2 | T4a | Stage IV |
| TCGA-UY-A9PD | 0 | 1.485 | male | 81 | MX | N0 | T3a | Stage III |
| TCGA-E7-A6ME | 0 | 2.003 | male | 75.808 | M0 | NX | T2b | Stage II |
| TCGA-DK-A3IS | 0 | 4.189 | male | 68.762 | M0 | N0 | T2a | Stage II |
| TCGA-GC-A3RB | 0 | 1.595 | male | 54.852 | M0 | N0 | T3b | Stage III |
| TCGA-KQ-A41Q | 0 | 0.989 | male | 89.071 | MX | N0 | T3b | Stage III |
| TCGA-PQ-A6FN | 0 | 1.389 | female | 78.208 | MX | N0 | T3a | Stage III |
| TCGA-S5-AA26 | 0 | 1.378 | male | 46.499 | MX | N0 | T3a | Stage III |
| TCGA-BL-A0C8 | 0 | 3.34 | male | 73.477 | M0 | NX | T1 | Stage I |
| TCGA-XF-A9SX | 1 | 1.97 | female | 63.97 | MX | N2 | T3b | Stage IV |
| TCGA-FD-A3N5 | 1 | 1.877 | male | 69.43 | MX | N0 | T2b | Stage II |
| TCGA-CU-A3KJ | 0 | 1.54 | male | 75.868 | M0 | N0 | T3b | Stage III |
| TCGA-XF-AAN8 | 1 | 0.323 | female | 74.819 | MX | N0 | T3b | Stage III |
| TCGA-K4-A83P | 0 | 1.356 | male | 77.981 | MX | N1 | T4a | Stage IV |
| TCGA-GV-A40E | 1 | 0.715 | male | 75.532 | MX | NX | '-- | Stage II |
| TCGA-XF-A9T2 | 1 | 1.575 | male | 54.644 | MX | N0 | T3b | Stage III |
| TCGA-ZF-A9R5 | 0 | 2.986 | male | 59.756 | M0 | N0 | T3 | Stage III |
| TCGA-CF-A9FM | 0 | 1.09 | male | 50.534 | M0 | N0 | T1 | Stage I |
| TCGA-GD-A6C6 | 0 | 0.184 | male | 64.742 | MX | N0 | T3a | Stage III |
| TCGA-C4-A0F1 | 0 | 0.244 | male | 71.721 | M0 | N0 | T3b | Stage III |
| TCGA-DK-A6B6 | 0 | 3.055 | male | 57.553 | MX | NX | '-- | Stage II |
| TCGA-ZF-A9R3 | 1 | 2.6 | female | 53.107 | M0 | NX | T2 | Stage II |
| TCGA-FD-A5BZ | 1 | 2.288 | female | 77.948 | MX | N1 | T3a | Stage IV |
| TCGA-XF-AAMT | 1 | 0.247 | female | 75.06 | MX | N2 | T3b | Stage IV |
| TCGA-CU-A72E | 1 | 1.132 | male | 76.611 | M0 | N2 | T3b | Stage IV |
| TCGA-CU-A3QU | 0 | 0.433 | male | 58.173 | M0 | N1 | T2b | Stage IV |
| TCGA-ZF-A9R2 | 0 | 1.759 | male | 75.444 | M0 | N0 | T2b | Stage II |
| TCGA-G2-A3IB | 1 | 0.603 | male | 66.162 | MX | NX | '-- | Stage II |
| TCGA-GU-AATQ | 1 | 0.584 | male | 69 | MX | N0 | T3b | Stage III |
| TCGA-XF-A9SM | 0 | 2.871 | male | 76.236 | MX | N0 | T3b | Stage III |
| TCGA-XF-A9SJ | 1 | 0.268 | male | 83.184 | MX | N2 | T3b | Stage IV |
| TCGA-ZF-AA4R | 1 | 2.838 | male | 67.225 | MX | N1 | T3a | Stage IV |
| TCGA-DK-A6AV | 0 | 5.348 | female | 82.436 | M0 | N0 | T2a | Stage II |
| TCGA-CF-A5UA | 0 | 1 | male | 67.466 | M0 | N0 | T2 | Stage II |
| TCGA-FD-A5BS | 0 | 4.49 | male | 68.392 | MX | N0 | T3b | Stage III |
| TCGA-XF-AAN4 | 1 | 2.255 | female | 77.54 | MX | N0 | T3b | Stage III |
| TCGA-FD-A62N | 0 | 0.225 | male | 69.307 | MX | N0 | T3b | Stage III |
| TCGA-XF-A9SH | 1 | 5.4 | female | 65.918 | M0 | N0 | T2b | Stage II |
| TCGA-CF-A3MH | 0 | 1.09 | male | 75.227 | M0 | N0 | T2 | Stage II |
| TCGA-UY-A78P | 0 | 6.521 | female | 78.682 | MX | N0 | T2 | Stage II |
| TCGA-BT-A42C | 0 | 2.392 | male | 64.614 | M0 | N0 | '-- | Stage II |
| TCGA-XF-A8HG | 1 | 1.279 | male | 69.647 | MX | N0 | T3b | Stage III |
| TCGA-FJ-A871 | 1 | 0.745 | male | 49.455 | MX | NX | T3b | Stage III |
| TCGA-2F-A9KT | 0 | 6.444 | male | 83.616 | M0 | N0 | T2b | Stage II |
| TCGA-ZF-AA54 | 1 | 1.616 | male | 71.584 | MX | NX | T3 | Stage III |
| TCGA-GC-A3RC | 0 | 1.326 | male | 59.364 | M0 | N0 | T2b | Stage II |
| TCGA-BT-A20X | 1 | 0.688 | male | 56.216 | M0 | N2 | T4a | Stage IV |
| TCGA-GU-A767 | 1 | 0.395 | male | 81.477 | MX | N2 | T3b | Stage IV |
| TCGA-G2-AA3C | 1 | 0.578 | male | 66.485 | M0 | N1 | T3b | Stage IV |
| TCGA-BT-A20O | 1 | 1.014 | male | 75.436 | MX | N0 | T3a | Stage III |
| TCGA-XF-AAN5 | 0 | 6.282 | female | 61.4 | MX | N0 | T3b | Stage III |
| TCGA-BL-A5ZZ | 0 | 1.033 | female | 80.129 | MX | N0 | T4a | Stage III |
| TCGA-DK-A1A3 | 1 | 1.822 | male | 60.918 | M0 | N2 | T3 | Stage IV |
| TCGA-G2-A2EJ | 0 | 4 | female | 56.573 | M0 | N0 | '-- | Stage II |
| TCGA-G2-A3IE | 1 | 1.677 | male | 52.008 | MX | NX | '-- | Stage II |
| TCGA-DK-A2HX | 1 | 3.89 | female | 80.362 | M0 | N2 | T3 | Stage IV |
| TCGA-FD-A6TD | 1 | 1.058 | male | 77.611 | MX | N0 | T3a | Stage III |
| TCGA-XF-AAME | 1 | 7.748 | female | 64.241 | MX | N0 | T2b | Stage II |
| TCGA-E7-A3X6 | 1 | 2.477 | male | 70.093 | M0 | NX | T2 | Stage II |
| TCGA-E7-A85H | 0 | 1.079 | male | 64.375 | M0 | N0 | T3 | Stage III |
| TCGA-C4-A0F7 | 1 | 0.17 | male | 77.885 | M0 | N2 | T4b | Stage IV |
| TCGA-E5-A4TZ | 1 | 1.279 | male | 64.564 | MX | N2 | T4b | Stage IV |
| TCGA-BT-A3PJ | 0 | 2.162 | male | 76.222 | M0 | N0 | T3b | Stage III |
| TCGA-DK-AA6L | 1 | 3.186 | male | 48.315 | MX | N1 | T3 | Stage IV |
| TCGA-FD-A5BT | 1 | 0.899 | male | 84.071 | MX | N0 | T3b | Stage III |
| TCGA-KQ-A41S | 0 | 0.096 | female | 87.784 | MX | NX | T4a | Stage III |
| TCGA-ZF-AA4V | 0 | 4.948 | male | 66.981 | M0 | N0 | T3b | Stage III |
| TCGA-FT-A3EE | 1 | 0.271 | female | 80.255 | MX | N0 | T4a | Stage III |
| TCGA-UY-A9PH | 0 | 4.277 | male | 73.025 | MX | N0 | T2b | Stage II |
| TCGA-GU-AATO | 1 | 0.888 | male | 75.847 | MX | N2 | T4a | Stage IV |
| TCGA-G2-A3VY | 0 | 1.468 | male | 66.06 | '-- | '-- | '-- | Stage II |
| TCGA-XF-A9SV | 1 | 1.063 | male | 82.918 | M1 | N2 | T4a | Stage IV |
| TCGA-ZF-A9R7 | 0 | 1.822 | female | 76.885 | M0 | NX | '-- | Stage II |
| TCGA-DK-AA75 | 1 | 0.932 | male | 82.438 | M0 | N0 | T3 | Stage III |
| TCGA-H4-A2HQ | 0 | 1.616 | female | 64.488 | M1 | NX | '-- | Stage IV |
| TCGA-BT-A2LB | 1 | 1.348 | female | 73.551 | M0 | N0 | T3a | Stage III |
| TCGA-CF-A47S | 0 | 0.912 | male | 41.712 | M0 | N0 | T2 | Stage II |
| TCGA-MV-A51V | 0 | 1.123 | male | 75.151 | M0 | N0 | T3a | Stage III |
| TCGA-FD-A43Y | 1 | 1.299 | male | 65.449 | MX | N0 | T4 | Stage III |
| TCGA-DK-AA76 | 0 | 1.003 | female | 64.499 | M0 | N0 | '-- | Stage II |
| TCGA-UY-A78L | 0 | 3.088 | male | 62.238 | MX | N1 | '-- | Stage IV |
| TCGA-E7-A677 | 0 | 2.247 | male | 81.132 | M0 | N0 | T2b | Stage II |
| TCGA-E7-A541 | 1 | 2.132 | male | 66.318 | MX | N0 | T2b | Stage II |
| TCGA-K4-A3WV | 0 | 1.77 | female | 77.929 | MX | N0 | T2b | Stage II |
| TCGA-DK-A6B5 | 0 | 4.225 | male | 45.077 | M0 | N2 | T4a | Stage IV |
| TCGA-DK-A3WX | 1 | 0.879 | female | 67.268 | M0 | N0 | T3 | Stage III |
| TCGA-KQ-A41P | 0 | 2.997 | male | 76.208 | M1 | N3 | T3b | Stage IV |
| TCGA-DK-AA6M | 0 | 4.334 | female | 64.586 | M0 | N0 | T2 | Stage II |
| TCGA-GV-A3JX | 0 | 1.592 | male | 59.967 | MX | N0 | T3b | Stage III |
| TCGA-BT-A42F | 0 | 2.367 | male | 64.967 | MX | N1 | T4a | Stage IV |
| TCGA-GC-A3WC | 0 | 1.479 | female | 80.26 | MX | N0 | T3 | Stage III |
| TCGA-XF-A9SZ | 1 | 2.353 | male | 79.726 | MX | N2 | T3b | Stage IV |
| TCGA-2F-A9KO | 1 | 2.011 | male | 63.899 | M0 | N1 | T3 | Stage IV |
| TCGA-DK-A2I6 | 0 | 7.277 | male | 81.614 | M0 | N1 | T2b | Stage IV |
| TCGA-XF-A8HD | 0 | 8.121 | male | 77.548 | MX | N0 | T3a | Stage III |
| TCGA-FD-A5BR | 0 | 2.227 | male | 57.436 | MX | N0 | T2b | Stage II |
| TCGA-HQ-A5NE | 1 | 1.014 | male | 56.567 | M0 | N0 | T3 | Stage III |
| TCGA-G2-AA3D | 0 | 5.86 | male | 60.836 | M0 | N2 | T3 | Stage IV |
| TCGA-DK-A3IQ | 1 | 1.477 | male | 74.504 | M0 | N0 | T3 | Stage III |
| TCGA-KQ-A41R | 0 | 3.699 | female | 80.414 | MX | N0 | T2 | Stage II |
| TCGA-CF-A47V | 0 | 1.038 | male | 52.781 | M0 | N0 | T2 | Stage II |
| TCGA-ZF-AA4X | 0 | 5.6 | male | 56.381 | M0 | NX | T2 | Stage II |
| TCGA-GU-A766 | 0 | 1.315 | male | 62.395 | MX | N0 | T2a | Stage II |
| TCGA-XF-AAN0 | 1 | 4.707 | male | 68.156 | M0 | N2 | T4a | Stage IV |
| TCGA-ZF-AA56 | 1 | 0.71 | female | 79.279 | MX | N0 | T4a | Stage III |
| TCGA-DK-AA77 | 0 | 1.693 | male | 61.274 | M0 | N0 | T2a | Stage II |
| TCGA-ZF-A9R9 | 1 | 2.367 | male | 58.879 | MX | N2 | T3b | Stage IV |
| TCGA-BL-A13I | 1 | 0.611 | female | 57.219 | M0 | N0 | T3 | Stage III |
| TCGA-BT-A20N | 1 | 2.178 | male | 72.482 | MX | N0 | T3a | Stage III |
| TCGA-LT-A5Z6 | 0 | 1.299 | male | 56.838 | MX | NX | '-- | Stage II |
| TCGA-XF-AAMZ | 1 | 3.693 | female | 81.279 | MX | N2 | T3a | Stage IV |
| TCGA-XF-AAMX | 1 | 0.564 | female | 87.353 | MX | N0 | T3b | Stage III |
| TCGA-K4-A5RH | 0 | 0.756 | male | 69.329 | MX | N0 | T3a | Stage III |
| TCGA-XF-A8HI | 1 | 1.49 | female | 57.696 | MX | N2 | T2b | Stage IV |
| TCGA-FD-A3B7 | 1 | 0.334 | male | 66.425 | MX | N0 | T3a | Stage III |
| TCGA-4Z-AA7S | 1 | 2.915 | male | 66.058 | M0 | N0 | T4a | Stage III |
| TCGA-DK-A1AB | 1 | 1.392 | female | 74.318 | M0 | N2 | T4a | Stage IV |
| TCGA-DK-A3IU | 1 | 1.934 | male | 58.545 | M0 | N0 | T2b | Stage II |
| TCGA-ZF-A9R1 | 0 | 2.118 | male | 81.123 | M0 | N1 | T3b | Stage IV |
| TCGA-CF-A3MI | 0 | 1.014 | male | 62.236 | M0 | N0 | T2 | Stage II |
| TCGA-DK-A1AF | 0 | 1.468 | female | 84.696 | M0 | N2 | T3 | Stage IV |
| TCGA-BT-A20P | 1 | 1.49 | female | 81.189 | M0 | N0 | T3a | Stage III |
| TCGA-ZF-A9RD | 1 | 1.118 | female | 75.332 | MX | N2 | T3a | Stage IV |
| TCGA-GV-A6ZA | 0 | 1.893 | male | 54.441 | MX | N0 | T2b | Stage II |
| TCGA-XF-A9SW | 1 | 0.992 | male | 85.066 | MX | N2 | T3b | Stage IV |
| TCGA-GU-A763 | 0 | 2.732 | male | 72.677 | M0 | N0 | T4 | Stage III |
| TCGA-2F-A9KR | 1 | 8.721 | female | 59.858 | M0 | N0 | T3a | Stage III |
| TCGA-ZF-AA5N | 1 | 0.46 | female | 62.304 | M1 | NX | T2 | Stage IV |
| TCGA-E7-A3Y1 | 0 | 0.447 | male | 57.307 | M0 | N0 | '-- | Stage II |
| TCGA-BT-A20R | 1 | 0.422 | female | 79.416 | M0 | N1 | T3b | Stage IV |
| TCGA-DK-A1AG | 0 | 1.301 | male | 66.014 | M0 | N0 | T3 | Stage III |
| TCGA-UY-A9PB | 0 | 2.463 | male | 49 | MX | N0 | T3a | Stage III |
| TCGA-DK-A3IV | 1 | 0.805 | male | 60.923 | M0 | NX | '-- | Stage II |
| TCGA-XF-A9T4 | 1 | 1.356 | male | 48.57 | MX | N1 | T2b | Stage IV |
| TCGA-XF-A9T5 | 0 | 5.553 | female | 78.296 | MX | N1 | T3a | Stage IV |
| TCGA-DK-AA6T | 0 | 1.567 | male | 63.556 | M0 | N0 | '-- | Stage II |
| TCGA-ZF-A9RF | 0 | 5.34 | male | 74.742 | M0 | NX | T1 | Stage II |
| TCGA-UY-A8OD | 0 | 9.403 | female | 67.364 | MX | N0 | T2b | Stage II |
| TCGA-FJ-A3ZF | 0 | 1.436 | male | 73.493 | M0 | N0 | '-- | Stage III |
| TCGA-E7-A6MF | 0 | 2.055 | male | 37.992 | M0 | NX | T2a | Stage II |
| TCGA-E7-A4IJ | 1 | 1.847 | male | 56.775 | M0 | NX | T2b | Stage II |
| TCGA-DK-A3IN | 1 | 0.685 | male | 72.773 | M0 | N0 | T4a | Stage III |
| TCGA-FD-A6TA | 0 | 5.238 | male | 58.071 | MX | N2 | T3b | Stage IV |
| TCGA-UY-A8OB | 0 | 5.778 | male | 64 | MX | N1 | T3a | Stage IV |
| TCGA-UY-A9PE | 0 | 0.518 | male | 87 | MX | N2 | T2b | Stage IV |
| TCGA-GU-A42P | 1 | 0.91 | male | 72.729 | M0 | N1 | T3a | Stage IV |
| TCGA-4Z-AA7Q | 1 | 1.397 | male | 79.4 | M0 | NX | T3a | Stage III |
