# Supplementary material for: Targeting the SMAD3/CISD2 axis suppresses bladder cancer progression by promoting ferroptosis in mesenchymal-like bladder cancer cells
Source: Cell Death Dis. 2025 Dec 18;17(1):101. doi: 10.1038/s41419-025-08339-9 (PMC12847906; doi:10.1038/s41419-025-08339-9)

Marker:

Thermo Scientific™
PageRuler Prestained Protein Ladder
Cat# 26616 Marker

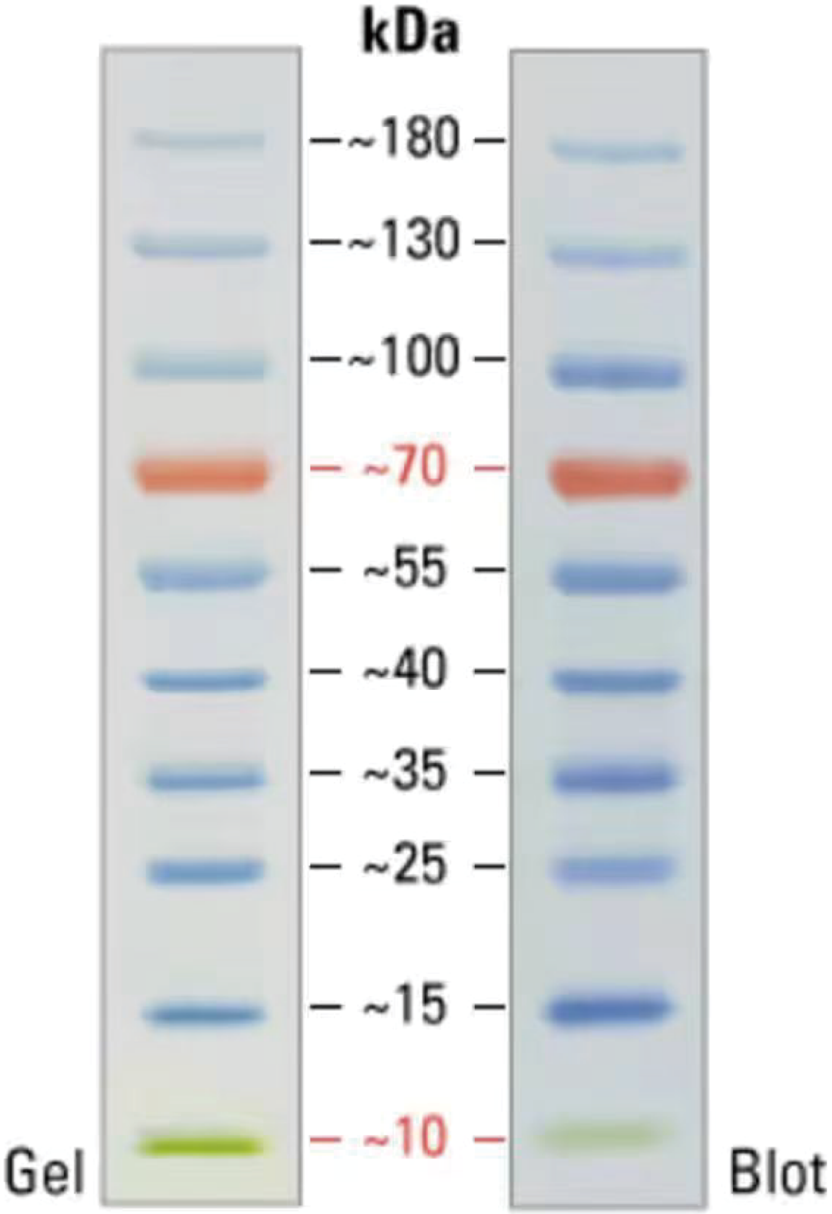

Protein detection:

1.nws12.21 Laboratory
SN:FM1056
Channel： Chemi with Fluor Markers

2.Bio-Rad
ChemiDoc MP imaging system
Catalog Number:12003154

Figure 2A. Example of original western blot for three repeats
T24(left) E-cadherin + GAPDH

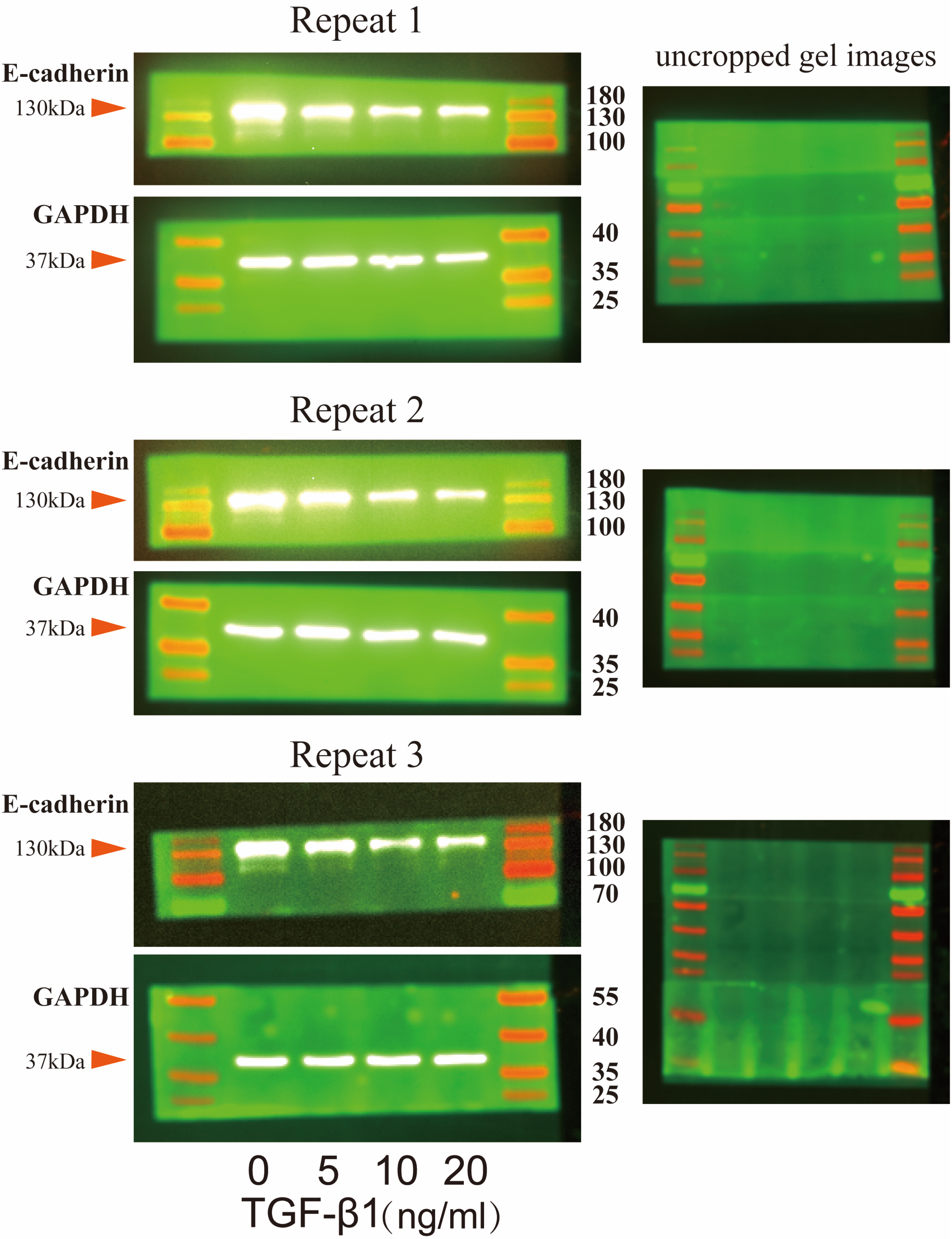

Figure 2A. Example of original western blot for three repeats
T24(left) N-cadherin + GAPDH

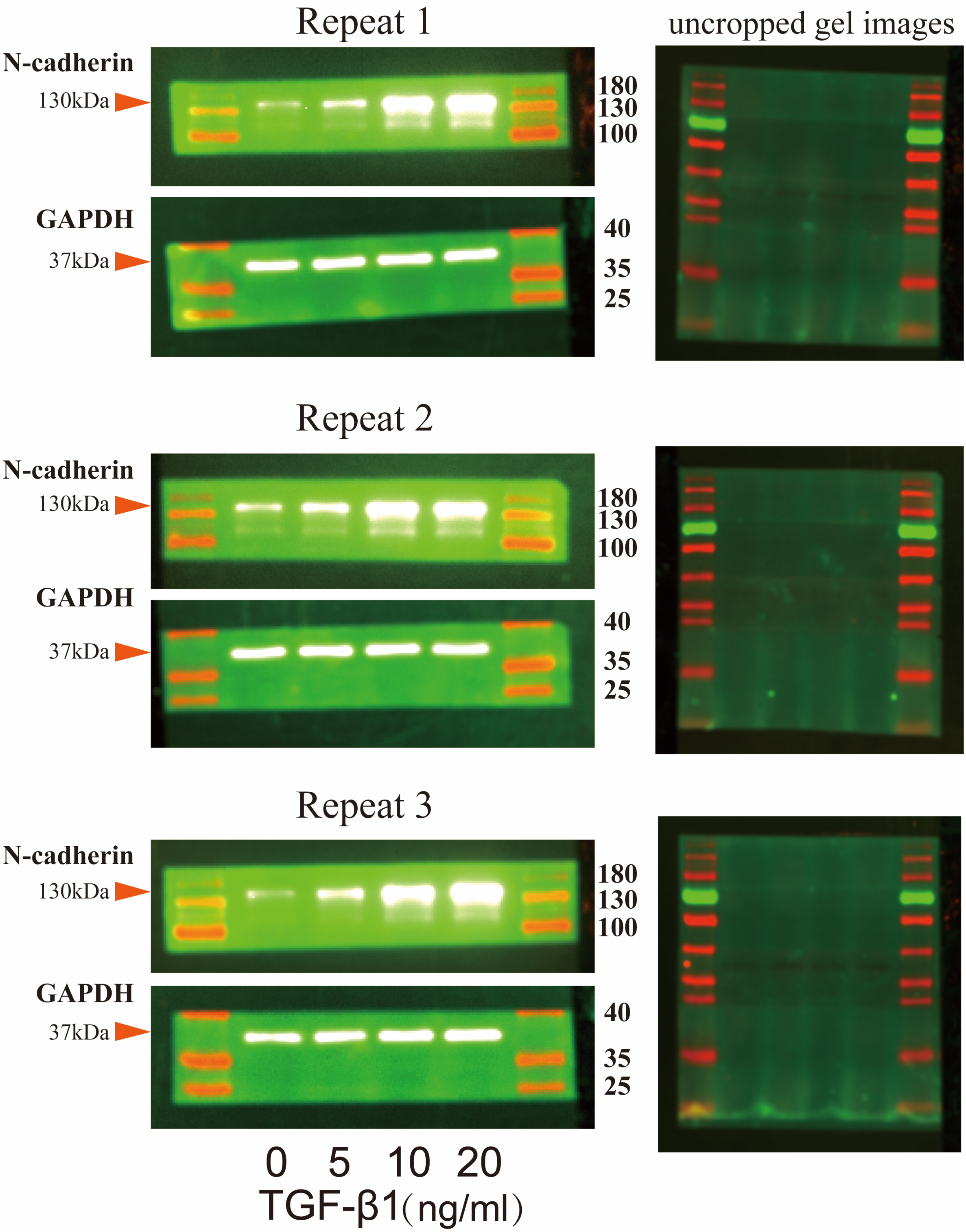

Figure 2A. Example of original western blot for three repeats

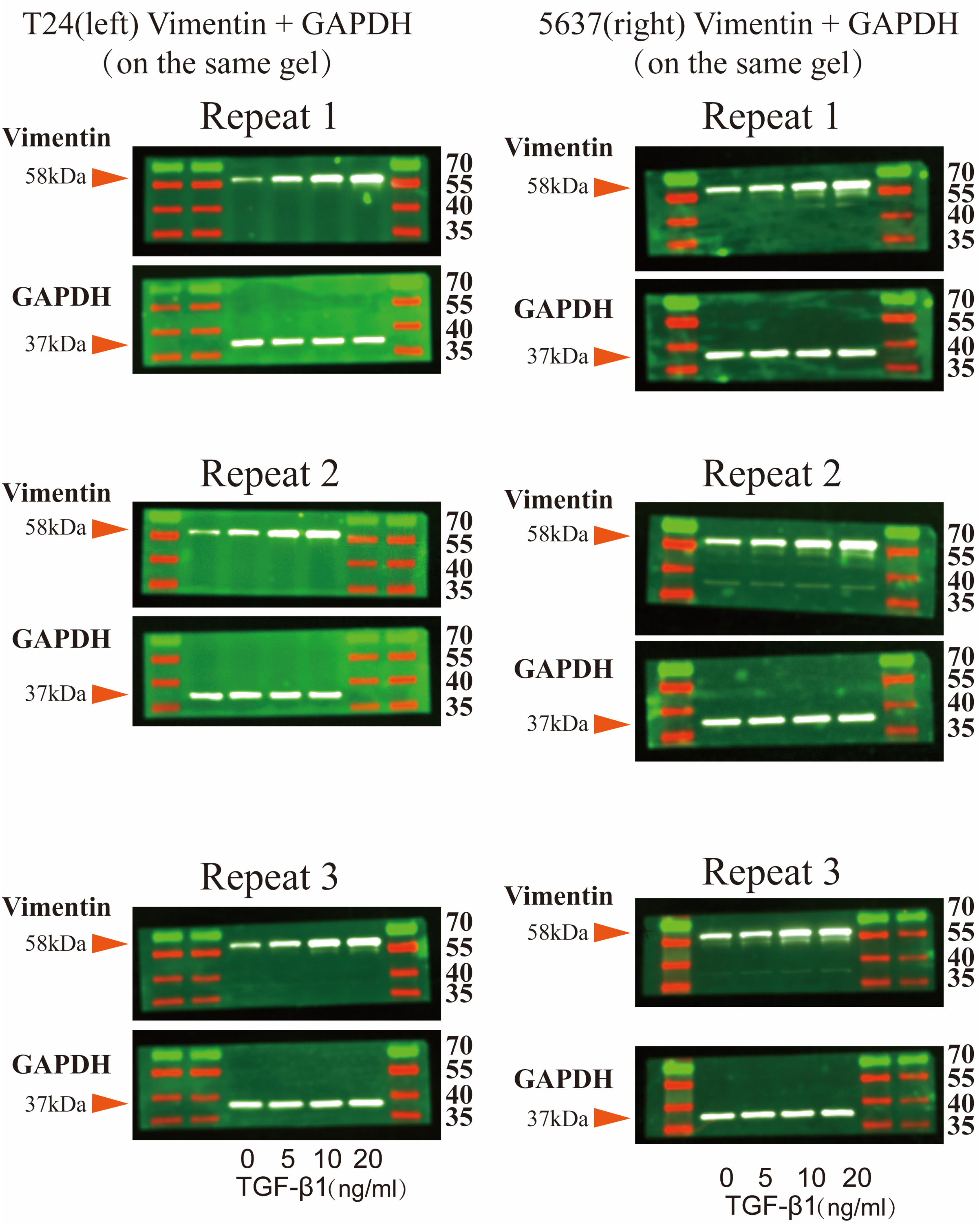

Figure 2A. Example of original western blot for three repeats
5637(right) E-cadherin + GAPDH

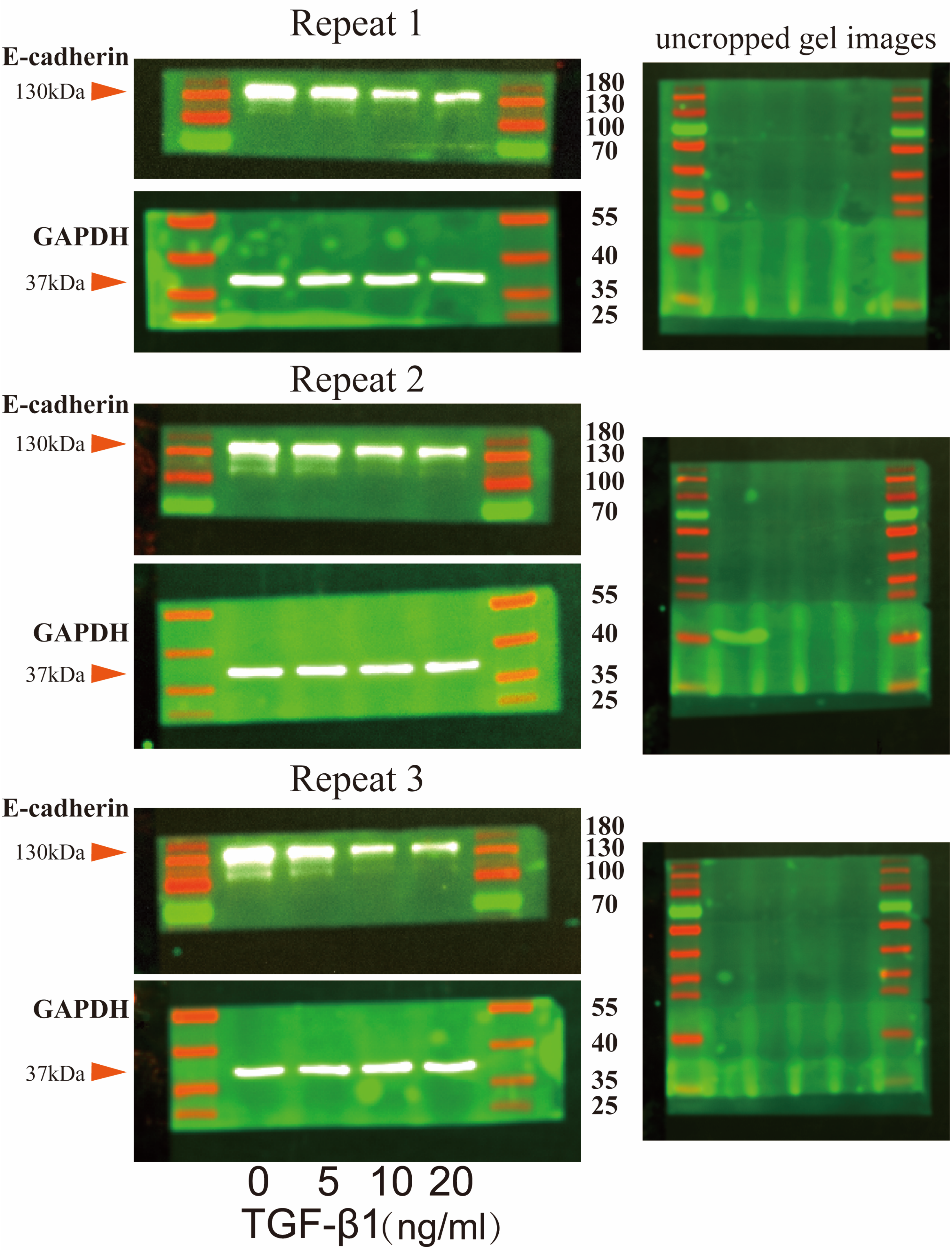

Figure 2A. Example of original western blot for three repeats

5637(right) N-cadherin + GAPDH

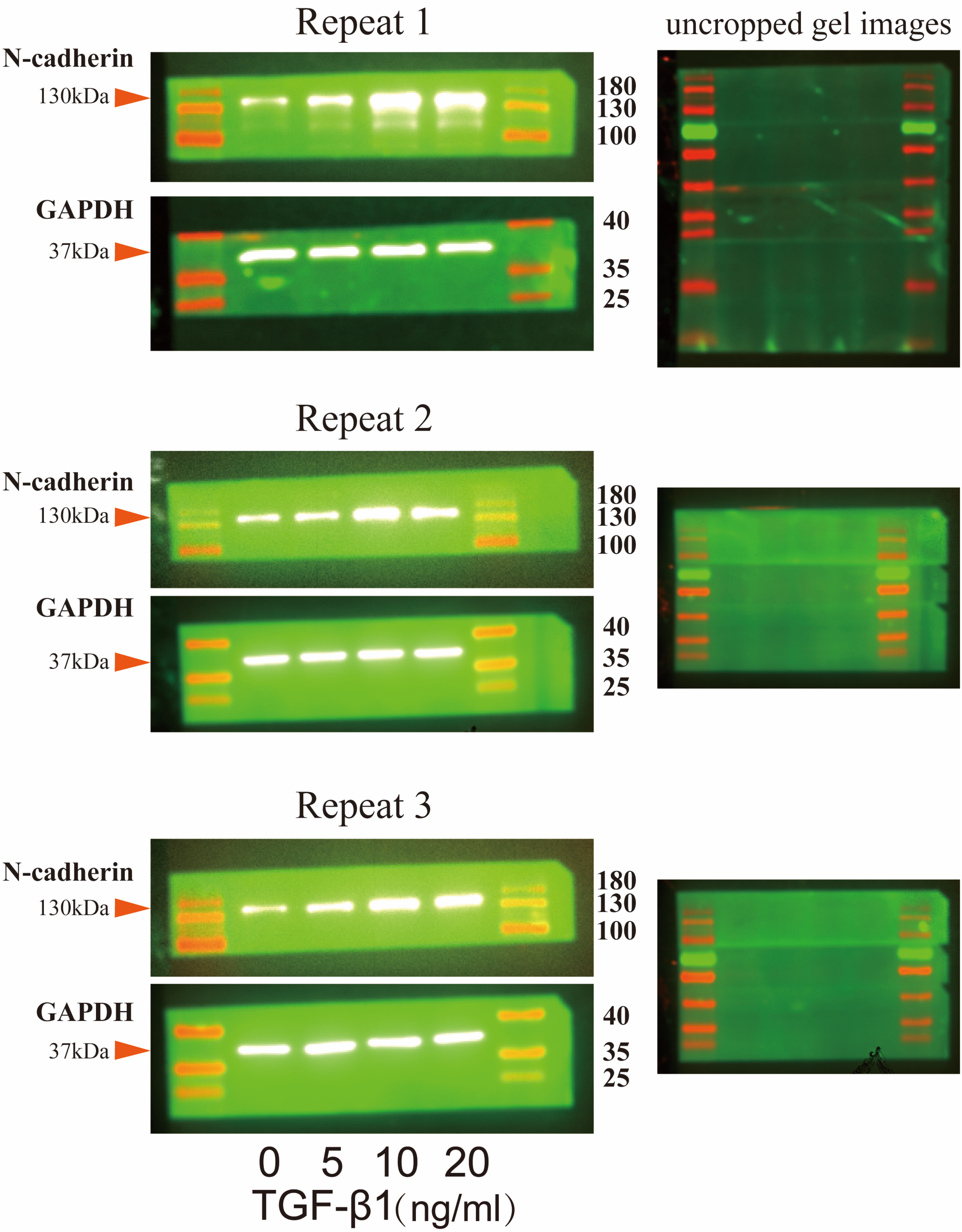

Figure 2I. Example of original western blot for three repeats
T24(left) GPX4 + GAPDH

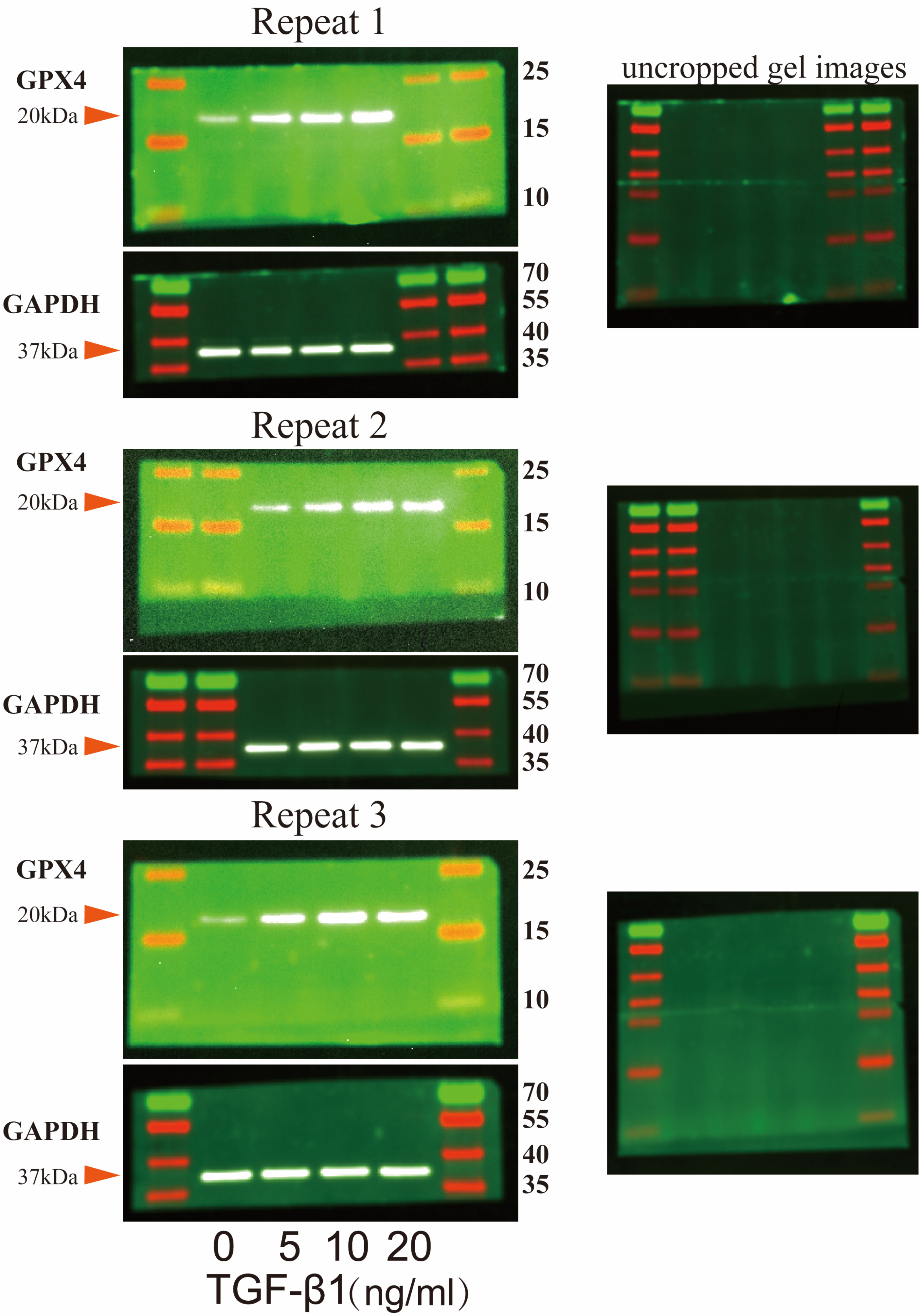

Figure 2I. Example of original western blot for three repeats

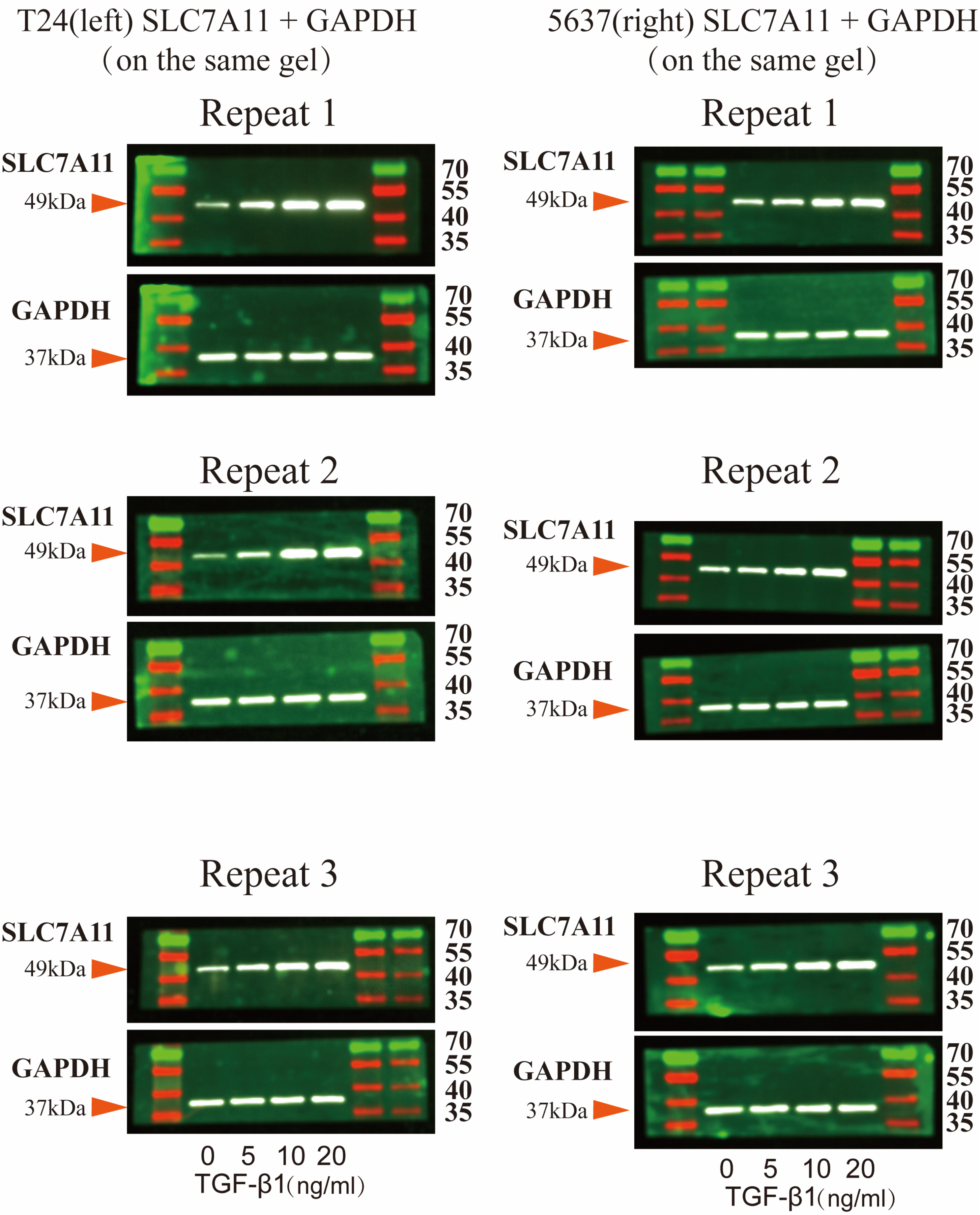

Figure 2I. Example of original western blot for three repeats
5637(right) GPX4 + GAPDH

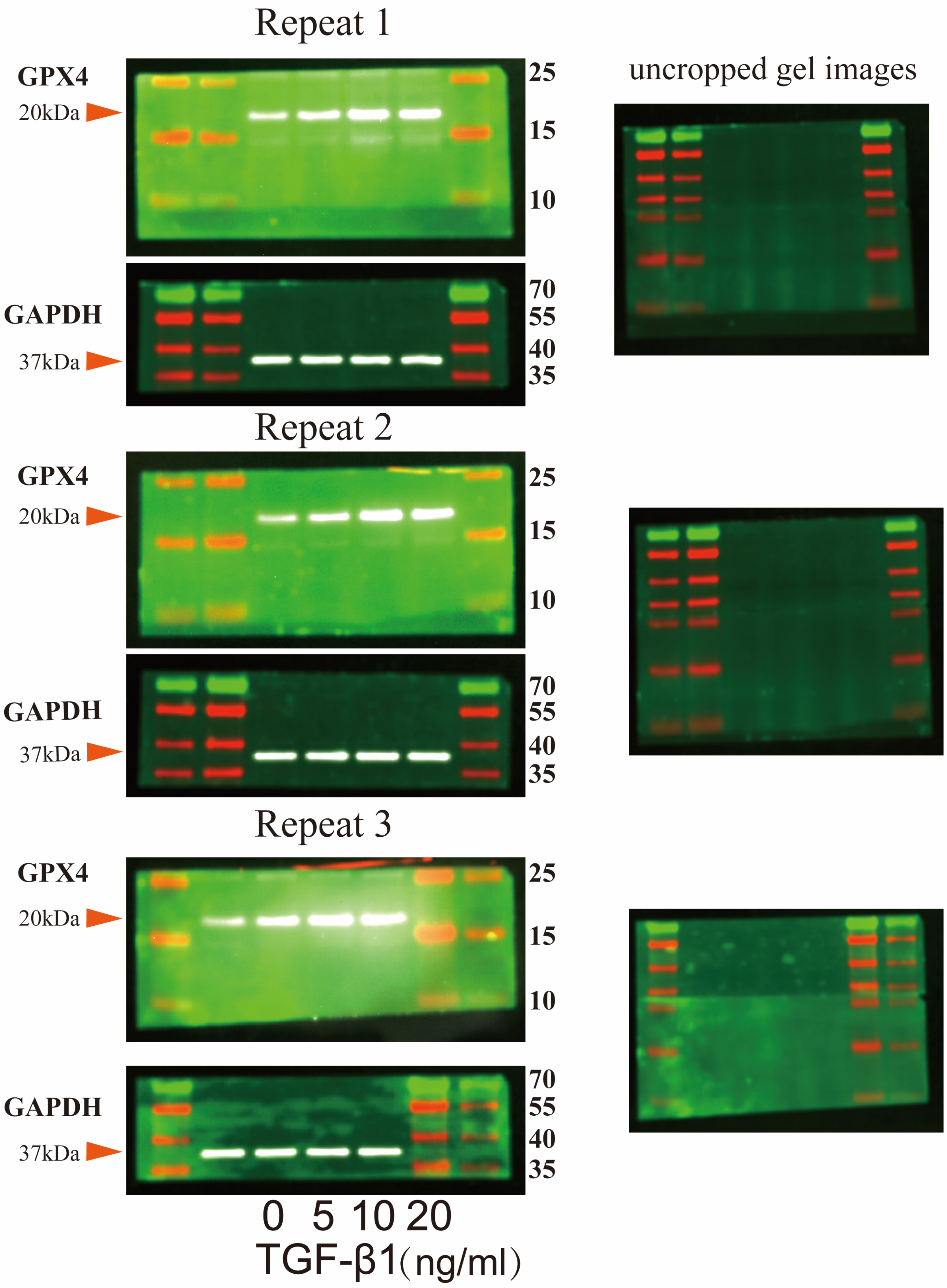

Figure 4D. Example of original western blot for three repeats
T24(left) Smad3 + GAPDH

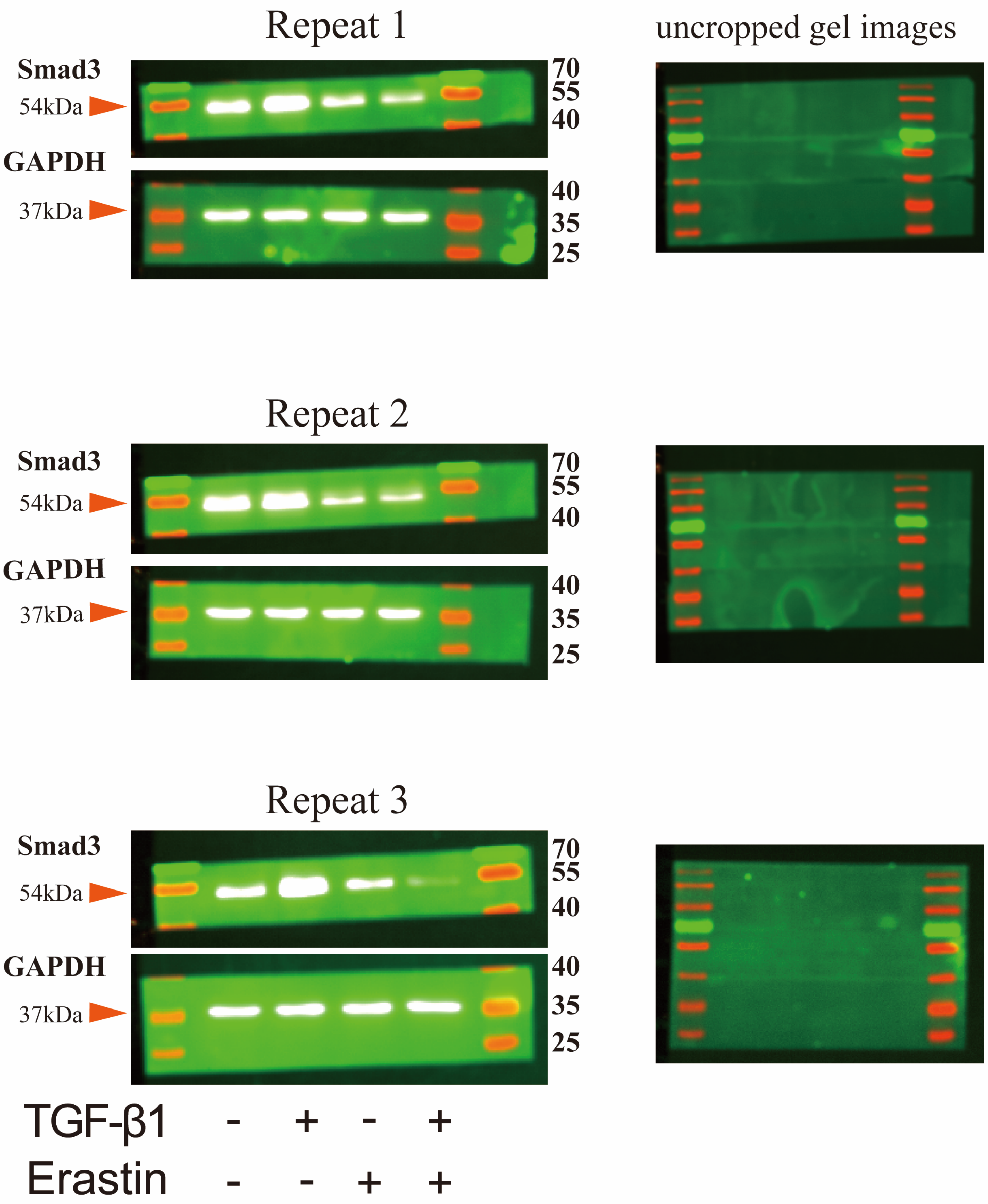

Figure 4D. Example of original western blot for three repeats
5637(right) Smad3 + GAPDH

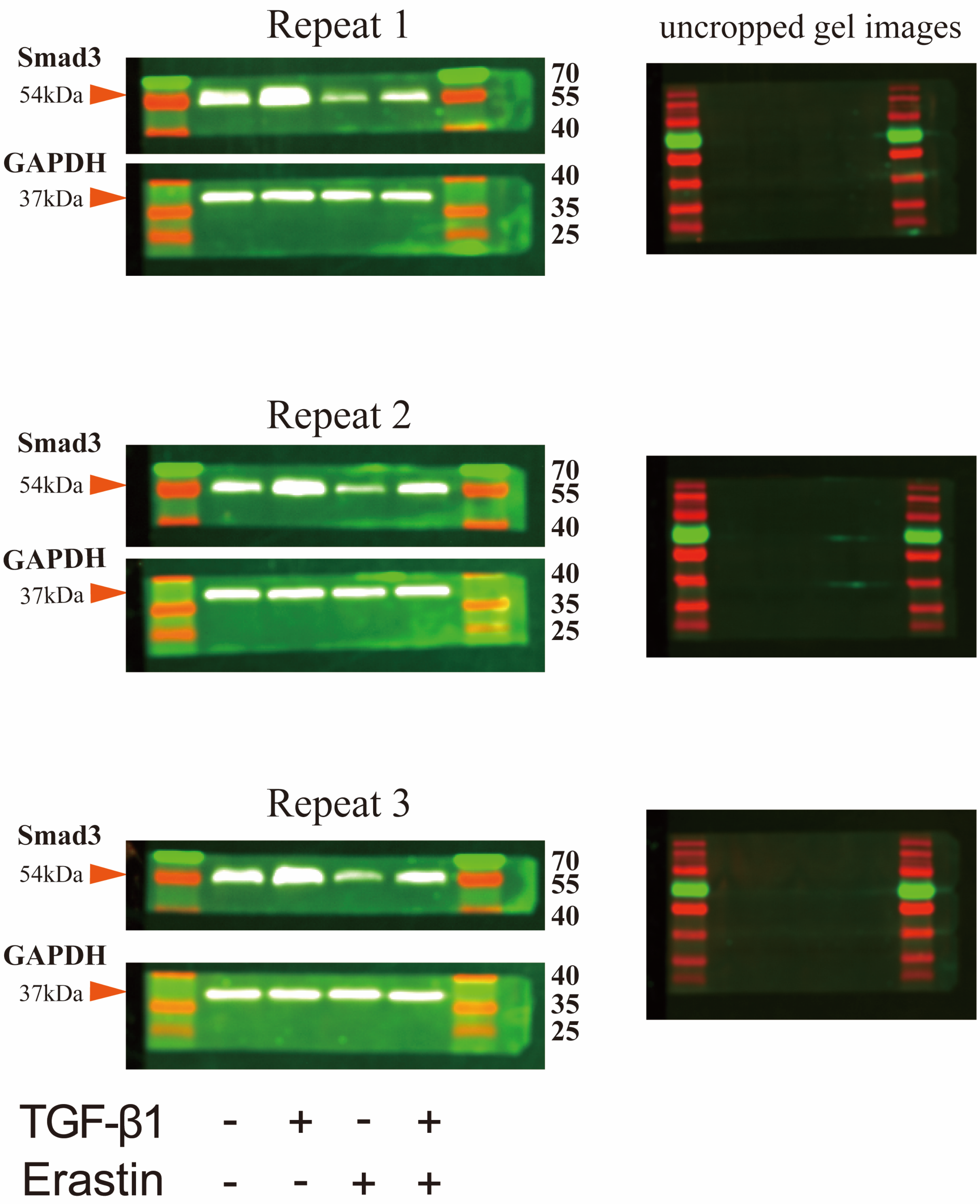

Figure 4I. Example of original western blot for three repeats

T24m(left) Smad3 + p-Smad3 + GAPDH (on the same gel)   5637m(right) Smad3 + p-Smad3 + GAPDH (on the same gel)

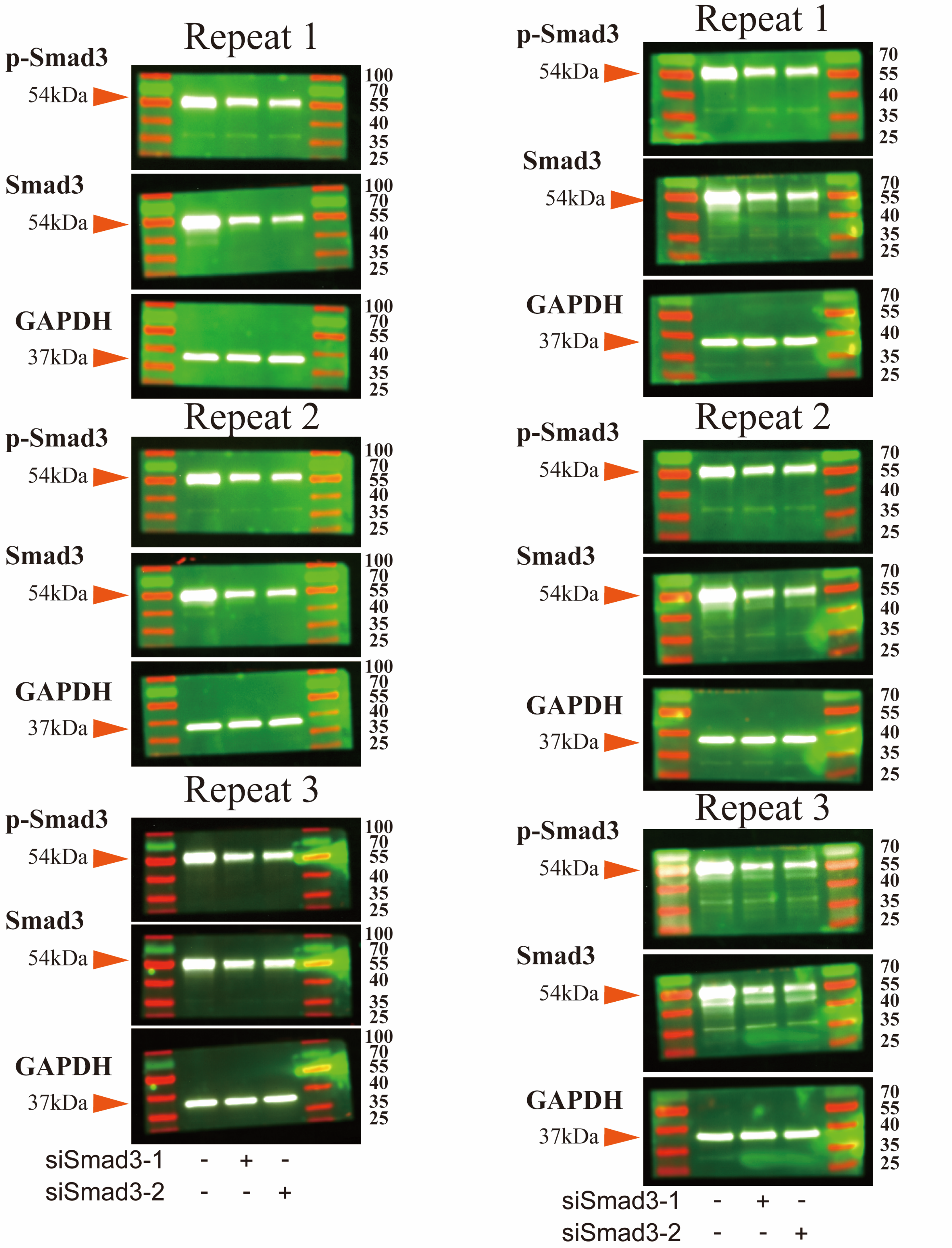

Figure 4J. Example of original western blot for three repeats
T24m(left) GPX4 + GAPDH

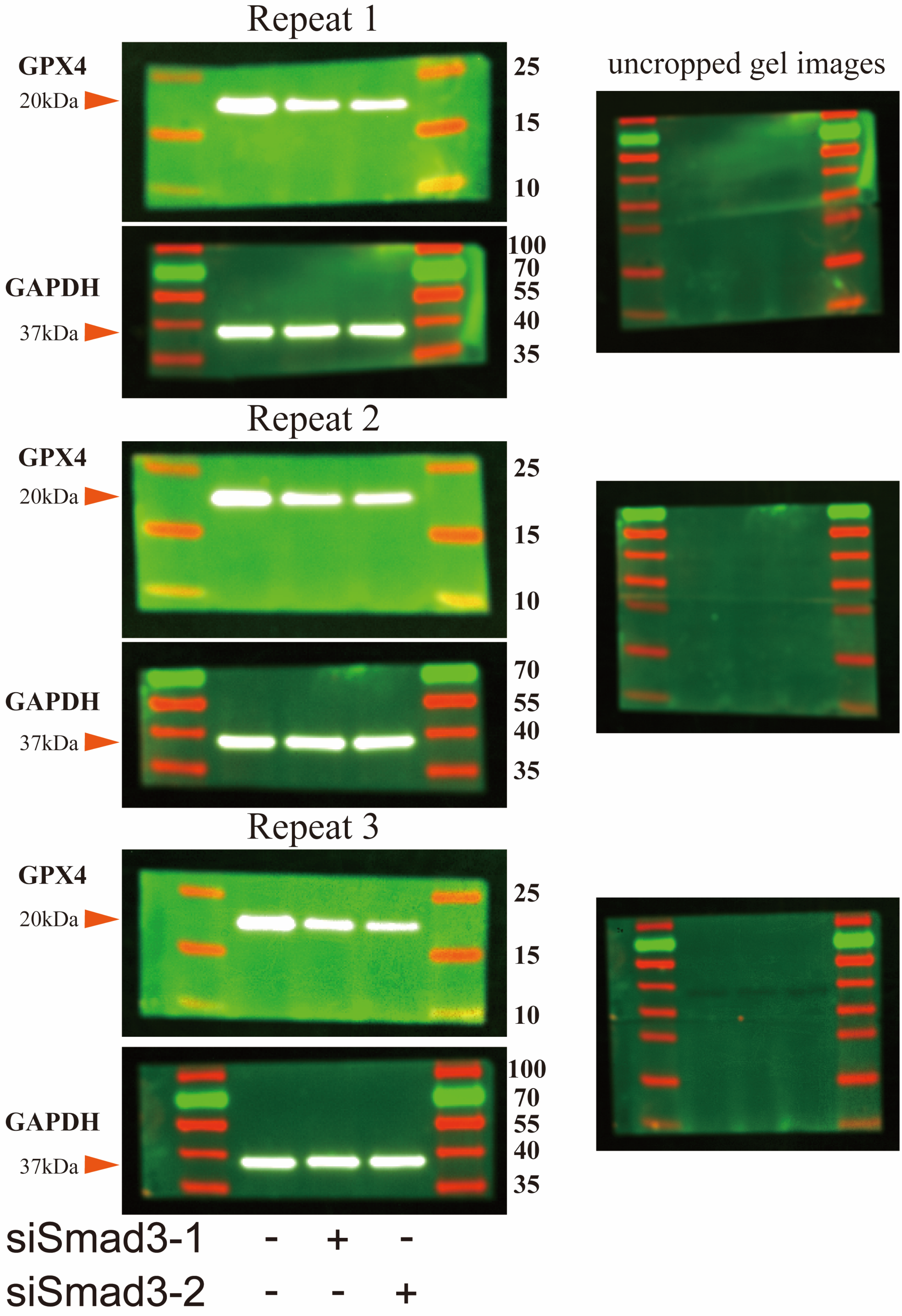

Figure 4J. Example of original western blot for three repeats

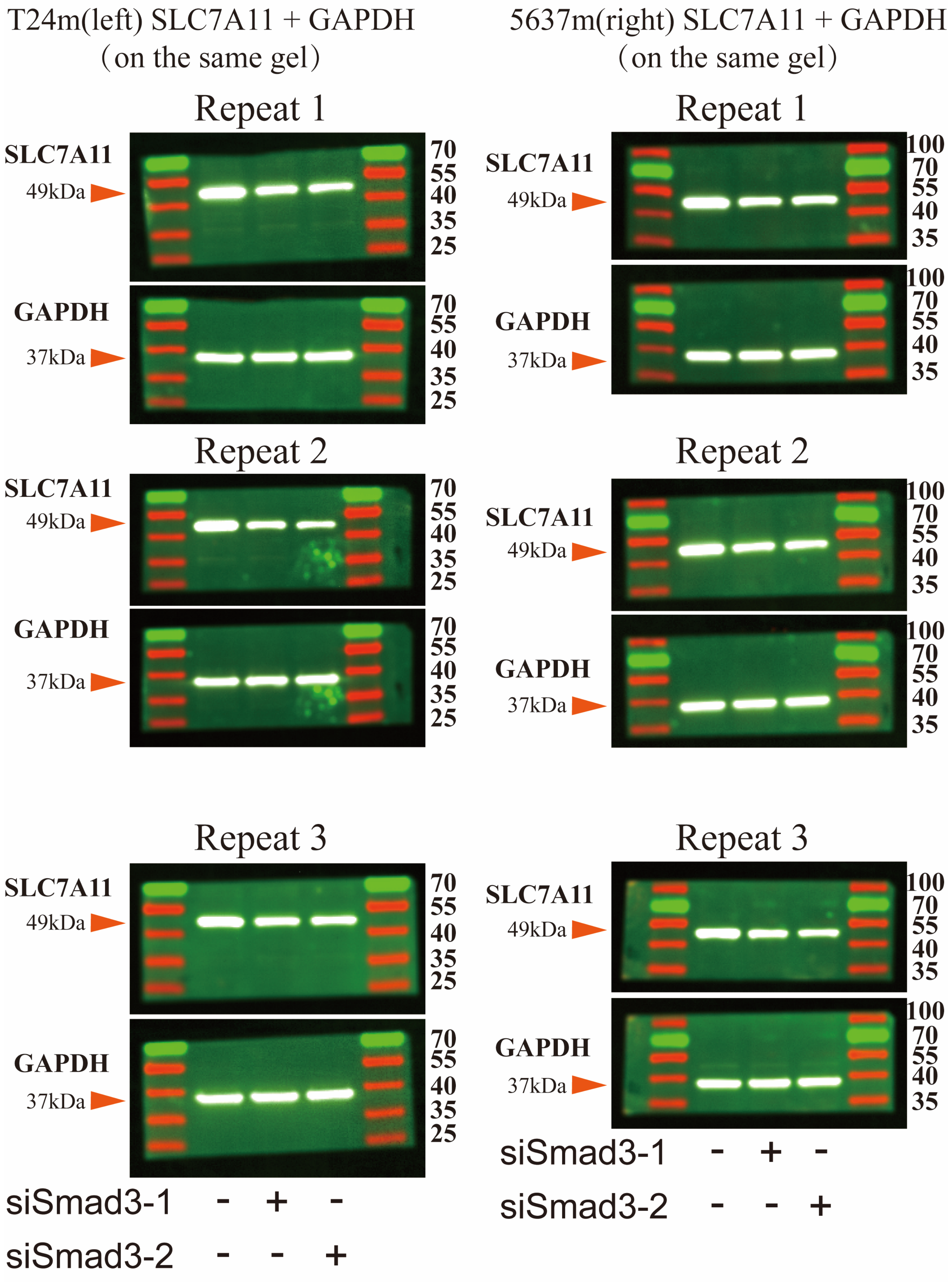

Figure 4J. Example of original western blot for three repeats
5637m(right) GPX4 + GAPDH

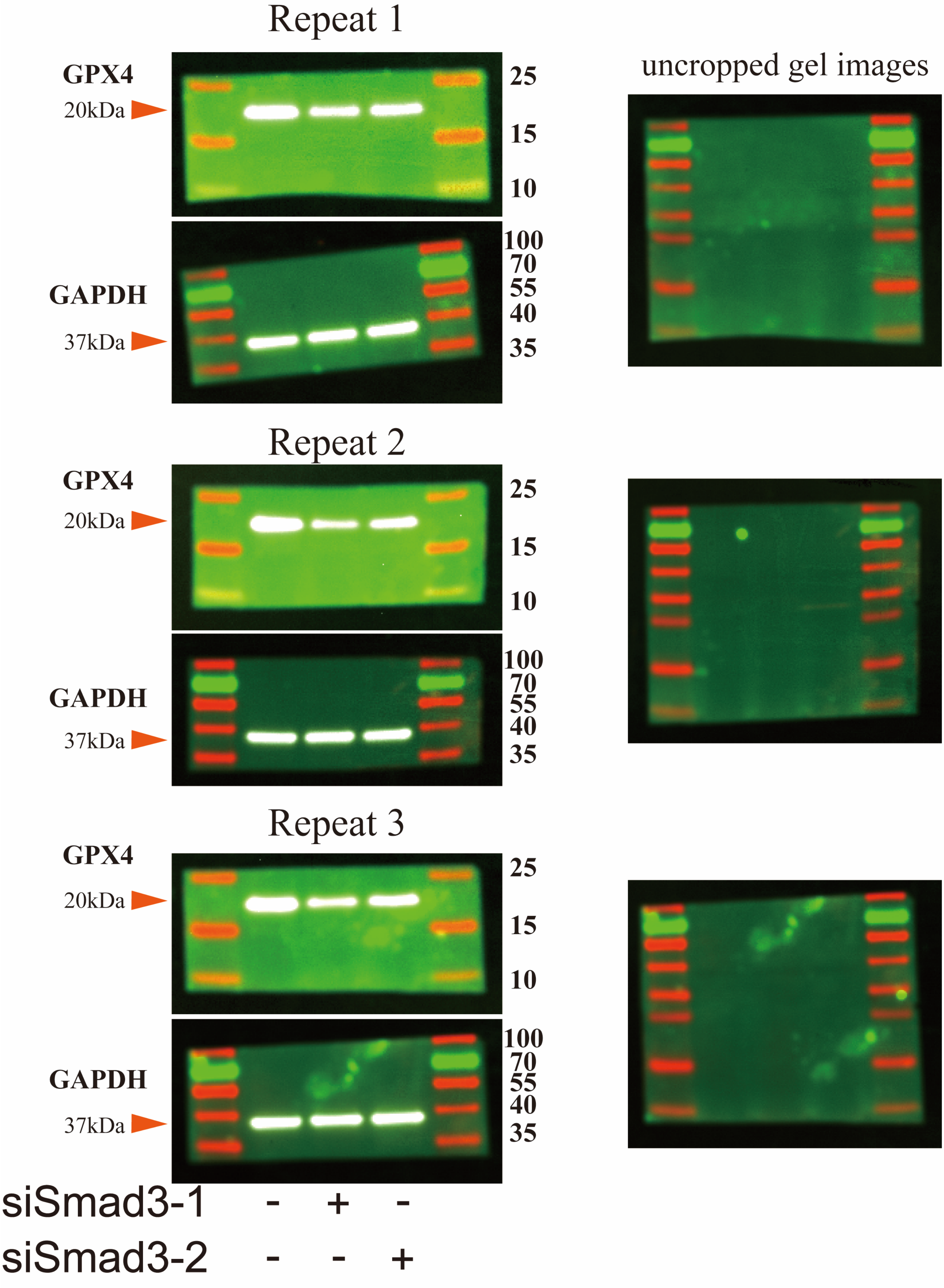

Figure 7L. Example of original western blot for three repeats
T24m(left) CISD2 + GAPDH

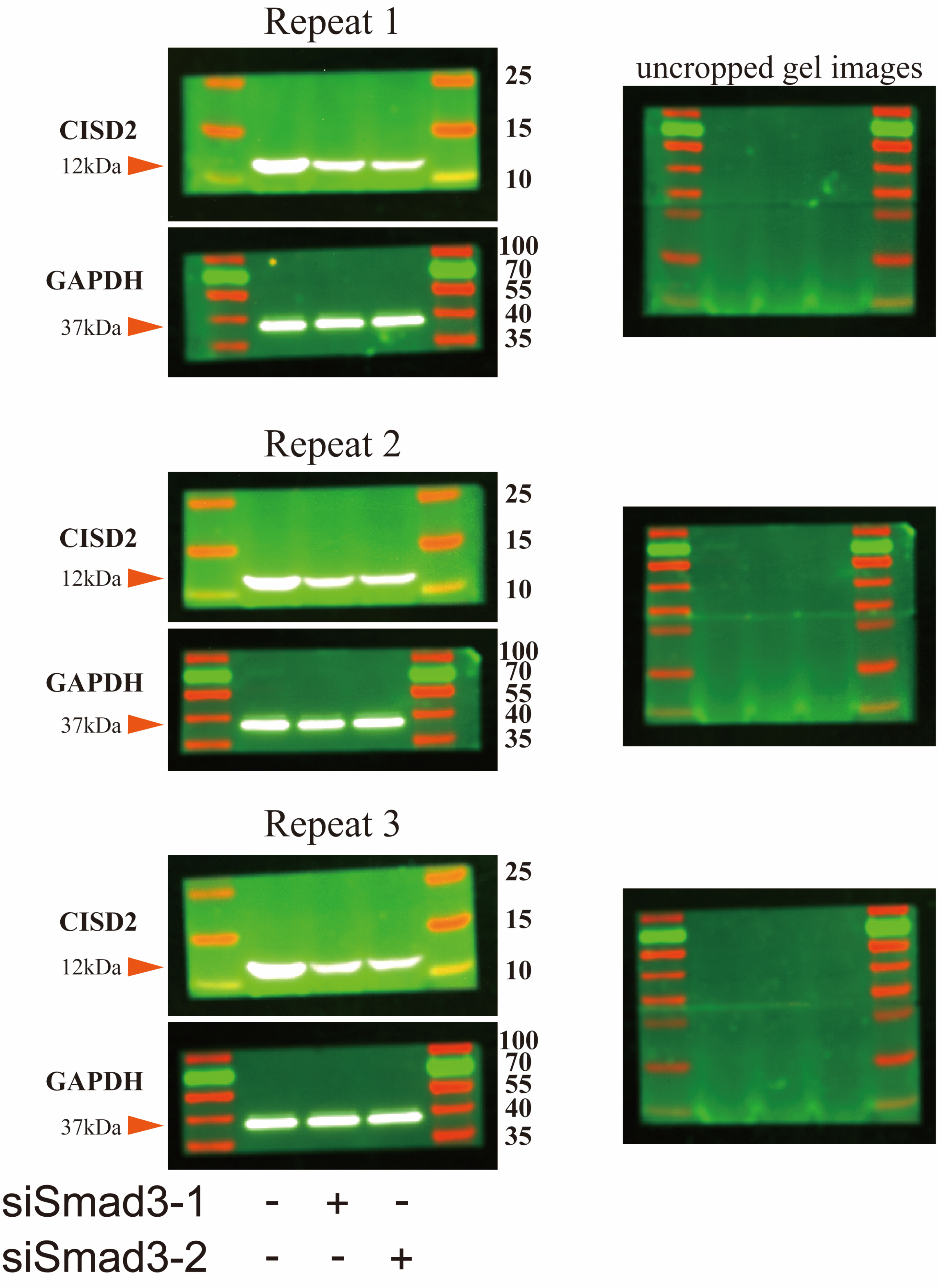

Figure 7L. Example of original western blot for three repeats
5637m(right) CISD2 + GAPDH

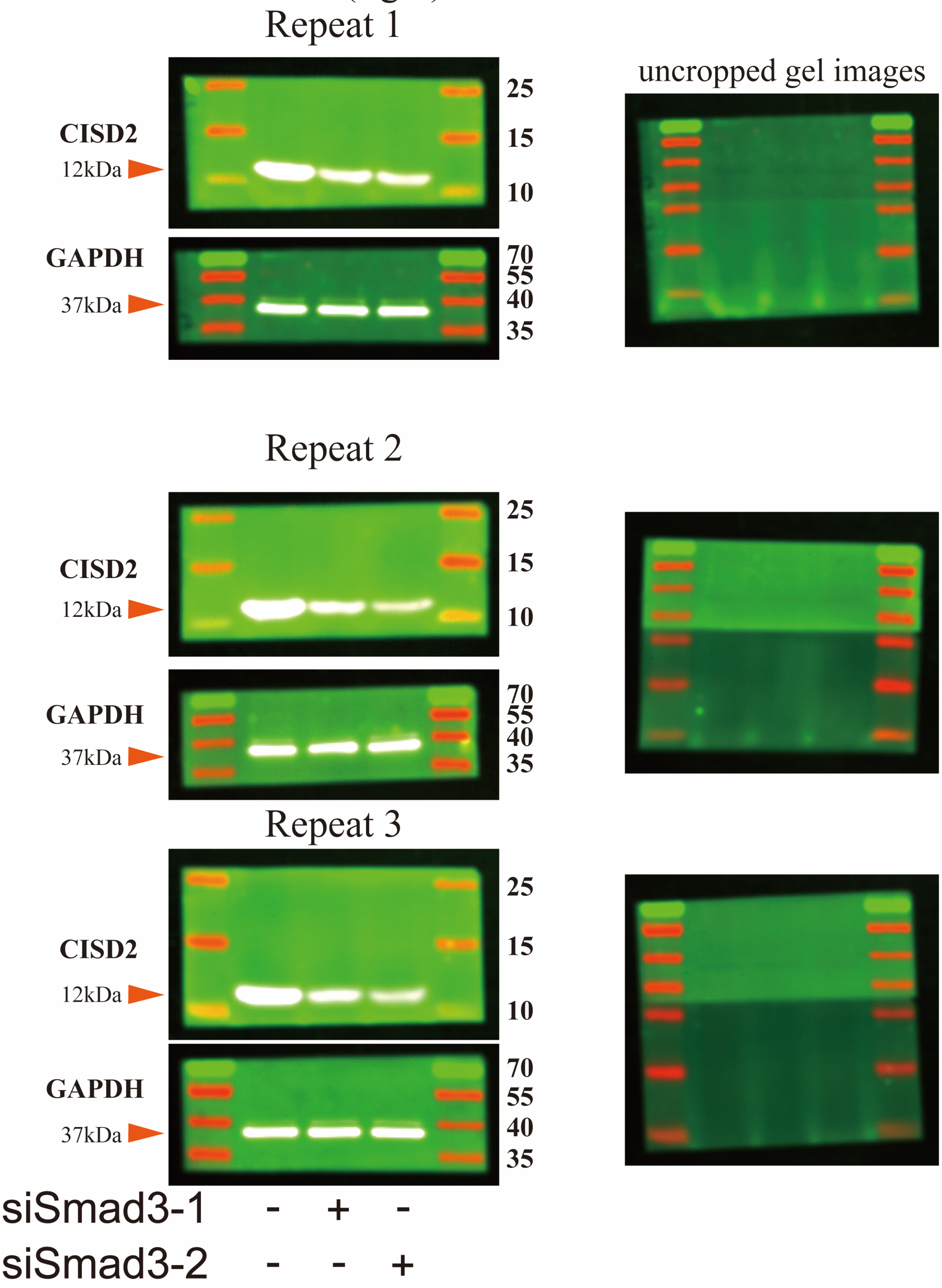

Figure 8B. Example of original western blot for three repeats
T24m(left) CISD2 + GAPDH

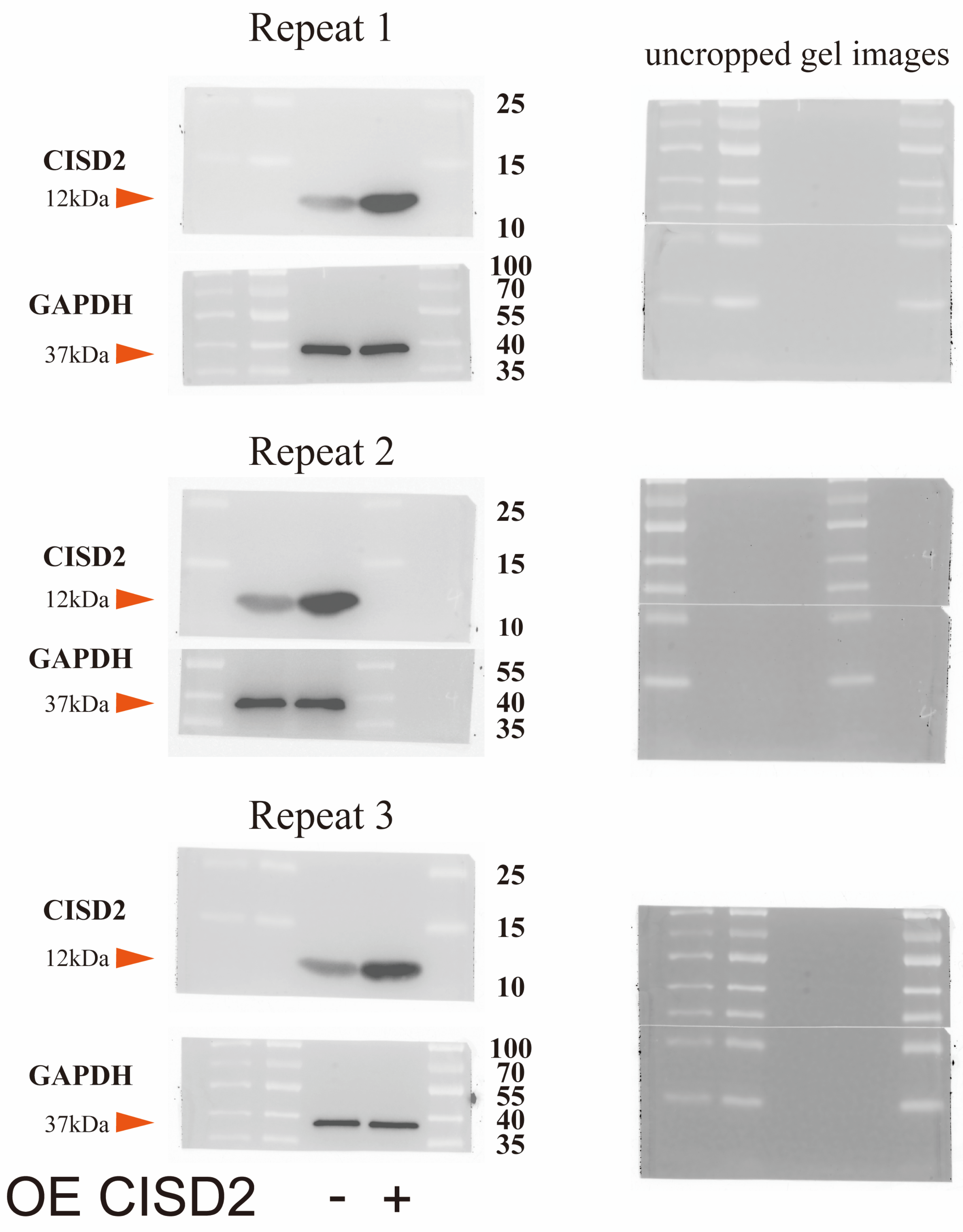

Figure 8B. Example of original western blot for three repeats
5637m(right) CISD2 + GAPDH

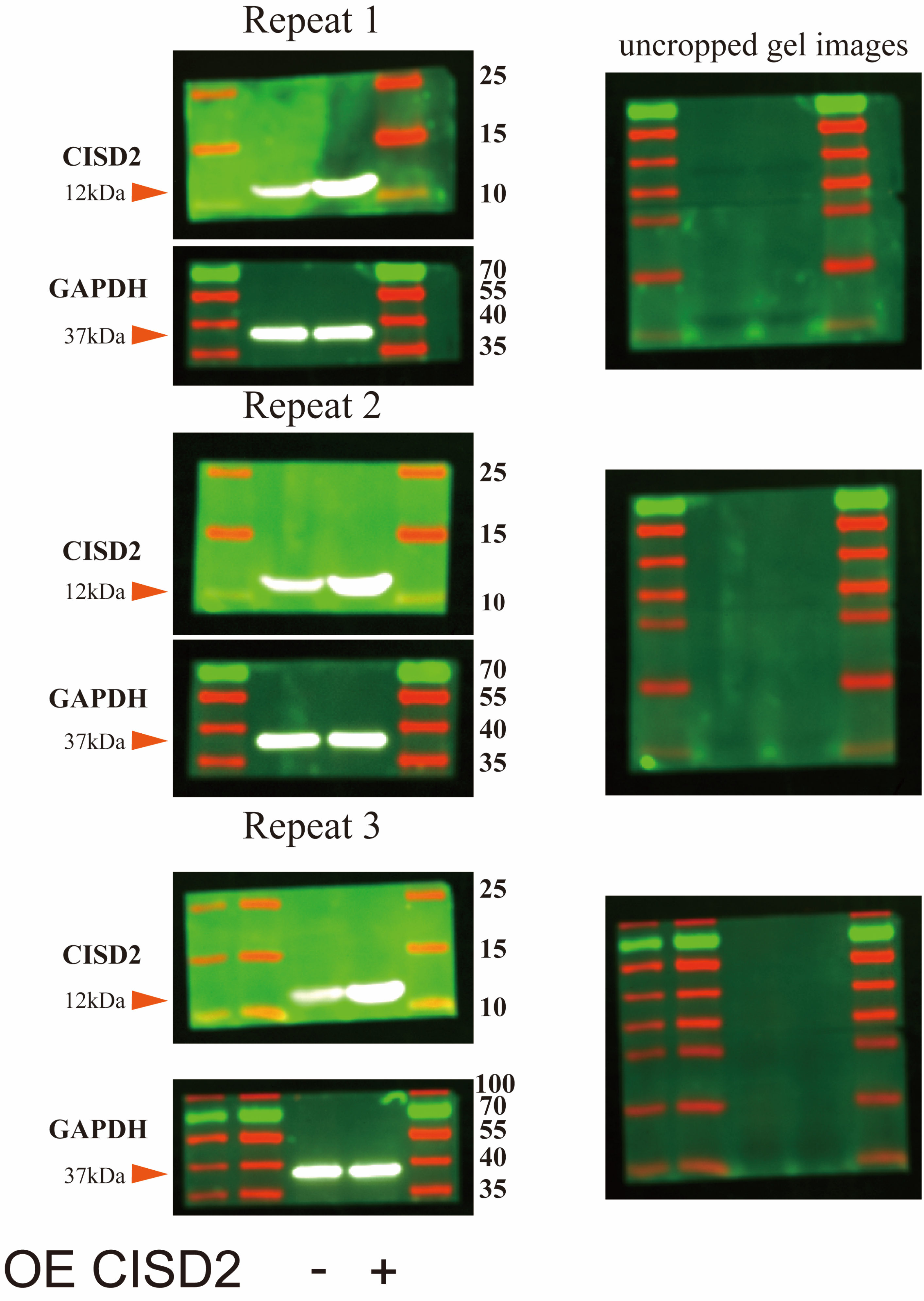

Figure 9B. Example of original western blot for three repeats

T24m Smad3 + p-Smad3 + GAPDH

(on the same gel)

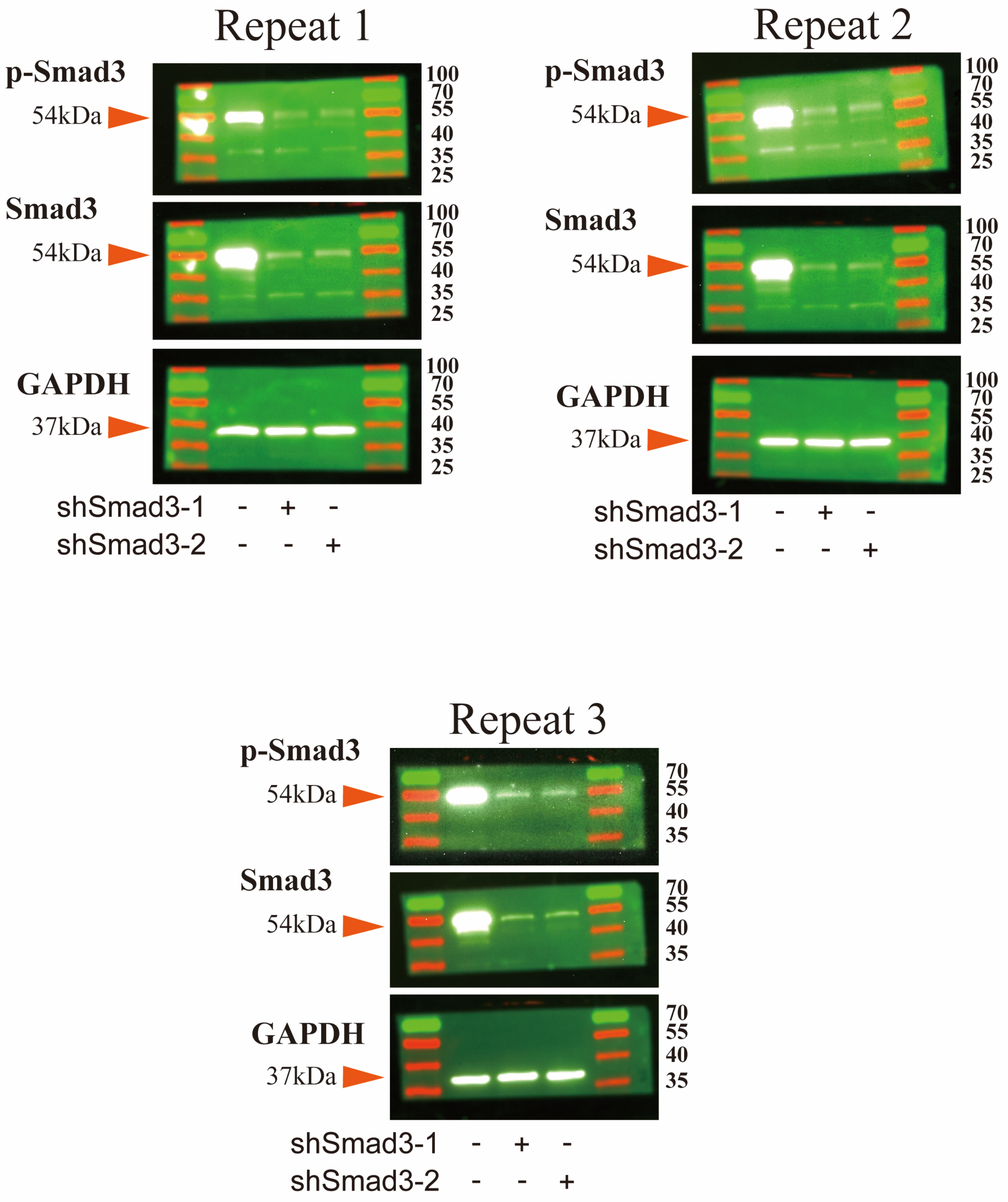

Supplement: Supplementary file 3 — original data [file 41419_2025_8339_MOESM3_ESM.pdf]
