# Supplementary material for: Targeting the SMAD3/CISD2 axis suppresses bladder cancer progression by promoting ferroptosis in mesenchymal-like bladder cancer cells
Source: Cell Death Dis. 2025 Dec 18;17(1):101. doi: 10.1038/s41419-025-08339-9 (PMC12847906; doi:10.1038/s41419-025-08339-9)
Supplement: Supplementary file 5 — Reproducibility Checklist [file 41419_2025_8339_MOESM5_ESM.docx]

**Corresponding Author Name: Weiting Kang and Zilian Cui**

**Manuscript Number: CDDIS-25-2809R**

**Reporting Summary**

***Springer Nature wishes to improve the reproducibility of the work that we publish. This checklist is used to ensure good reporting standards and to improve the reproducibility. Please respond completely to all questions relevant to your***

***manuscript. For more information, please read the journal’s Guide to Authors.***

☑ **Check here to confirm that the following information is available in the Material & Methods section:**

● **The exact sample size (*n)*** for each experimental group/condition, given as a number, not a range

● **A description of the sample collection** allowing the reader to understand whether the samples represent technical or biological replicates (including how many animals, litters, culture, etc.)

● **A statement of how many times the experiment shown was replicated in the laboratory**

● **Definitions of statistical methods and measures**: For small sample sizes (n<5) descriptive statistics are not appropriate, instead plot individual data points

o Very common tests, such as*t*-test, simple χ2 tests, Wilcoxon and Mann-Whitney tests, can be

unambiguously identified by name only, but more complex techniques should be described in the methods section

o Are tests one-sided or two-sided?

o Are thereadjustments for multiple comparisons?

o **Statistical test results**, e.g., ***P* values**

o Definition of **‘center values’** as **median or mean**;

o Definition of **error bars as s.d. or s.e.m. or c.i.**

***Please ensure that the answers to the following questions are reported in the manuscript itself. We encourage you to include a specific subsection in the methods section for statistics, reagents and animal models. Below, provide the***

***page number or section and paragraph number.***

**Statistics and general methods**

1. How was the sample size chosen to ensure

adequate power to detect a pre-specified effect size? (Give section/paragraph or page #)

For animal studies, include a statement about sample size estimate even if no statistical methods were used.

2. Describe inclusion/exclusion criteria if samples or animals were excluded from the analysis. Were

the criteria pre-established? (Give section/paragraph or page #)

3. If a method of randomization was used to

determine how samples/animals were allocated to experimental groups and processed, describe it. (Give section/paragraph or page #)

For animal studies, include a statement about

randomization even if no randomization was used.

**Reported in section/paragraph or page #**

| Reported in Materials and methods, Statistical analysis. |
| --- |
| Reported in Materials and methods, Tumour xenograft experiment and Statistical analysis sections. |
| n/a |
| n/a |
| n/a |


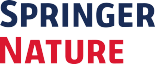


4. If the investigator was blinded to the group

allocation during the experiment and/or when assessing the outcome, state the extent of

blinding. (Give section/paragraph or page #)

For animal studies, include a statement about

blinding even if no blinding was done.

5. For every figure, are statistical tests justified as appropriate?

Do the data meet the assumptions of the tests (e.g., normal distribution)?

Is there an estimate of variation within each group of data?

Is the variance similar between the groups that are

being statistically compared? (Give section/paragraph or page #)

| n/a |
| --- |
| n/a |
| Yes, all statistical tests used in the figures were rigorously selected based on the data characteristics and study design. |
| Yes, the data fully meet the assumptions of all statistical tests applied in this study. |
| Yes, variation within each group is reported for all key analyses. |
| Yes, the variances were similar across all compared groups. |

**Reagents** **Reported** **in** **section/paragraph** **or** **page** **#**

6. Report the source of antibodies (vendor and catalog number)

7. Identify the source of cell lines and report if they were recently authenticated (e.g., by STR

profiling) and tested for mycoplasma contamination

| Reported in Materials and methods, Chemicals and antibodies section, third paragraph. |
| --- |
| Reported in Materials and methods, Cell culture and EMT model section, first paragraph. |

**Animal** **Models** **Reported** **in** **section/paragraph** **or** **page** **#**

8. Report species, strain, sex and age of animals

9. For experiments involving live vertebrates,

include a statement of compliance with ethical regulations and identify the committee(s)

approving the experiments.

| Reported in Materials and methods, Tumour xenograft experiment section. |
| --- |
| Reported in Materials and methods, Tumour xenograft experiment section. |

10. We recommend consulting the ARRIVE guidelines [(](http://www.ncbi.nlm.nih.gov/pubmed/20613859)*[PLoS Biol.](http://www.ncbi.nlm.nih.gov/pubmed/20613859)* **[8](http://www.ncbi.nlm.nih.gov/pubmed/20613859)**[(6), e1000412,2010)](http://www.ncbi.nlm.nih.gov/pubmed/20613859) to ensure that other relevant aspects of animal studies are adequately reported.

*2*

**Human** **subjects**

11. Identify the committee(s) approving the study protocol.

12. Include a statement confirming that informed consent was obtained from all subjects.

13. For publication of patient photos, include a

statement confirming that consent to publish was obtained.

14. Report the clinical trial registration number (at [ClinicalTrials.gov](http://clinicaltrials.gov/)or equivalent).

**Reported** **in** **section/paragraph** **or** **page** **#**

| Reported in Materials and methods, Clinical specimens. |
| --- |
| Reported in Materials and methods, Clinical specimens. |
| n/a |
| n/a |

15. For phase II and III randomized controlled trials, please refer to the[CONSORT statement](http://www.consort-statement.org/)and submit the CONSORT checklist with your submission.

16. For tumor marker prognostic studies, werecommend that you follow the[REMARK reporting guidelines.](http://www.nature.com/nrclinonc/journal/v2/n8/full/ncponc0252.html)

**Data** **deposition** **Reported** **in** **section/paragraph** **or** **page** **#**

Reported in Materials and methods, RNA

sequencing and Bioinformatics analysis.

17. Provide accession codes for deposited data. Data deposition in a public repository is

mandatory for:

a. Protein, DNA and RNA sequences

b. Macromolecular structures

c. Crystallographic data for small molecules

d. Microarray data

Deposition is strongly recommended for many other datasets for which structured public repositories exist; more

details on our data policy are available in the Guide to Authors. We encourage the provision of other source data in supplementary information or in unstructured repositories such as[Figshare](http://www.figshare.com/)and[Dryad.](http://datadryad.org/) We encourage

publication of Data Descriptors (see[Scientific Data)](http://www.nature.com/sdata/) to maximizedatareuse.

18. If computer code was used to generate results that are central to the paper’sconclusions,

n/a

include a statement in the Methods section

under “**Code** **availability”** to indicate whether and how the code can be accessed. Include

version information as necessary and any restrictions on availability.

*3*
